# Synthesis, molecular modelling and evaluation of larvicidal efficacy of annulated Benzo[*h*]chromenes against *Culex pipiens* L. Larvae

**DOI:** 10.1038/s41598-024-68035-0

**Published:** 2024-08-08

**Authors:** Mahmoud K. F. El-Sayed, Manal M. Elshahawi, Fatma S. M. Abu El-Azm, Eslam M. Hosni, Mahmoud Kamal, Yasmeen M. Ali

**Affiliations:** 1 Chemistry Department, Faculty of Science, Ain Shams University, Cairo, 11566 Egypt; 2 Entomology Department, Faculty of Science, Ain Shams University, Cairo, 11566 Egypt

**Keywords:** Benzo[h]chromene, Benzochromenopyrimidine, Benzochromenotriazolopyrimidine, *Culex pipiens*, Disease vector, Larvicidal efficacy, Biochemistry, Chemistry

## Abstract

A new series of substituted benzo[h]chromene, benzochromenopyrimidine, and benzochromenotriazolopyrimidine derivatives were synthesized via chemical transformations of iminonitrile, ethoxymethylene amino, and cyanomethylene functionalities. The chemical structures of the synthesized compounds were assured by spectroscopic data and elemental analysis. The larvicidal efficacy of these compounds against *Culex pipiens L*. larvae was investigated, revealing potent insecticidal activity, particularly for compounds **6**, **10**, and **16**, exceeding that of the standard insecticide chlorpyrifos. The mode of action of these compounds was explored through molecular docking studies, indicating their potential as acetylcholine esterase (AChE) inhibitors and nicotinic acetylcholine receptors (nAChR) blockers. The structure–activity relationship analysis highlighted the influence of substituents and fused heterocyclic rings on larvicidal potency. These findings suggest that the synthesized compounds hold promise as potential candidates for developing novel and effective mosquito control agents.

## Introduction

Benzochromene derivatives have demonstrated a wide range of biological activities, including antimicrobial, antioxidant, anti-inflammatory, analgesic, anti-proliferative, and anti-tubercular properties^1–7^. Among these, benzochromenopyrimidines are particularly interesting due to their antimicrobial, analgesic, anti-inflammatory, and anticancer effects^8–13^. Notably, some benzochromenopyrimidine derivatives act as acetylcholinesterase inhibitors, potent antioxidants used in Alzheimer's disease treatment, and antidyslipidemic agents^14,15^. Additionally, 1,2,4-triazolopyrimidines, a type of hybrid heterocycle, have displayed antitumor, antimicrobial, and antiviral activities^16–19^.

*Culex pipiens*, a globally distributed mosquito species, poses a significant public health threat as a vector for various arboviruses, including West Nile, Saint Louis encephalitis, Eastern equine encephalitis, and Usutu viruses^20–23^. Additionally, it transmits filariasis and avian malaria, and recent studies suggest it may contaminate raw milk with harmful microbial pathogens^20,24^. The ability of *C. pipiens* to transmit various diseases to both humans and animals, coupled with its widespread distribution and increasing resistance to conventional insecticides, underscores the importance of developing new and effective control strategies to mitigate its impact on public health and agriculture^20,25^.

The escalating issue of insecticide resistance in disease vectors, such as *C. pipiens*, necessitates the urgent development and evaluation of novel insecticidal compounds to overcome this challenge^25^. The exploration of heterocyclic compounds, including pyrano[2,3-c]pyrazoles, as a source of novel insecticides has shown promise, as demonstrated by El-Sayed et al. (2023)^26^, who reported the larvicidal activity of these derivatives against *C. pipiens* and *Musca domestica*. This highlights the potential of heterocyclic compounds, such as benzochromenes, as a promising avenue for developing effective insecticidal agents. Previous studies have shown that some benzochromenopyrimidine derivatives act as acetylcholinesterase inhibitors^14^, further supporting the rationale for investigating the insecticidal potential of benzochromenes and their derivatives.

Insecticides are classified based on how they work, including nerve poisons, growth regulators, chitin synthesis inhibitors, and juvenile hormone mimics^27^. Nerve poisons disrupt the insect's nervous system through various mechanisms. One group of nerve poisons are acetylcholinesterase (AChE) inhibitors. These insecticides, such as the organophosphate chlorpyrifos, prevent the breakdown of the neurotransmitter acetylcholine, leading to its buildup and overstimulation of the nervous system^28,29^. Neonicotinoids, like nitenpyram, target nicotinic acetylcholine receptors (nAChRs), causing prolonged activation and ultimately paralysis^30,31^. Another class of nerve poisons includes pyrethroids and indoxacarb. These insecticides disrupt voltage-gated sodium channels (VGSCs), which are crucial for nerve signal transmission^32^. This disruption leads to hyperexcitation of nerve cells, eventual paralysis, and ultimately insect death^27^.

Building upon our previous research on the synthesis of heterocyclic systems with biological applications^11,12,26,33–38^, we report the synthesis of fused benzochromene, benzochromenotriazolopyrimidine, and benzochromenopyrimidine systems. The primary objective of this study is to investigate the insecticidal potential of these compounds against *C. pipiens* immatures. A molecular docking analysis will complement the larvicidal bioassay to elucidate the potential mode of action of these compounds, contributing to our understanding of any resulting toxicity in the biological assay.

## Results and discussion

### Chemistry

**Scheme 1:**
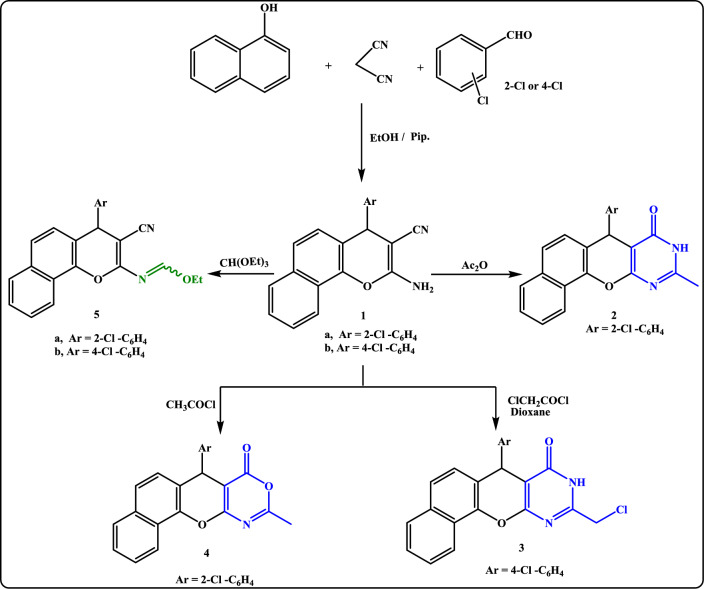
Treatment of *β*-enaminonitrile **1a, b** with some carbon electrophiles.

In our running study, the intended starting compounds 2-amino-4-aryl-4*H*-benzo[*h*]chromene-3-carbonitrile **1a**, **b** were formerly synthesized via a multi-component reaction of malononitrile, 2-chlorobenzaldehyde (or 4-chlorobenzaldehyde) and α-naphthol in ethanol containing a catalytic amount of piperidine (Scheme 1). The structures of **1a** and **1b** were inferred from m.p and typical IR data with those reported in the literature^39,40^.

The functionalities in *β*-enaminonitrile **1a,b** rendered it a beneficial precursor for the creation of new heterocyclic compounds. Thus, refluxing *β*-enaminonitrile **1a**,**b** with some appointed carbon electrophiles such as acetic anhydride and/or chloroacetyl chloride afforded pyrimidinone derivatives **2** and/or **3**, respectively (Scheme 1). The IR, ^1^H-NMR, and ^13^C-NMR spectra as well as the elemental analyses were completely in accord with those assigned structures. (cf. experimental).

The behavior of the understudy enaminonitrile **1a** towards acetyl chloride was investigated (Scheme 1), it was reported that the treatment of enaminonitrile with acid chloride afforded either acetamide derivative^41^ or pyrimidinone derivative^42^, but herein none of the expected structures have been isolated. The isolated product was identified as 7-(2-chlorophenyl)-10-methyl-7*H*,11*H*-dihydro-7*H*,8*H*-benzo^7,8^ chromeno[2,3-*d*][1,3]oxazin-8-one **(4)**. The IR spectrum of** 4** revealed the absence of an absorption band of the nitrile group and the presence of an absorption band at 1732, and 1656 cm^−1^ attributed to C=O of oxazinone and C=N groups, respectively. Moreover, the ^13^C-NMR spectrum of **4** exhibited signals for the carbonyl group and C_2_ of the oxazinone ring.

**Figure 1:**
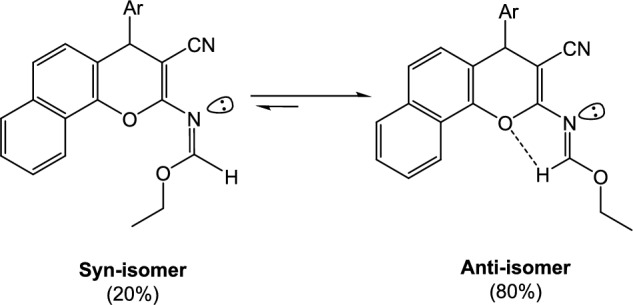
Two diastereomeric isomers of compound **5a**.

Moreover, condensation of enaminonitrile **1a, b** with triethyl orthoformate yielded the corresponding ethyl formimidate **5a, b** (Scheme 1), which was subsequently exploited as the principal intermediate for the synthesis of novel chromenopyrimidines and chromenotriazolopyrimidines via reaction with diverse nitrogen nucleophiles. The ^1^H-NMR spectrum of **5a**, **b** showed the presence of triplet and quartet signals referring to the existence of protons of an ethoxy group. The ^1^H-NMR spectrum of **5a** showed its presence as two diastereomers syn and anti isomers with predominant of anti-isomer due to the presence of H-bonding is shown **(**Fig. 1**)**.

**Scheme 2:**
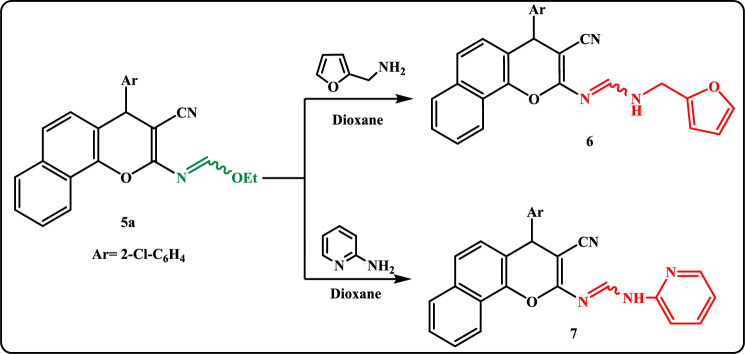
Reaction of ethyl formimidate **5a** with heteroamines.

Aminolysis of the formimidate **5a** with heteroamines either 2-furanylmethanamine or 2-aminopyridine in boiling dioxane yielded the formamidine derivatives **6** and **7**, respectively (Scheme 2). The evidence for the uncyclized structures **6** and **7**, was deduced from the IR spectrum which showed an absorption band at 2191 cm^-1^ corresponding to (C≡N) in addition to the appearance of singlet signals for NH proton exchangeable with D_2_O in ^1^H-NMR spectra.

**Scheme 3:**
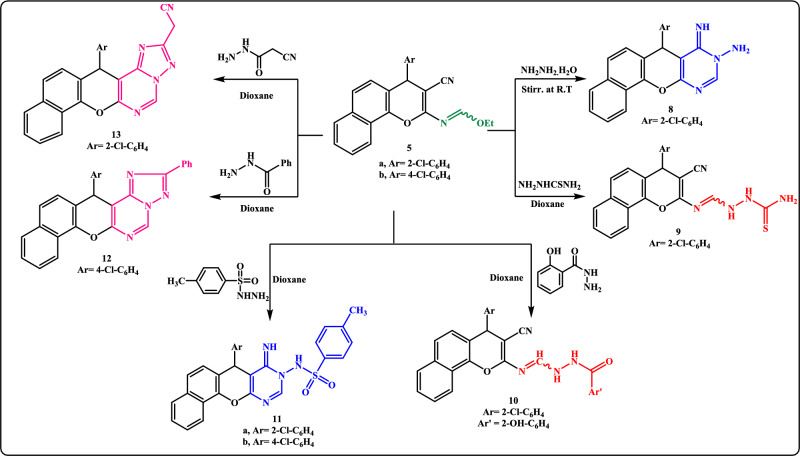
Reaction of formimidate **5a, b** with hydrazine hydrate and some hydrazine derivatives.

Meanwhile, the reactivity of ethyl formimidate **5a** with hydrazine hydrate and some hydrazine derivatives has been reported (Scheme 3). So, stirring ethyl formimidate **5a** with excess hydrazine hydrate for 10 h at room temperature yielded the amino-imino derivative **8**. The structure of **8** was established from spectral data. The IR spectrum of **8** showed the disappearance of *ν*C≡N in addition to the appearance of *ν*NH, *ν*NH_2,_ and *ν*C=N at 3337, 3319, 3285, and 1653 cm^−1^, respectively. Moreover, appearance of two singlet signals for NH, NH_2_ exchangeable with D_2_O besides a singlet signal at δ 8.25 ppm for C_2_ pyrimidine-H in addition to the disappearance of the triplet and quartet signals attributed to the ethyl protons of its precursor **5a** in ^1^H-NMR (DMSO-*d6*) spectrum reinforces the proposed structure.

The uncyclized product, 1[{4-(2-chlorophenyl)-3-cyano-4*H*-benzo[*h*]chromen-2-ylimino}-methyl]thiosemicarbazide** 9** was obtained as brown crystals on conducting ethyl formimidate **5a** with thiosemicarbazide in dioxane. Likewise, condensation of compound **5a** with 2-hydroxy-benzohydrazide in dioxane gave benzo[*h*]chromene benzohydrazide derivative **10**. (Scheme 3) Moreover, when formimidate derivatives **5a, b** were allowed to react with *p*-toluene sulphonohydrazide in refluxing dioxane, benzo[7,8]chromeno[2,3-*d*]pyrimidine derivatives **11a,b** were afforded. (Scheme 3).

**Figure 2:**
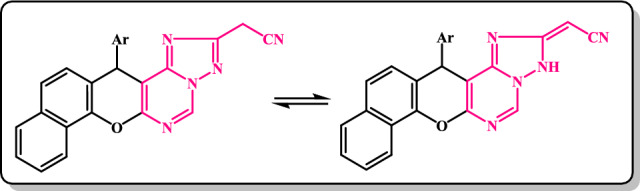
Two isomeric forms of compound **13**.

On the other hand, it has been mentioned that ethyl formimidate derivatives reacted with acid hydrazides through two-ring annulations to give fused triazolopyrimidine systems^43,44^. Thus, triazolo[1,5-c]pyrimidine derivatives **12** and **13** were isolated via the treatment of ethyl formimidate **5b, 5a** with benzoylhydrazide and 2-cyanoacetic acid hydrazide in refluxing dioxane (Scheme 3). A supportive clue for the suggested structure **12** was upcoming from its IR spectrum as it lacked absorption bands for both cyano-, carbonyl, and NH groups and showed only absorption bands for CH-Ar at 3046 cm^−1^ and C=N at 1619 cm^-1^. Moreover, the spectral data were utilized to deduce the structure of **13** and showed its existence in two tautomeric forms (Fig. 2). Thus, the IR spectrum displayed absorption bands for *ν*NH*, ν*C≡N, and *ν*C=N at 3340, 2261(small), 2192, 1658, and 1627 cm^−1^, respectively. Strong support for structure **13** was forthcoming from its ^1^H-NMR (DMSO-*d*_*6*_) spectrum which disclosed the disappearance of the triplet and quartet signals assigned to the ethyl protons of its precursor **5a** and showed the appearance of signals at *δ*(ppm): 3.58 (s, 2H, CH_2_CN), 4.39 (s, 1H, NH, exchangeable with D_2_O), 5.40 (s, 1H, C_4_-pyran), 6.27 (s, 1H, =CH), 6.98–8.23 (m, 10H, Ar–H), 9.73 (s, 1H, pyrimidine-H).

**Scheme 4:**
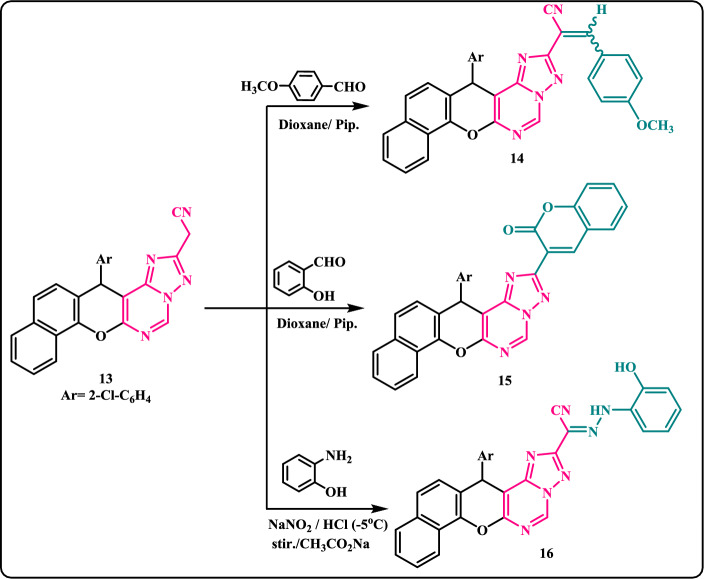
Reaction of cyanomethyl derivative **13** with aromatic aldehydes and diazonium salt.

The reactivity of cyanomethyl functionality towards aromatic aldehydes as electrophilic reagents has been investigated by many researchers to insert and construct new heterocyclic systems and it was found to give generally Knoevenagel condensation product under basic conditions and yielded either 2-iminochromene^45,46^ or 2-chromenones^47^ in case of salicylaldehyde. Thus, the reaction of cyanomethyl derivative **13** with *p*-methoxybenzaldehyde and/or salicylaldehyde in refluxing dioxane in the presence of a catalytic amount of piperidine afforded arylidene derivative **14** and chromenone derivative **15**, respectively (Scheme 4). ^1^H-NMR (DMSO‐d_6_) spectrum of **14** disclosed the disappearance of any bands for CH_2_-group of its precursor **13** and displayed bands at δ(ppm): 3.83 (s, 3H, OCH_3_), 6.24 (s, 1H, C_4_-pyran), 7.08–7.93 (m, 13H, Ar–H), 8.30 (m, 1H, Ar–H), 9.69 (s, 1H, pyrimidine-H) in addition to a new singlet band at *δ* 8.15 ppm due to the presence of benzylidene CH.

On the other hand, the elimination of the 2-iminochromene structure and the preference of the 2-chromenone structure for compound **15** were deduced from spectral data. Thus, the IR spectrum of **15** revealed the absence of stretching bands for the C≡N or NH groups, and the presence of a stretching band at 1747 cm^−1^ attributed to an oxo-coumarin group. Strong evidence for the structure **15** came from ^1^H-NMR (DMSO‐d_6_) which lacked the existence of either methylene protons of its precursor **13** or any signal attributed to NH proton and showed bands at δ(ppm): 6.32 (s, 1H, C_4_-pyran), 7.01–8.34 (m, 14H, Ar–H), 8.63 (s, 1H, chromenone ring), 8.31 (d, 1H, Ar–H), 9.77 (s, 1H, pyrimidine-H).

Meanwhile, the reactivity of cyanomethyl functionality towards diazonium salts as nitrogen electrophilic reagents was also studied. Thus, treatment of cyanomethyl derivative **13** with 2-hydroxy-benzenediazonium chloride in the presence of sodium acetate at − 5 °C yielded the hydrazonyl derivative **16**. Adequate evidence for the chemical structure of **16** was substantiated by the correct elemental analysis and spectral data.

### Biological evaluation

#### Larvicidal activity

Given the reported activity of benzochromenopyrimidine derivatives as acetylcholinesterase (AChE) inhibitors^14^ and pyranopyrazole derivatives as potent insecticidal nicotinic acetylcholine receptor (nAChR) activators^26^, we evaluated fifteen synthesized benzochromene, benzochromenotriazolopyrimidine, and benzochromenopyrimidine derivatives against *C. pipiens* larvae as potential neurotoxic insecticides.

In our study, all fifteen tested compounds demonstrated insecticidal activity (Table 1) against the third larval instar of the *C. pipiens* lab strain, inducing neurotoxic symptoms such as tremors, uncoordinated movements, paralysis, and finally larvae death. This observation underscores the potential action of these compounds as nerve poisons that may act on the neural receptors (AChE, nAChRs, or VGSC α subunit) of mosquito larvae. Notably, all compounds were more potent than the standard insecticide **Chlorpyrifos** under identical experimental conditions, supporting the hypothesis of their action as neurotoxic insecticides. Chlorpyrifos was chosen as a reference compound due to its widespread use as a conventional insecticide and its known activity as an acetylcholinesterase inhibitor. While it may not share structural similarities with the synthesized compounds, it provides a benchmark for comparing the larvicidal potency of the novel compounds.

**Table 1:** The average insecticidal activity for 15 tested compounds against the third larval instar of *C. pipiens* after 12 h. compared to the insecticidal activity of a conventional insecticide **“Chlorpyrifos”** at the same conditions. Confidence Interval (C.I) of 95%.

Compound No	LC_25_/ mg/mL ± SD	LC_50_/ mg/mL ± SD	LC_90_/ mg/mL ± SD	χ^2^cal./tab_. (7.8)_	r^2^	P value	Toxicity index
**1a**	169.2 ± 0.6	334.3 ± 0.1	1219.4 ± 0.22	2.4	0.87	0	23.3
**1b**	220.9 ± 0.4	361.8 ± 0.2	923.1 ± 0.26	1.1	0.94	0.009	21.5
**3**	217.7 ± 0.9	378.8 ± 0.12	1084.7 ± 0.14	0.9	0.99	0.008	20.5
**5a**	190.1 ± 0.75	342.7 ± 0.3	1050.1 ± 0.15	2.8	0.99	0.004	22.7
**5b**	108.3 ± 0.8	182.1 ± 0.42	488.4 ± 0.24	1.6	0.93	0.001	42.8
**6**	42.3 ± 0.2	78.0 ± 0.32	252.0 ± 0.36	1.5	0.99	0.006	100
**7**	77.2 ± 0.1	183.5 ± 0.4	950.2 ± 0.6	5.4	0.99	0.009	42.5
**8**	101.9 ± 0.95	191.6 ± 0.65	635.8 ± 0.29	1.4	0.92	0.001	40.7
**9**	88.9 ± 0.7	175.0 ± 0.25	633.7 ± 0.24	4.6	0.95	0.001	44.5
**10**	88.4 ± 0.5	139.9 ± 0.23	221.2 ± 0.61	4.1	0.95	0.002	55.7
**11b**	179.4 ± 0.6	308.8 ± 0.26	866.8 ± 0.68	3.6	0.87	0	25.2
**12**	112.1 ± 0.8	186.9 ± 0.34	649.9 ± 0.69	2.2	0.95	0	41.7
**13**	200.5 ± 0.65	386.1 ± 0.24	1341.4 ± 0.45	0.7	0.99	0.008	20.2
**14**	246.4 ± 0.1	357.8 ± 0.47	727.1 ± 0.25	1.8	0.97	0.002	21.7
**16**	80.2 ± 0.12	127.5 ± 0.36	307.2 ± 0.69	4.5	0.98	0.002	61.1
**Chlorpyrifos**	247.3 ± 0.31	588.3 ± 0.28	3052.4 ± 0.95	1.1	0.99	0.007	13.2

(LC (mg/L)) Lethal Concentration/ milligram per Litre.

(χ^2^cal./tab_._) Chi-square value calculated/tabulated.

(r^2^) Correlation coefficient.

Compound **6** exhibited the highest larvicidal activity (LC_50_ = 78.0 ± 0.32 mg/L), followed by compounds **16** (LC_50_ = 127.5 ± 0.36 mg/L) and **10** (LC_50_ = 139.9 ± 0.23 mg/L). Compounds **9**, **5b**, **7**, **12**, and **8** demonstrated moderate and convergent LC_50_ values, showing slightly lower potency compared to compound **6**. Compounds **11b**, **1a**, **5a**, **14**, **1b**, **3**, and **13** displayed convergent LC_50_ values, indicating the lowest larvicidal activity among the tested compounds. Nevertheless, all compounds outperformed Chlorpyrifos in terms of LC_50_ values (Table 1).

The toxicity index (TI), a key indicator of insecticidal potency, varied among the compounds. Compound **6** showed the highest TI value (100), while compound **13** exhibited the lowest (20.2). Chlorpyrifos had the lowest TI value (13.2) under the same conditions. The goodness of fit test confirmed the validity of the LDP line for all tested compounds, indicating the reliability of the results (Table 1). Finally, in control experiments, the larvae were exposed to the same concentrations (100 to 1000 mg/mL) of DMF solvent in water. Using the Abbott formula, which was employed to account for control mortality, the toxicity of DMF in water was under 5% which indicates low activity.

#### Molecular docking assessment

**Figure 3:**
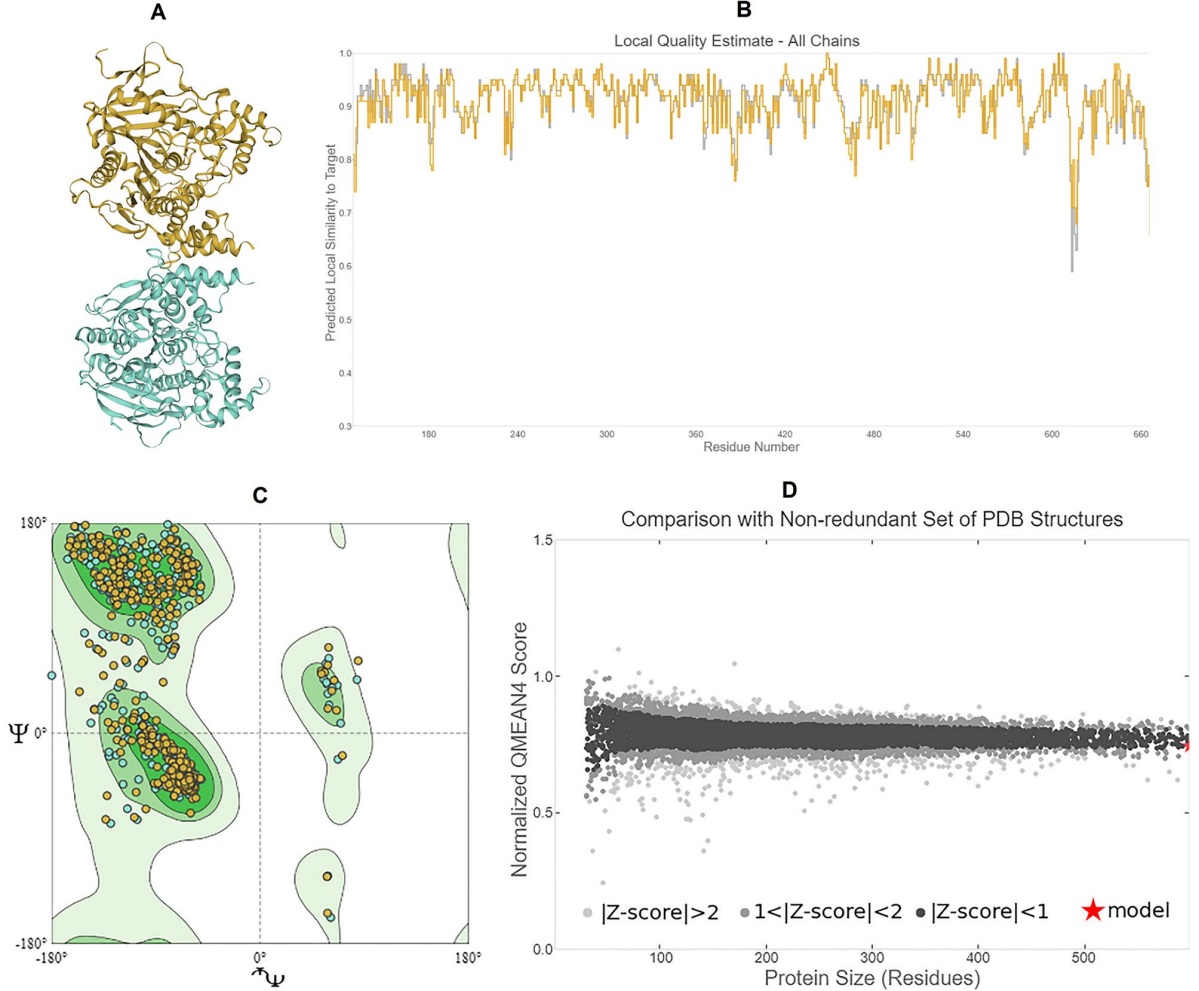
Quality estimate parameters for modeled *C. pipiens* acetylcholine esterase (AChE) protein (**a**) Modelled AChE 3D structure (**b**) Local model quality estimate (**c**) Ramachandran plot (**d**) Comparison with a non-redundant set of PDB structures.

Molecular docking studies were conducted to investigate the potential mode of action for the tested compounds on different neural receptors, and the resulting data was largely promising in identifying the potential neural targets that may lead to insect death. To study the binding interactions of the synthesized potential insecticides with mosquito neuroreceptors, three-dimensional (3D) structures of *C. pipiens* acetylcholinesterase (AChE), nicotinic acetylcholine receptor (nAChRs), and voltage-gated sodium channel binding protein alpha subunit (VGSC α subunit) were modeled using protein homology modeling, as they were not available in the Protein Data Bank (PDB). The AChE model was based on the crystal structure of *Anopheles gambiae* acetylcholinesterase in complex with PMSF (SMTL ID: 5ydj.1), while AlphaFold DB models of Q9W3G6.1.A Nicotinic acetylcholine receptor alpha3 and SCNA_DROME (gene: para) from *Drosophila melanogaster* were used for nAChRs and VGSC α subunit, respectively. These templates offered high query coverage (99%, 99%, and 98%) and good sequence identity (83.88%, 70.85%, and 87.04%) with the target *C. pipiens* proteins^28,48^. The quality of the AChE model was assessed using various parameters, including GMQE score (0.84), QMEAN Z-score (-0.53), and Ramachandran plot analysis (94.50% favored residues and 0.47% outliers), indicating good structural reliability and stability (Fig. 3)^28,48^.

**Figure 4:**
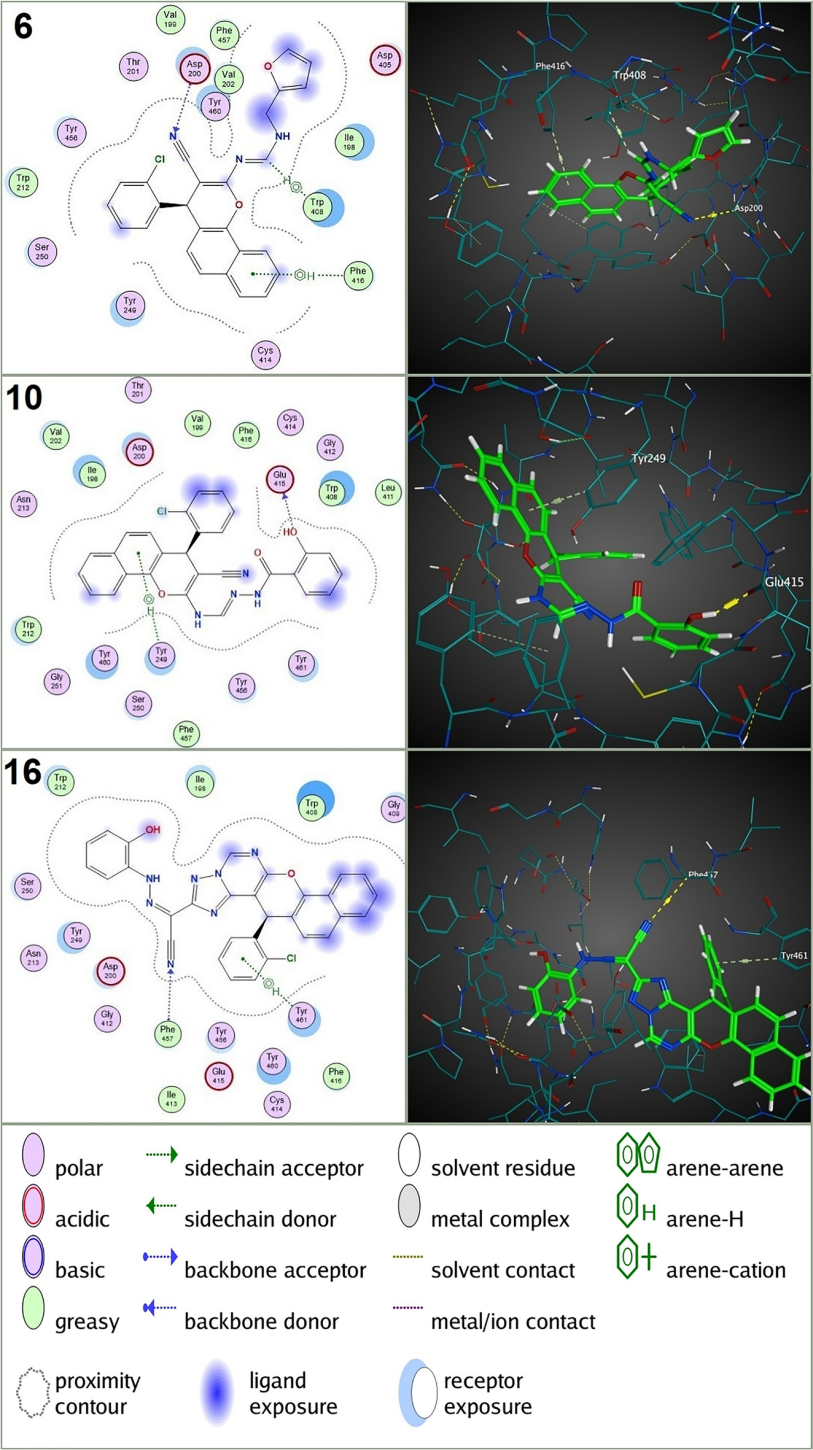
2D&3D molecular interactions of the compounds **6**, **10**, and **16** with AChE of *C. pipiens*. These **3** compounds showed the highest toxicity against *C.pipiens* larvae.

**Figure 5:**
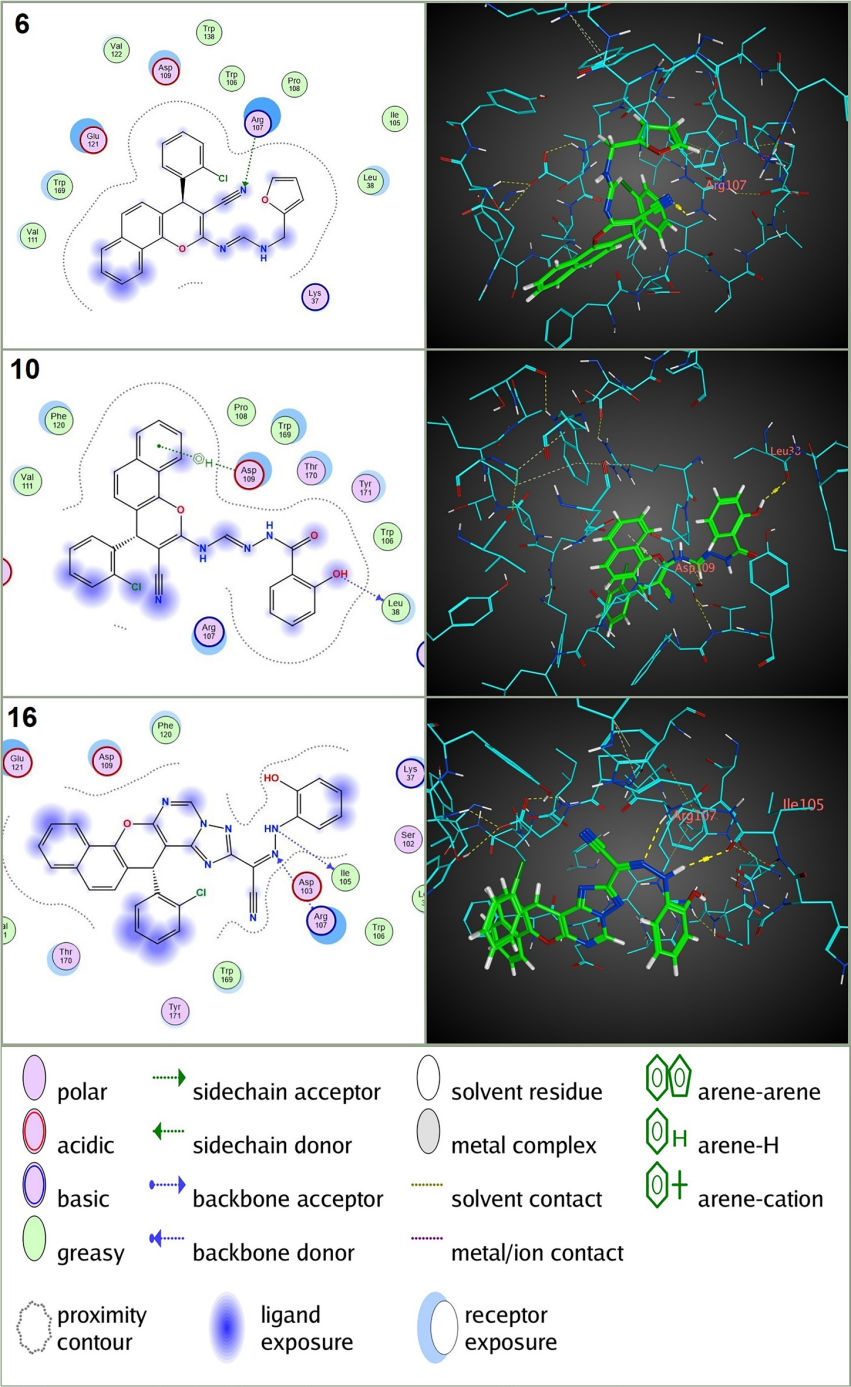
2D&3D molecular interactions of the compounds **6**, **10**, and **16** with nAChRs of *C. pipiens*. These **3** compounds showed the highest toxicity against *C.pipiens* larvae.

**Figure 6:**
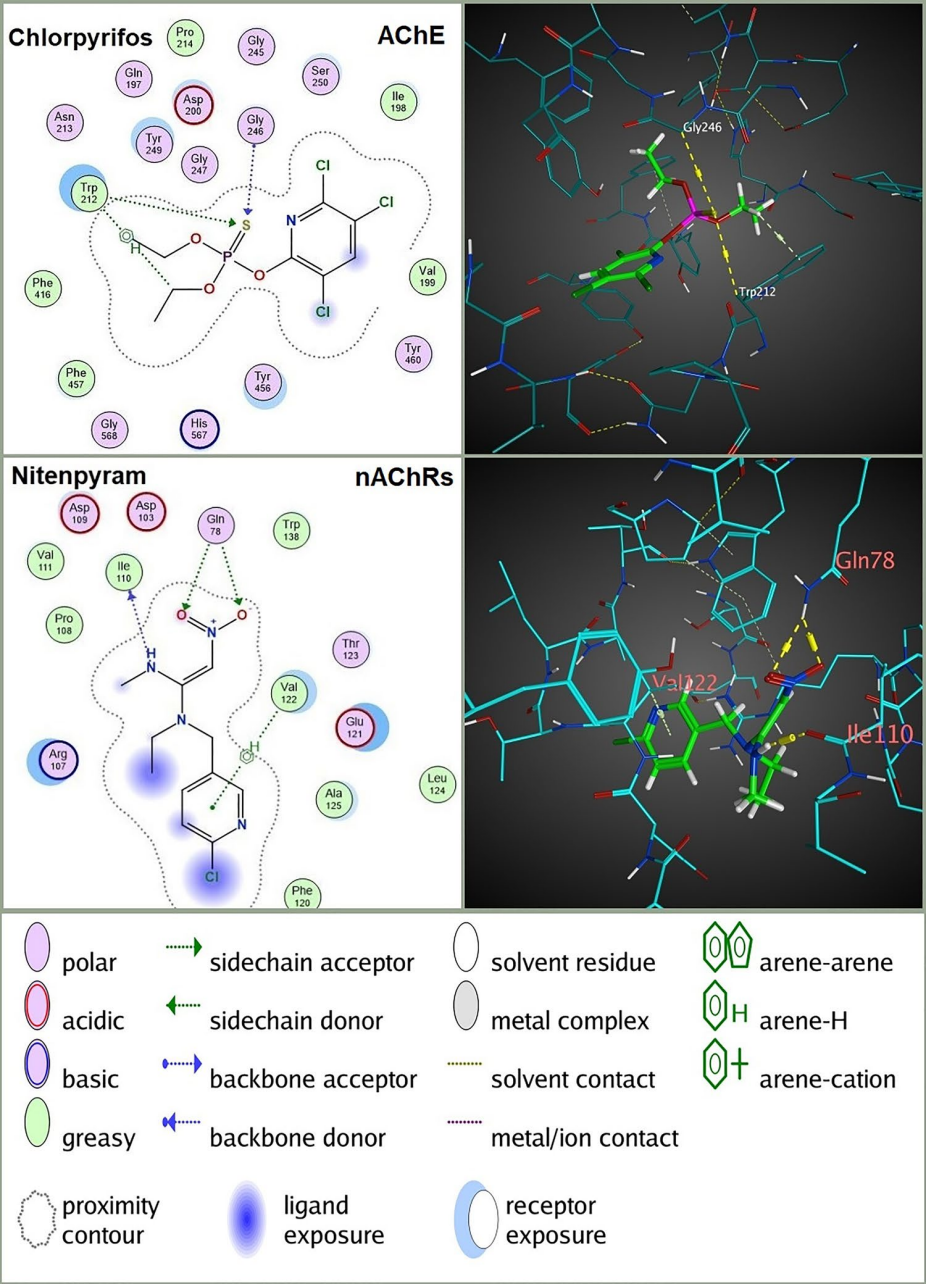
D&3D molecular interactions of the conventional acetylcholine esterase inhibitor, **Chlorpyrifos** with AChE of *C. pipiens*. and The conventional neonicotinoid, **Nitenpyram** with nAChRs of *C. pipiens*.

The molecular docking results revealed that all tested compounds exhibited favorable binding affinities (negative S scores) to the three target receptors (AChE, nAChRs, and VGSC α subunit), indicating potential interactions and supporting the observed larvicidal activity and neurotoxic symptoms in *C. pipiens* larvae. Notably, the most potent compounds (**6**, **10**, and **16**), which exhibited the lowest lethal concentrations (LC_50_) in larvicidal assays, consistently demonstrated the strongest binding affinities to both AChE and nAChRs. For instance, compound **6** displayed S scores of − 8.11 kcal/mol for AChE and − 6.27 kcal/mol for nAChRs (Table 2), forming an H-acceptor interaction with ASP 200 (B), H-pi interactions with TRP 408 (B), and pi-H interactions with PHE 416 (B) in the AChE active site (Fig. 4), and an H-acceptor interaction with ARG 107 (A) in the nAChRs active site (Fig. 5). Compound **10** displayed S scores of − 8.33 kcal/mol for AChE and − 6.74 kcal/mol for nAChRs (Table 2), forming an H-donor interaction with GLU 415 (B) and pi-H interactions with TYR 249 (B) in the AChE active site (Fig. 4), and an H-donor interaction with LEU 38 (A) and pi-H interactions with ASP 109 (A) in the nAChRs active site (Fig. 5). Compound **16** displayed S scores of − 8.28 kcal/mol for AChE and − 6.79 kcal/mol for nAChRs (Table 2), forming H-acceptor interactions with PHE 457 (B) and pi-H interactions with TYR 461 (B) in the AChE active site (Fig. 4), and H-donor and H-acceptor interactions with ILE 105 (A) and ARG 107 (A), respectively, in the nAChRs active site (Fig. 5). These values were significantly lower (more negative) than those of the reference compounds chlorpyrifos (S = − 6.89 kcal/mol for AChE, forming H-acceptor interactions with TRP 212 (B) and GLY 246 (B), and H-pi interactions with TRP 212 (B)) and nitenpyram (S = − 6.04 kcal/mol for nAChRs, forming H-donor and H-acceptor interactions with ILE 110 (A) and GLN 78 (A), respectively, and pi-H interactions with VAL 122 (A)) (Table 2 & Fig. 6). In addition to the neurotoxic effects observed in the biological assay, the in silico docking analysis revealed a stronger binding affinity of the novel compounds to AChE and nAChRs compared to chlorpyrifos and nitenpyram. This was evidenced by the formation of more hydrogen bonds with the active site gorge of these receptors and is consistent with their higher docking scores relative to the conventional insecticides. These findings support the hypothesis that the insecticidal activity of the novel compounds is primarily mediated through interactions with AChE, although nAChRs could also contribute to their neurotoxic effects. Furthermore, the analysis of connection types, residues connected, and the number of bonds predicted to be formed in all tested compounds compared to Chlorpyrifos and Nitenpyram further strengthens the hypothesis that these targets are involved in the mode of action of the novel compounds (S File 1).

**Table 2:** In silico docking analysis of binding affinity and pose fitness between novel annulated Benzo[h]chromenes and *C. pipiens* neural targets (AChE, nAChRs, and VGSC α subunit).

Compd	AChE		nAChRs		VGSC α subunit	
	*S	**RMSD Refine	S	RMSD Refine	S	RMSD Refine
**1a**	− 6.73	1.85	− 5.55	1.61	− 5.99	0.98
**1b**	− 6.63	1.11	− 5.65	0.83	− 5.30	1.34
**3**	− 6.86	1.94	− 5.99	1.14	− 6.04	1.69
**5a**	− 6.64	1.76	− 5.91	1.51	− 5.83	1.90
**5b**	− 7.15	1.08	− 6.19	1.56	− 5.66	0.85
**6**	− 8.11	1.31	− 6.27	1.20	− 6.42	1.81
**7**	− 7.40	1.01	− 6.67	1.35	− 6.18	1.73
**8**	− 6.61	1.41	− 5.48	1.19	− 5.49	1.32
**9**	− 7.25	1.32	− 6.44	1.06	− 6.09	1.14
**10**	− 8.33	1.61	− 6.74	1.34	− 6.28	1.62
**11b**	− 8.01	1.89	− 7.10	1.52	− 6.74	1.90
**12**	− 7.90	1.12	− 5.88	1.39	− 6.24	1.55
**13**	− 7.88	1.66	− 6.04	0.72	− 6.20	1.64
**14**	− 7.60	1.80	− 6.40	1.72	− 6.74	0.77
**16**	− 8.28	1.50	− 6.79	1.64	− 6.56	1.00
Reference insecticide	Chlorpyrifos		Nitenpyram		Indoxacarb	
	− 6.89	1.27	− 6.04	1.78	− 6.61	1.55

*S: Docking Score, kcal/mol.

**RMSD: Root mean square deviation, Å.

Interestingly, compounds with higher LC_50_ values generally exhibited weaker binding affinities to both AChE and nAChRs compared to compounds **6**, **10**, and **16**. This observation further reinforces the hypothesis that AChE and nAChRs are the primary targets for these compounds, with binding affinity playing a significant role in their insecticidal efficacy and the severity of neurotoxic symptoms induced in *C. pipiens* larvae. However, the relationship between binding affinity and insecticidal activity was not always straightforward. Some compounds, such as **11b** and **13**, showed a relatively strong binding affinity to AChE (-8.01 and -7.88 kcal/mol, respectively) despite having moderate LC_50_ values. This suggests that factors other than binding affinity, such as pharmacokinetics or the specific interactions within the binding pocket, may also influence the insecticidal activity and the resulting neurotoxic effects of these compounds. Furthermore, compounds **11b** and **14** exhibited good binding affinity to VGSC α subunit (S = − 6.74 kcal/mol), comparable to the reference compound indoxacarb (S = − 6.61 kcal/mol). Compound **11b** formed H-donor and ionic interactions with GLU 1405 (A) and LYS 1104 (A), respectively, while compound **14** formed H-donor and H-acceptor interactions with GLU 161 (A) and GLY 1109 (A), respectively. Indoxacarb formed an H-donor bond with GLU 1405 (A). This suggests that VGSC α subunit may also be a potential secondary target for these compounds.

Overall, the molecular docking results provide valuable insights into the potential mode of action of these novel benzochromene derivatives and their observed neurotoxic effects on *C. pipiens* larvae. The strong correlation between insecticidal activity, neurotoxicity, and binding affinity to AChE and nAChRs suggests that these receptors are the primary targets for most of these compounds, with AChE likely being the more important target due to the stronger correlation between binding affinity and insecticidal activity. However, the results also indicate that VGSC α subunit may be a potential secondary target for some compounds and that factors beyond binding affinity can modulate insecticidal activity and neurotoxicity. It is important to note that this is a theoretical explanation based on docking studies, and further studies, including in vitro enzyme inhibition assays and in vivo assays, are needed to confirm these hypotheses and to elucidate the precise mechanisms by which these compounds interact with the receptors and exert their insecticidal and neurotoxic effects.

### Structure–activity relationship (SAR) study

The larvicidal efficacy of synthesized benzo[h]chromene derivatives against Culex pipiens larvae is significantly influenced by the interplay between structural modifications and their resulting interactions with multiple neural targets. Specifically, the nature of substituents on the benzo[h]chromene scaffold and the type of fused heterocyclic ring play pivotal roles.The presence of various functional groups, including halogens, aromatic rings, sulfur, and nitrogen-containing moieties, not only modulates interactions with these neural targets but also impacts other toxicological aspects. These include absorption, lipophilicity, bioavailability, and the potential for metabolic resistance within the insect.^49,50^ These functional groups can engage in specific interactions with amino acid residues within target receptors, utilizing mechanisms such as hydrogen bonding, pi-pi stacking, or ionic interactions. Such interactions can lead to neurotoxic effects, culminating in the death of mosquito larvae. These diverse factors collectively contribute to the observed variations in insecticidal activity among the tested compounds. The interaction with neural targets, particularly acetylcholinesterase (AChE), emerges as a key factor in the toxicity of these derivatives. The higher binding affinity to AChE compared to other neural receptors suggests that AChE inhibition is a primary mechanism of action. The specific orientation and electronic nature of functional groups, whether electron-withdrawing or donating, can influence the strength and specificity of these interactions. Furthermore, structural modifications can affect steric hindrance or electron density distribution, thereby altering the interaction of the tested compounds with the target receptor and, consequently, its toxicity. This can lead to either increased or decreased insecticidal activity, as observed across all tested compounds^51–53^.

The precursor compounds, 2-amino-4-aryl-4H-benzo[h]chromene-3-carbonitriles (**1a**, **1b**), displayed comparatively lower larvicidal activity than most of their derivatives. This suggests that while the 2-amino and 3-cyano groups may contribute to insecticidal properties, their presence alone does not confer optimal potency against *C. pipiens* larvae. Molecular docking analysis corroborates this observation, revealing that **1a** and **1b** primarily interact with AChE through a single hydrogen bond between the cyano group (N37) and ASP200, along with pi-H interactions between the benzo[h]chromene scaffold and aromatic residues (TRP408 and PHE416) in the enzyme largest pocket. Notably, the docking scores for **1a** and **1b** are higher than those of the more potent compounds **6**, **10**, and **16**, indicating a potential correlation between lower AChE binding affinity and lower larvicidal efficacy noticed for **1a** and **1b**.

Acetylation and subsequent cyclization of **1b** yielded compound **3**, which showed a slight decrease in insecticidal activity compared to **1b**. This decrease can be attributed to the loss of the cyano-ASP200 hydrogen bond interaction, present in **1b**, and the formation of a new, potentially less favorable, hydrogen bond between CL 32 and ILE198 in the AChE active site in **3**. Additionally, **1b** formed a pi-H interaction with PHE416 that was not observed in **3**.

Condensation of enaminonitriles **1a** and **1b** with triethyl orthoformate yielded the corresponding ethyl formimidates **5a** and **5b**, respectively. Notably, **5b** exhibited a significant increase in larvicidal activity compared to its parent compound **1b**, while **5a** did not exhibit a significant increase in activity compared to **1a**. The docking scores (S) of **1b** and **5b** are − 6.63 and − 7.15 kcal/mol, respectively. This indicates that **5b** binds more strongly to AChE than **1b**. The increased binding affinity of **5b** could be due to the ethyl formimidate group being less bulky than the amino group in **1b**, reducing steric hindrance and allowing for a more favorable interaction with the AChE active site. Additionally, the ethyl formimidate group may form favorable interactions with other residues in the active site, further increasing the binding affinity of **5b**. These factors could contribute to the increased larvicidal activity of **5b** compared to **1b**.

Aminolysis of **5a** with heteroamines led to the replacement of the ethoxy group (–OCH_2_CH_3_) with a formamidine group, containing a nitrogen atom that can act as a hydrogen bond donor. This structural modification resulted in the formation of compounds **6** and **7**, which exhibited enhanced larvicidal activity compared to **5a**. The increase in potency can be attributed to the formation of a stronger hydrogen bond between the cyano group (CN) at the 2-position and ASP200 in the AChE active site. The docking scores of **6** and **7** (− 8.11 and − 7.40 kcal/mol, respectively) are lower (more negative) than those of **5a** (− 6.64 kcal/mol), indicating stronger binding affinity to AChE. The additional H-pi interaction observed between the furan ring of **6** and TRP408 may further enhance its binding affinity and inhibitory activity compared to **7**.

Further modifications of **5a** led to the synthesis of hydrazinolysis products (**8**, **9**, **10,11b**) and chromenotriazolopyrimidine derivatives (**12,13**), which exhibited moderate larvicidal activity. The moderate activity of these compounds suggests that while the modifications introduced new interactions with AChE, they may not have been as optimal as the interactions observed in the most potent compounds. For example, compound **8** formed a hydrogen bond with ILE198, similar to compound **3**, but lacked the additional interactions observed in compounds **6**, **10**, and **16**. Compound **9** formed a hydrogen bond with SER250, a residue not involved in the interactions of the most potent compounds, suggesting that this interaction may not be as crucial for larvicidal activity. Among the hydrazinolysis products, compound **10** stood out due to its ability to form a strong hydrogen bond with GLU415, a key residue in the AChE active site, contributing to its higher potency compared to other derivatives in this group. This interaction with GLU415 was unique to compound **10** and may explain its increased activity.

Compound **16** a hydrazonyl derivative of **13**, displayed the second-highest larvicidal activity, likely due to its ability to form multiple hydrogen bonds (with PHE457 and TYR461) and hydrophobic interactions with key residues in the AChE active site gorge. The presence of the hydrazonyl moiety in **16** may have introduced these additional interactions, contributing to its enhanced binding affinity and inhibitory activity compared to other triazolopyrimidine derivatives.

Interestingly, the triazolopyrimidine derivatives (**12**, **13**, and **14**) interacted with AChE through pi-H and H-pi interactions, which were not observed in the hydrazinolysis products. This suggests that the triazolopyrimidine ring system may be involved in different interactions with AChE compared to the hydrazinolysis products, potentially influencing their larvicidal activity. Additionally, the presence of a phenyl group in compound **12** may have contributed to its higher activity compared to **13** and **14** due to additional hydrophobic interactions. However, although compound 12 does not show a hydrogen bond formed with AChE its potent toxicity may be related to its interactions with nAChRs and VGSC α subunit since it forms ILE 105 (A) H-donor with nAChRs and GLU 160 (A) H-acceptor with VGSC α subunit.

While the cyano (CN) group plays a major role in the interaction of potent compounds with the ASP200 residue of acetylcholinesterase (AChE) in *C. pipiens* (S. File 1), the exact composition of the catalytic triad in this species remains to be elucidated. In most vertebrates, AChE inhibition typically involves interactions with the serine, histidine, and glutamic acid residues of the established catalytic triad^54^. However, due to potential species-specific variations in AChE structure, it is crucial to identify the corresponding catalytic triad residues in *C. pipiens* to fully understand the mechanism of AChE inhibition by these compounds. This will provide valuable insights into the selectivity and specificity of these larvicides, contributing to the development of more targeted and effective mosquito control strategies.

While molecular docking offers valuable insights into structure–activity relationships (SAR), it is inherently limited by its static nature and inability to fully capture the dynamic interplay between ligands and proteins, as well as the influence of solvent effects. Consequently, docking scores may not always accurately reflect experimental binding affinities^55^. To address these limitations and enhance our understanding of SAR, incorporating molecular dynamics (MD) simulations and free energy calculations can provide a more comprehensive and dynamic perspective on compound-target interactions. MD simulations can reveal conformational changes, induced fit effects, and the role of water molecules in ligand binding, while free energy calculations can offer more accurate estimates of binding affinities^56^.

In addition, further in vitro investigations into the precise mechanisms of interaction between benzo[h]chromene derivatives and their target receptors, including AChE, as well as the resulting insecticidal and neurotoxic effects, remain a crucial avenue for future research. This will not only deepen our understanding of the molecular basis of their larvicidal activity but also aid in the rational design of more effective and selective compounds for mosquito control.

## Experimental

### Chemistry

All melting points were measured on a Griffin and George melting-point apparatus (Griffin & Georgy Ltd., Wembley, Middlesex, UK) and are uncorrected. IR spectra were recorded on the Pye Unicam SP1200 spectrophotometer (Pye Unicam Ltd., Cambridge, UK) by using the KBr wafer technique. ^1^H-NMR spectra were determined on a Varian Gemini 300 MHz on Bruker Avance III using tetramethylsilane as an internal standard (chemical shifts in δ scale), while ^13^C NMR spectra were run at 75 MHz. EI-MS was measured on a Shimadzu GC–MS (Columbia, MD) operating at 70 eV. Elemental analyses were carried out at the Microanalytical Unit, Faculty of Science, Ain Shams University, using a Perkin-Elmer 2400 CHN elemental analyzer (Waltham, MA), and satisfactory analytical data (± 0.4) were obtained for all compounds. The homogeneity of the synthesized compounds was controlled by thin layer chromatography (TLC), using aluminum sheet silica gel F_254_ (Merck).

### 2-Amino-4-(2-chlorophenyl)-4H-benzo[h]chromene-3-carbonitrile 1a and 2-amino-4-(4-chlorophenyl)-4H-benzo[h]chromene-3-carbonitrile 1b

Typical reaction procedure for the synthesis of naphthopyrane (multi-component step): 1-naphthol (5 g, 3.4 mmol) was mixed with malononitrile (1.88 mL, 3.4 mmol), *o*-chlorobenzaldehyde and/or *p*-chlorobenzaldehyde (3.8 mL, 3.4 mmol) in (50 mL) absolute ethanol containing piperidine (2 mL). The mixture was allowed to reflux for about 7 h. The obtained solid was filtered off and washed with ethanol then recrystallized from dioxane to give **1a** and **1b**, respectively.

**1a:** brown crystals, yield: 91%, m.p. 239–240 ^0^C (Lit. m.p. 238–240)^39^.

**1b:** pale yellow crystals, yield: 86%, m.p. 234–235 ^0^C (Lit. m.p. 230–232)^39,40^.

### 7-(2-Chlorophenyl)-10-methyl-7,9-dihydro-8H-benzo-[7,8]chromeno[2,3-d]pyrimidin-8-one 2

A solution of enaminonitrile **1a** (1 g, 3 mmol) in acetic anhydride (20 mL) was heated under reflux for 12 h. The formed solid while reflux was filtered off, dried, and then crystallized from dioxane to give **2** as buff crystals; yield: 55%, m.p. > 300 oC. IR (KBr, cm-^−1^): 3156 (NH), 1655 (C=O). ^1^H-NMR (300 MHz, DMSO-*d6*) *δ* (ppm): 2.34 (s, 3H, CH_3_), 5.70 (s, 1H, C_4_-pyran), 7.17–7.65 (m, 8H, Ar–H), 7.86 (d, 1H, Ar–H, *J* = 8.1 Hz), 8.23 (d, 1H, Ar–H, *J* = 8.1 Hz), 12.50 (s, 1H, NH, exchangeable with D_2_O). ^13^C-NMR (DMSO-*d*_*6*_) δ (ppm): 20.97, 35.85, 98.81, 117.78, 120.75, 123.07, 124.41, 125.63, 126.87, 126.93, 127.65, 127.71, 128.29, 129.51, 130.96, 131.88, 132.81, 142.66, 143.40, 159.01, 161.55, 162.18. MS (m/z, %): 376 (M^+.^ + 2, 29.7%), 374 (M^+.^, 25.71%), 217 (100%), 110 (36.12%), 72 (83.97%) Anal. Calc. for C_22_H_15_ClN_2_O_2_ (374.84): C, 70.50; H, 4.03; Cl, 9.46; N, 7.47. Found: C, 70.58; H, 4.13; Cl, 9.26; N, 7.56.

### 10-(Chloromethyl)-7-(4-chlorophenyl)-7,9-dihydro-8H-benzo[7,8]chromeno[2,3-d]pyrimidin-8-one 3

A solution of enaminonitrile **1b** (1 g, 3 mmol) and chloroacetyl chloride (0.23 mL, 3 mmol) in dioxane (15 mL) was heated under reflux for 6 h. The reaction mixture was cooled and then poured into ice-cold water. The obtained solid was filtrated off and crystallized from benzene to give **3** as pale-yellow crystals; yield: 74%, m.p: 294–295 oC. IR (KBr, cm^-1^): 3320 (br, NH), 2959, 2851(CH alph.), 1657 (C=O), ^1^H-NMR (300 MHz, DMSO-*d6*) *δ* (ppm): 4.54 (s, 2H, CH_2_), 5.32 (s, 1H, C_4_-pyran), 7.25–7.67 (m, 8H, Ar–H), 7.90 (d, 1H, Ar–H, *J* = 7.8 Hz), 8.26 (d, 1H, Ar–H, *J* = 8.1 Hz), 13.02 (br s, 1H, NH, exchangeable with D_2_O). ^13^C-NMR (300 MHz, DMSO-*d6*) *δ* (ppm): 38.02, 42.13, 101.69, 118.23, 120.68, 123.05, 124.65, 126.51, 126.90, 126.98, 127.76, 128.38, 128.80, 129.42, 129.89, 131.28, 132.83, 143.54, 143.97, 156.61, 161.05, 162.09. MS (m/z, %): 411 (M^+.^ + 2, 15.44%), 409 (M^+.^, 18.22%), 36 (35.09%), 295 (100%), 161 (74.94%), 149 (48.47%), 106 (73.54%), 75 (43.53%). Anal. Calc. for C_22_H_14_Cl_2_N_2_O_2_ (409.27): C, 64.56; H, 3.45; Cl, 17.32; N, 6.84. Found: C, 64.65; H, 3.49; Cl, 17.22; N, 6.90.

### 7-(2-Chlorophenyl)-10-methyl-7H,8H-benzo[7,8]chromeno[2,3-d] [1,3]oxazin-8-one 4

A solution of enaminonitrile **1a** (1 g, 3 mmol) in acetyl chloride (20 mL) was refluxed for 18 h on a water bath. The excess solvent was evaporated and the solid formed was crystallized from benzene to give **4** as brown crystals; yield: 53%, m.p. 225–228 ^0^C. IR (KBr, cm^−1^): 1732 (C = O), 1656 (C=N). ^1^H-NMR (300 MHz, DMSO-*d6*) *δ* (ppm): 2.59 (s, 3H, CH_3_), 5.92 (s, 1H, C_4_-pyran), 7.18–7.89 (m, 9H, Ar–H), 8.26 (d, 1H, Ar–H, *J* = 8.4 Hz). ^13^C-NMR (DMSO-*d*_*6*_) δ (ppm): 24.97, 38.65, 111.38, 117.11, 120.68, 122.85, 124.90, 125.25, 127.25, 127.79, 128.10, 128.30, 129.17, 129.88, 131.89, 132.97, 140.97, 160.26, 167.08. MS (m/z, %): 377 (M^+.^ + 2, 7.87%), 375 (M^+.^, 12.56%), 261(100%), 218 (56.87), 179 (47.61%), 143 (90.03%), 133 (50.17%). Anal. Calc. for C_22_H_14_ClN_2_O_3_ (375.81): C, 70.31; H, 3.75; Cl, 9.43; N, 3.73. Found: C, 70.23; H, 3.91; Cl, 9.54; N, 3.81.

### Ethyl N-[4-(2-chlorophenyl)-3-cyano-4H-benzo[h]chromen-2-yl] formimidate 5a and Ethyl N-[4-(4-chlorophenyl)-3-cyano-4H-benzo[h]chromen-2-yl] formimidate 5b

A solution of enaminonitrile **1a** or **1b** (1 g, 3 mmol) in triethylorthoformate (20 mL) was heated under reflux for 8 h. The reaction solution was evaporated till dryness. The obtained solid was filtrated off and crystallized from ethanol to give **5a** and **5b**, respectively.

**5a**: yellow crystals; yield: 79%, m.p. 177–179 ^0^C. IR (KBr, cm^-1^): 2207 (C≡N), 1656 (C=N). ^1^H-NMR (300 MHz, DMSO-*d6*) *δ* (ppm): 1.21 (*Syn*), 1.36 (*Anti*) (2t, 3H, CH_2_*CH*_*3*_, *J* = 6.9 Hz), 4.13 (*Syn*), 4.38 (*Anti*) (2q, 2H, *CH*_*2*_CH_3_, *J* = 6.9 Hz), 5.63 (s, 1H, C_4_-pyran), 6.97 (d, 1H, Ar–H, *J* = 8.7 Hz), 7.30–7.65 (m, 7H, Ar–H), 7.88 (d, 1H, Ar–H, *J* = 9 Hz), 8.36 (d, 1H, Ar–H, *J* = 7.2 Hz), 8.18 (*Syn*), 8.94 (*Anti*) (2 s, 1H, N = CH). ^13^C-NMR (300 MHz, DMSO-*d6*) *δ* (ppm): 13.92, 18.57, 54.01, 78.75, 115.37, 117.63, 121.32, 122.83, 124.93, 125.24, 127.04, 127.15, 127.65, 128.15, 129.50, 130.01, 131.82, 132.28, 133.01, 140.50, 143.12, 157.72, 161.92. MS (m/z, %): 390 (M^+.^ + 2, 16.04%), 388 (M^+.^, 23.09%), 375 (72%), 349 (52.68%), 254 (48.50%), 247 (100%), 245 (61.71%), 232 (63.75%), 204 (92.47%), 161 (43.96%), 134 (30.74%) Anal. Calc. for C_23_H_17_ClN_2_O_2_ (388.85): C, 71.04; H, 4.41; Cl, 9.12; N, 7.20. Found: C, 71.20; H, 4.50; Cl, 9.26; N, 7.11.

**5b**: pale brown crystals; yield: 73%, m.p: 170–171 ^0^C. IR (KBr, cm^−1^): 2205 (C≡N), 1654 (C=N). ^1^H-NMR (300 MHz, DMSO-*d6*) *δ* (ppm): 1.36 (t, 3H, CH_2_*CH*_*3*_*,* *J* = 6.9 Hz), 4.42 (q, 2H, *CH*_*2*_CH_3_, *J* = 6.9 Hz), 5.22 (s, 1H, C_4_-pyran), 7.07 (d, 1H, Ar–H, *J* = 8.4 Hz), 7.34–7.67 (m, 7H, Ar–H), 7.89 (d, 1H, Ar–H, *J* = 8.4 Hz), 8.36 (d, 1H, Ar–H, *J* = 7.5 Hz), 8.94 (s, 1H, N=CH). ^13^C-NMR (300 MHz, DMSO-*d6*) *δ* (ppm): 13.92, 41.23, 63.99, 79.80, 116.06, 117.89, 121.31, 122.92, 124.84, 125.94, 127.05, 127.13, 127.66, 128.92, 130.13, 132.17, 132.95, 142.86, 142.99, 157.43, 162.01. MS (m/z, %): 390 (M^+.^ + 2, 7.51%), 388 (M^+.^, 10.95%), 380 (53.96%), 328 (62.27%), 286 (62.44%), 273 (61.03%), 147 (100%). Anal. Calc. for C_23_H_17_ClN_2_O_2_ (388.85): C, 71.04; H, 4.41; Cl, 9.12; N, 7.20. Found: C, 71.13; H, 4.21; Cl, 9.18; N, 7.04.

### N'-{4-(2-Chlorophenyl)-3-cyano-4H-benzo[h]chromen-2-yl}-N-(furan-2-ylmethyl) formimidamide 6

A solution of **5a** (1 g, 2.5 mmol) in dioxane (15 mL) and 2-furanylmethanamine (0.22 mL, 2.5 mmol) was refluxed for 12 h. The reaction mixture was cooled and then poured onto ice-cold water. The obtained solid was filtrated off and crystallized from benzene to give **6** as yellow crystals, yield: 82%, m.p. 138–139 ^0^C. IR (KBr, cm^-1^): 3432 (NH), 2191 (C≡N), 1657 (C=N). ^1^H-NMR (300 MHz, DMSO-*d6*) *δ* (ppm): 5.40 (s, 1H, C_4_-pyran), 5.83 (s, 2H, CH_2_), 6.13 (s, 1H, NH exchangeable with D_2_O), 6.29 (d, 1H, furan ring), 6.97–8.31 (m, 12H, 10Ar-H + 2H furan ring), 8.33 (s, 1H, N=CH). ^13^C-NMR (300 MHz, DMSO-*d6*) *δ* (ppm): 36.69, 37.93, 95.36, 106.08, 110.31, 116.7, 120.73, 122.64, 123.02, 124.01, 125.4, 126.73, 126.88, 126.94, 127.23, 127.71, 127.92, 128.17, 128.84, 129.29, 129.78, 130.14, 130.37, 131.19, 131.76, 131.95,132.81, 132.93, 140.09, 142.06, 142.18, 143.84, 152, 156.69, 160.36, 160.48, 162.08 . MS (m/z, %): 441 (M^+.^ + 2, 24.71%), 439 (M^+.^, 18.37%), 330 (100%), 234 (60.20%), 295 (90.28%), 165(32.71%), 122 (49.68%), 65 (34.37%) Anal. Calc. for C_26_H_18_ClN_3_O_2_ (439.90): C, 70.99; H, 4.12; Cl, 8.06; N, 9.55. Found: C, 70.85; H, 4.30; Cl, 8.26; N, 9.44.

### N'-{4-(2-Chlorophenyl)-3-cyano-4H-benzo[h]chromen-2-yl}-N-(pyridin-2-yl) formamidine 7

A solution of **5a** (1 g, 2.5 mmol) in dioxane (15 mL) and 2-aminopyridine (0.24 mL, 2.5 mmol) was refluxed for 24 h. The reaction mixture was cooled and then poured onto ice-cold water. The obtained solid was filtrated off and crystallized from ethanol to give **7** as pale-yellow crystals, yield: 65%, m.p. 222–224 ^0^C. IR (KBr, cm^−1^): 3397 (NH), 2191 (C≡N), 1644 (C=N). ^1^H-NMR (300 MHz, DMSO-*d6*) *δ* (ppm): 6.27 (s, 1H, C_4_-pyran), 7.00–7.91 (m, 11H, 8H Ar–H + 3H pyridine), 8.09–8.12 (m, 2H, _C6_-pyridine moiety + NH exchangeable with D_2_O), 8.26–8.33 (m, 2H, Ar–H), 8.57 (s, 1H, N = CH). ^13^C-NMR (300 MHz, DMSO-*d6*) *δ* (ppm): 36.39, 98.74, 113.66, 116.92, 118.70, 120.78, 122.97, 124.39, 125.56, 127.07, 127.78, 127.96, 128.29, 129.55, 130.39, 131.57, 131.79,132.97, 137.99, 140.41, 143.43, 148.00, 151.90, 156.59, 157.93, 162.85. MS (m/z, %): 439 (M^+.^ + 2, 1.54%), 437 (M^+.^, 10.89%), 404 (42.40), 394 (38.26%), 360 (30.35%), 254 (35.13%), 105 (100%), 94 (38.17%). Anal. Calc. for C_26_H_17_ClN_4_O (436.90): C, 71.48; H, 3.92; Cl, 8.11; N, 12.82. Found: C, 71.60; H, 3.82; Cl, 8.29; N, 12.88.

### 7-(2-Chlorophenyl)-8-imino-7H-benzo[7,8]chromeno[2,3-d]-pyrimidin-9(8H)-amine 8

A solution of **5a** (1 g, 2.5 mmol) in excess hydrazine hydrate (3 mL) was stirred at room temperature for 10 h. The formed solid was filtered off, dried, and crystallized from toluene to give **8** as pale brown crystals, yield: 70%, m.p: 202–205 ^0^C. IR (KBr, cm^−1^): 3336, 3319, 3284, 3180 (NH, NH_2_), 1652 (C=N). ^1^H-NMR (300 MHz, DMSO-*d6*) *δ* (ppm): 5.74 (s, 2H, NH_2_ exchangeable with D_2_O), 5.79 (s, 1H, C_4_-pyran), 6.80 (br.s, 1H, NH exchangeable with D_2_O), 7.18–7.67 (m, 8H, Ar–H), 7.88 (d, 1H, Ar–H, *J* = 8.1 Hz), 8.23 (s, H, Ar–H), 8.25 (s, 1H, pyrimidine-H). ^13^C-NMR (300 MHz, DMSO-*d6*) *δ* (ppm): 37.21, 97.38, 116.91, 120.77, 122.98, 124.39, 125.50, 126.91, 127.73, 127.97, 128.83, 129.78, 131.10, 131.98, 132.85, 141.42, 143.29, 151.22, 155.04, 156.74. MS (m/z, %): 376 (M^+.^ + 2, 27.86%), 374 (M^+.^, 28.40%), 353 (95.12%), 347 (82.94%), 342 (100%), 287 (47.04%), 207 (61.97%). Anal. Calc. for C_21_H_15_ClN_4_O (374.83): C, 67.29; H, 4.03; Cl, 9.46; N, 14.95. Found: C, 67.14; H, 4.23; Cl, 9.52; N, 15.07.

### 1-[{4-(2-Chlorophenyl)-3-cyano-4H-benzo[h]chromen-2-ylimino} methyl]thiosemicarbazide 9

A mixture of iminoether derivative **5a** (1 g, 2.5 mmol) and thiosemicarbazide (0.23 g, 2.5 mmol) in dioxane (20 mL) was heated under reflux for 15 h. The formed solid while reflux was filtered off, dried, and then crystallized from ethanol to give **9** as brown crystals, yield: 73%, m.p. 256–259 °C. IR (KBr, cm^−1^): 3467, 3308, 3155 (NH_2_, 2NH), 2191 (CN), 1645 (C = N), 1261 (C=S). ^1^H-NMR (300 MHz, DMSO-*d6*) *δ* (ppm): 5.76 (s, 1H, C_4_-pyran), 6.35 (s, 1H, NH exchangeable with D_2_O), 6.98–7.41 (m, 7H, 5Ar-H + NH_2_ exchangeable with D_2_O), 7.54–7.74 (m, 3H, 3Ar-H + NH exchangeable with D_2_O), 7.87 (d, 1H, Ar–H, *J* = 8.1 Hz), 8.23 (s, 1H, N = CH), 8.29 (d, 1H, Ar–H, *J* = 8.1 Hz). ^13^C-NMR (300 MHz, DMSO-*d6*) *δ* (ppm): 37.04, 94.48, 116.95, 120.85, 123.06, 123.94, 125.74, 126.85, 126.92, 127.71, 127.94, 129.23, 130.34, 131.76, 131.95, 132.92, 140.26, 143.95, 156.90, 162.41, 162.72. MS (m/z, %): 435 (M^+.^ + 2, 20.23%), 433 (M^+.^, 35.18%), 412 (63.51), 399 (54.78%), 383 (50.44%), 334 (66.04%), 320 (70.40%), 303 (88.43%), 242 (56.11%), 198 (63.63%), 191 (74.85%), 168 (74.94%), 164 (100%), 120 (48.24%), 118 (82.67%), 112 (89.93%), 110 (84.68%), 92 (56.39%), 87 (42.86%), 72 (54.82%). Anal. Calc. for C_22_H_16_ClN_5_OS (433.91): C, 60.90; H, 3.72; Cl, 8.17; N, 16.14; S, 7.39. Found: C, 60.98; H, 3.57; Cl, 8.07; N, 16.32; S, 7.31.

### N'-{[(4-(2-Chlorophenyl)-3-cyano-4H-benzo[h]chromen-2-ylimino]methyl}-2-hydroxybenzo hydrazide 10

A mixture of imnioether **5a** (1 g, 2.5 mmol) and salicylhydrazide (0.39 g, 2.5 mmol) in dioxane (15 mL) was heated under reflux for 20 h. The reaction mixture was cooled and then poured onto ice-cold water. The obtained solid was filtrated off then boiled with petroleum ether and then crystallized from benzene to give **10** as pale brown crystals, yield: 75%, m.p. 253–256 °C. IR (KBr, cm^-1^): br. 3184 (NH, OH), 2192 (C≡N), 1660 (C=O). ^1^H-NMR (DMSO-*d*_*6*_) (*δ* ppm): 6.27 (s, 2H, C_4_-pyran + NH exchangeable with D_2_O), 6.94–8.34 (m, 15H, 14Ar-H + NH exchangeable with D_2_O), 9.74 (s, 1H, N = CH), 10.92 (s, 1H, OH, exchangeable with D_2_O). ^13^C-NMR (300 MHz, DMSO-*d6*) *δ* (ppm): 37.13, 38.21, 54.85, 114.96, 116.61, 117.30, 119.30, 119.31,119.77, 120.22, 120.28, 120.78, 121.63, 122.68, 124.14, 125.49, 126.82, 126.97, 127.29, 128.03,128.86, 128.94, 129.29, 129.43, 129.56, 129.69, 129.86, 131.26, 132.03, 132.86, 133.12, 133.52, 134.15, 136.57, 140.91, 142.22, 157.67, 158.54, 160.41, 165.80. MS (m/z, %): 496 (M^+.^ + 2, 8.20%), 494 (M^+.^, 10.73%), 314 (19.84%), 289 (100%), 252 (48.07%), 194 (34.89%), 139 (41.68%). Anal. Calc. for C_28_H_19_ClN_4_O_3_ (494.94): C, 67.95; H, 3.87; Cl, 7.16; N, 11.32. Found: C, 67.75; H, 3.95; Cl, 7.20; N, 11.18.

### N-{7-(2-Chlorophenyl)-8-imino-7H-benzo[7,8]chromeno[2,3-d]-pyrimidin-9(8H)-yl}-4-methylbenzenesulfonamide 11a and N-[7-(4-Chlorophenyl)-8-imino-7H-benzo[7,8]chromeno[2,3-d]pyrimidin-9(8H)-yl]-4-methylbenzenesulfonamide 11b

A mixture of **5a** or **5b** (1 g, 2.5 mmol) and *p*-toluene sulphonohydrazide (0.48 g, 2.5 mmol) in dioxane (20 mL) was heated under reflux for 4 h. The formed solid while refluxing in each case was filtered off, dried, and crystallized from dioxane to give 11a and 11b, respectively.

11a: white crystals, yield: 60%, m.p. 279–280 °C. IR (KBr, cm^−1^): 3444, 3233 (2NH), 1648 (C=N). 1H-NMR (300 MHz, DMSO-d6) δ (ppm): 2.13 (s, 3H, CH_3_), 5.64 (s, 1H, C4-pyran), 6.85–6.87 (m, 2H, Ar–H), 7.00–7.03 (m, 1H, Ar–H), 7.20–7.48 (m, 6H, Ar–H), 7.59–7.61 (m, 3H, Ar–H), 7.76–7.86 (m, 3H, 1 Ar–H + 2NH exchangeable with D_2_O), 8.21–8.23 (m, 1H, Ar–H), 8.63 (s, 1H, pyrimidine-H). 13C-NMR (300 MHz, DMSO-d6) δ (ppm): 20.91, 38.38, 95.06, 114.79, 120.60, 122.54, 125.12, 125.43, 125.68, 127.35, 127.40, 127.86, 128.07, 128.89, 129.65, 131.34, 132.44, 132.69, 133.00, 137.34, 140.16, 140.48, 143.22, 153.15, 154.88, 159.38. MS (m/z, %): 531 (M + . + 2, 8.71%), 529 (M + ., 26.58%), 432 (50.86%), 312 (30.76%), 192 (69.75%), 82 (52.39%), 76 (100%), 65 (44.12%). Anal. Calc. for C28H21ClN4O3S (529.01): C, 63.57; H, 4.00; Cl, 6.70; N, 10.59; S, 6.06. Found: C, 63.37; H, 3.92; Cl, 6.84; N, 10.50; S, 6.18.

**11b**: white crystals, yield: 83%, m.p. 280–282 °C. IR (KBr, cm-1): 3443 (NH), 1651(C=N). ^1^H-NMR (300 MHz, DMSO-d6) δ (ppm): 2.20 (s, 3H, CH_3_), 5.41 (s, 1H, C_4_-pyran), 6.89 (d, 1H, Ar–H, *J* = 8.1 Hz), 7.21–7.35 (m, 8H, Ar–H), 7.57–7.70 (m, 3H, Ar–H), 7.91 (d, 1H, Ar–H, J = 7.8 Hz), 8.06 (br.s, 2H, 2NH exchangeable with D_2_O), 8.25 (d, 1H, Ar–H, *J* = 7.8 Hz), 8.62 (s, 1H, pyrimidine-H). ^13^C-NMR (300 MHz, DMSO-d6) δ (ppm): 20.76, 37.65, 96.66, 117.63, 120.46, 122.89, 125.46, 125.82, 127.17, 127.35, 127.82, 128.72, 128.80, 129.39, 131.98, 132.82, 140.13, 140.77, 141.25, 142.68, 152.99, 154.91, 158.90. MS (m/z, %): 531 (M + ^.^ + 2, 13.14%), 529 (M + ^.^, 57.35%), 510 (87.64%), 417 (100%), 376 (63.78%), 237 (56.82%), 131 (48.89%). Anal. Calc. for C_28_H_21_ClN_4_O_3_S (529.01): C, 63.57; H, 4.00; Cl, 6.70; N, 10.59; S, 6.06. Found: C, 63.44; H, 4.05; Cl, 6.79; N, 10.51; S, 6.20.

### 14-(4-Chlorophenyl)-2-phenyl-14H-benzo[7,8]chromeno[3,2-e][1,2,4]triazolo[1,5-c]pyrimidine 12

A mixture of imnioether **5b** (1 g, 2.5 mmol) and benzoylhydrazide (0.34 g, 2.5 mmol) in dioxane (15 mL) was heated under reflux for 15 h. The reaction mixture was cooled and then poured onto ice-cold water. The obtained solid was filtrated off and crystallized from dioxane to give **12** as pale-yellow crystals; yield: 65%, m.p: 296–297 °C. IR (KBr, cm^−1^): 3046 (CH-Ar), 1619 (C = N). ^1^H-NMR (300 MHz, DMSO-*d6*) *δ* (ppm): 5.98 (s, 1H, C4-pyran), 7.30–7.74 (m, 12H, Ar–H), 7.94 (d, 1H, Ar–H, *J* = 7.5 Hz), 8.16–8.17 (m, 1H, Ar–H), 8.36 (d, 1H, Ar–H, *J* = 8.1 Hz), 9.69 (s, 1H, pyrimidine-H). MS (m/z, %): 462 (M^+.^ + 2, 6.48%), 460 (M^+.^, 10.95%), 361 (58.62%), 321 (52.46%), 298 (86.61%), 40 (100%). Anal. Calc. for C_28_H_17_ClN_4_O (460.92): C, 72.96; H, 3.72; Cl, 7.69; N, 12.16. Found: C, 73.00; H, 3.82; Cl, 7.60; N, 12.21.

### 2-{14-(2-Chlorophenyl)-14H-benzo[7,8]chromeno[3,2-e][1,2,4]triazolo[1,5-c]pyrimidin-2-yl}acetonitrile 13

A mixture of imnioether derivative **5a** (1 g, 2.5 mmol) and cyanoacetohydrazide (0.25 g, 2.5 mmol) in dioxane (20 mL) was heated at reflux for 20 h. The reaction mixture was concentrated and allowed to cool. The residue was poured onto ice-cold water and the formed solid was filtered off, dried, and then crystallized from ethanol to give **13** as yellow crystals, yield: 62%, m.p. 156–159 °C. IR (KBr, cm^−1^): 3340 (NH), 2261, 2192 (C≡N), 1658 (C=N). ^1^H-NMR (300 MHz, DMSO-*d6*) *δ* (ppm): 3.58 (s, 2H, CH_2_CN), 4.39 (s, 1H, NH, exchangeable with D_2_O), 5.40 (s, 1H, C_4_-pyran), 6.27 (s, 1H, = CH), 6.98–8.23 (m, 10H, Ar–H), 9.73 (s, 1H, pyrimidine-H). MS (m/z, %): 425 (M^+.^ + 2, 15.85%), 423 (M^+.^, 4.17%), 413 (63.26%), 375 (41.30%), 303 (48.44%), 179 (70.39%), 75 (30.43%), 62 (100%). Anal. Calc. for C_24_H_14_ClN_5_O (423.86): C, 68.01; H, 3.33; Cl, 8.36; N, 16.52. Found: C, 68.09; H, 3.37; Cl, 8.26; N, 16.48.

### 2-{14-(2-Chlorophenyl)-14H-benzo[7,8]chromeno[3,2-e][1,2,4]-triazolo[1,5-c]pyrimidin-2-y}-3-(4-methoxyphenyl) acrylonitrile 14

A mixture of **13** (1 g, 2.3 mmol) and *p*-methoxybenzaldehyde (0.28 mL, 2.3 mmol) in dioxane (15 mL) containing drops of piperidine was refluxed for 9 h. The formed solid while heating was filtered off, dried, and then crystallized from dioxane/DMF (2:1) to give **14** as yellow crystals, yield: 52%, m.p. > 300 °C. IR (KBr, cm^−1^): 2221 (C≡N), 1626 (C=N). ^1^H-NMR (300 MHz, DMSO-*d6*) *δ* (ppm): 3.83 (s, 3H, OCH_3_), 6.24 (s, 1H, C_4_-pyran), 7.08–7.93 (m, 13H, Ar–H), 8.15 (s, 1H, C = CH), 8.30 (d, 1H, Ar–H, *J* = 7.8 Hz), 9.69 (s, 1H, pyrimidine-H). MS (m/z, %): 544 (M^+.^ + 2, 17.55%), 542 (M^+.^, 27.25%), 514 (40.57%), 497 (30.49%), 464 (34.08%), 441 (100%), 397 (31.98%), 252 (54.39%), 141 (40.41%), 134 (74.54%), 107 (65.92%). Anal. Calc. for C_32_H_20_ClN_5_O_2_ (542): C, 70.91; H, 3.72; Cl, 6.54; N, 12.92. Found: C, 70.77; H, 3.76; Cl, 6.45; N, 12.97.

### 3-[14-(2-Chlorophenyl)-14H-benzo[7,8]chromeno[3,2-e][1,2,4]triazolo[1,5-c]pyrimidin-2-yl]-2H-chromen-2-one 15

A mixture of **13** (1 g, 2.3 mmol) and salicylaldehyde (0.24 mL, 2.3 mmol) in dioxane (20 mL) containing drops of piperidine was refluxed for 5 h. The formed solid while heating was filtered off, dried, and then crystallized from dioxane/DMF (2:1) to give **15** as pale-yellow crystals, yield: 53%, m.p. > 300 °C. IR (KBr, cm-^−1^): 1747 (CO), 1644 (C=N). ^1^H-NMR (300 MHz, DMSO-*d6*) *δ* (ppm): 6.32 (s, 1H, C_4_-pyran), 7.01–8.34 (m, 14H, Ar–H), 8.31 (d, 1H, Ar–H), 8.63 (s, 1H, chromenone ring), 9.77 (s, 1H, pyrimidine-H). MS (m/z, %): 530 (M^+.^ + 2, 18.60%), 528 (M^+.^, 31.89%), 496 (40.38%), 386 (48.11%), 257 (37.17%), 96 (90.02%), 129 (46.64%), 59 (100%). Anal. Calc. for C_31_H_17_ClN_4_O_3_ (528.95): C, 70.39; H, 3.24; Cl, 6.70; N, 10.59. Found: C, 70.54; H, 3.31; Cl, 6.81; N, 10.55.

### 14-(2-Chlorophenyl)-N-(2-hydroxyphenyl)-14H-benzo[7,8]chromeno[3,2-e][1,2,4]triazolo[1,5-c]pyrimidine-2-carbohydrazonoyl cyanide 16

To *o*-amino phenol (1.1 g, 10 mmol), concentrated HCl (3 mL) was added and cooled to ~ 0–5 °C in an ice bath then cooled sodium nitrite solution (1.0 g in 10 mL of water) was added to the mixture dropwise during 10 min. The reaction mixture was then stirred for 10 min. A cold mixture of the acetonitrile derivative **13** (4.35 g, 10 mmol) and sodium acetate (4.10 g, 50 mmol) in ethanol (50 mL), was then added dropwise to the reaction mixture with stirring. The stirring was continued for 30 min, and the reaction mixture was left for 1 h at room temperature. The solid product obtained was collected by filtration and crystallized from ethanol to give **16** as reddish-brown crystals, yield: 61%, m.p: 182–183 oC. IR (KBr, cm^-1^): br. 3427 (OH, NH), 2258 (C≡N), 1625 (C = N). ^1^H- NMR (300 MHz, DMSO-*d6*) *δ* (ppm): 3.42 (br s, 2H, NH + OH, exchangeable with D_2_O), 6.11(s, 1H, C_4_-pyran), 7.14–7.64 (m, 12H, Ar–H), 7.83 (d, 1H, Ar–H, *J* = 7.8 Hz), 8.26 (d, 1H, Ar–H, *J* = 8.1 Hz), 9.67 (s, 1H, pyrimidine-H). ^13^C-NMR (300 MHz, DMSO-*d6*) *δ* (ppm): 37.18, 116.05, 116.38, 120.55, 122.86, 124.67, 125.20, 126.99, 127.63, 127.78, 128.95, 129.80, 131.66, 131.90, 132.85, 140.53, 141.02, 143.34, 152.81, 154.34, 160.90. MS (m/z, %): 545 (M^+.^ + 2, 14.13%), 543 (M^+.^, 18.93%), 422 (58.61%), 360 (73.98%), 335 (76.45%), 258 (50.47%), 264 (55.33%), 234 (100%), 215 (47.95%), 155 (68.82%), 122 (34.45%), 68 (42.01%), 41 (78.09%). Anal. Calc. for C_30_H_18_ClN_7_O_2_ (543.97): C, 66.24; H, 3.34; Cl, 6.52; N, 18.02. Found: C, 66.32; H, 3.46; Cl, 6.43; N, 18.16.

### Biological evaluation

#### Mosquito larval colony

The laboratory strain of *C. pipiens* was raised and maintained for roughly 24 generations in an insectary at the Entomology Department of the Faculty of Science, Ain Shams University, using the recommended techniques, under controlled conditions at 27 ± 2 °C and RH 75%, and photoperiod 12:12 light: dark hours^57^. TetraMin was administered to the newly hatched larvae. The pupae were placed within the (25 × 30 × 25) cm wooden cages before being moved. Adults received a 10% sucrose solution every day. The females were permitted to consume a meal of blood provided by a pigeon host^58^.

#### Biological assay

The World Health Organization's recommended larval bioassay test protocol was followed for conducting the experiments in a lab setting^59^. Twenty third-instar *C. pipiens* larvae were given a variety of insecticidal concentrations of the investigated chemicals ranging from 100 mg/L to 1000 mg/L. Before being diluted with water, the investigated substances were solubilized in dimethylformamide (DMF). Three replicates were used for each concentration, while DMF with water served as the control. The finding of larval mortality was noted after 12 h. Larvae were assumed to be dead if they did not react to touching^53^. A conventional insecticide “Chlorpyrifos” was tested also as a reference larvicidal compound under the same conditions used for testing our synthesized compounds.

#### Statistical analysis

The data on larval mortality were examined using the LDP line program's statistical capabilities. The lethal concentrations (LC_25_, LC_50_, LC_90_) were calculated using a 95% confidence interval (C.I.). Additionally, the Abbott formula was employed to account for control mortality, along with the Finney formula, the Chi-square test, and the goodness of fit test (r2)^60,61^. The toxicity index (T.I) of the investigated chemicals against mosquito larvae is then evaluated using the Sun equation^62^ which is applied as follows:

$$\text{Toxicity index} = \frac{{\text{LC}}\text{50 }\text{of most effective compound}}{{\text{LC}}\text{50 }\text{of the compound used}} \, \times {100}$$

### Neural receptors 3D structure preparations

The amino acid sequences for the target receptors of the house mosquito (*C. pipiens*): acetylcholinesterase (AChE) (Accession Number: Q86GC8), nicotinic acetylcholine receptor (nAChR) (Accession Number: A0A8D8NUM7), and voltage-gated sodium channel alpha subunit (VGSC α subunit) (Accession Number: A0A8D8AMN4), were obtained from the UniProt Knowledgebase (UniProtKB) (https://www.uniprot.org/).

Due to the potential limitations of using pre-existing structures, homology modeling was employed to generate 3D models for the AChE enzyme, the nAChR, and the VGSC α subunit binding proteins. SWISS-MODEL (https://swissmodel.expasy.org/), a web-based server for protein structure prediction, was used for this purpose^48,63^. This server utilizes a combination of BLASTp and HHBlits algorithms to identify suitable template structures within the Protein Data Bank (PDB) and SWISS-MODEL Template Library (SMTL) databases for each receptor^48^. The identified templates are then used to build a reliable model for the target protein sequence.

The quality of the generated homology models was evaluated using the Z-scoring functions, General Model Quality Estimate (GMQE), and Qualitative Model Energy Analysis (QMEAN), which are specifically designed for SWISS-MODEL outputs^28,48,63^. These scores provide an objective assessment of the model's accuracy and reliability.

### Molecular docking assessment

A molecular docking simulation was performed to understand the potential mode of action underlying the larvicidal activity of the tested compounds. The 2D structures of the fifteen compounds were drawn using ChemDraw 20.0 (CambridgeSoft). The Molecular Operating Environment (MOE V. 2014.02 software; https://www.chemcomp.com/en/index.htm) was then employed for 3D structure generation of the compounds, protonation state assignment, partial charge calculation, and energy minimization. Geometry optimization and energy minimization of the 3D structures were further performed using Wave Function Spartan v 14.0 (Wavefunction Inc., Irvine, CA, USA) to improve docking accuracy^26^.

The 3D structures of the target receptors (AChE,nAChRs, and VGSC α subunit) were prepared and used as receptors^28^. The MOE-Site-Finder function was used to define the active site for docking with alpha spheres. A non-bonded cut-off value of 8–10 Å was applied to the Lennard–Jones terms. The energy of the complex was minimized using the MMFF94x force field until the root-mean-square (RMS) gradient reached 0.1 kcal/mol/Å. For each compound-receptor pair, 100 docking poses were generated. The ten poses with the lowest docking energies for each molecule were selected for further analysis. The London ΔG energy scoring function was employed to rank and evaluate the binding affinity of each ligand-receptor complex.

MOE software was used for cross-docking of three reference insecticides: Chlorpyrifos (AChE inhibitor), Nitenpyram (nAChRs agonist), and Indoxacarb (VGSC α subunit blocker) at the alpha pockets of these receptors. The fifteen synthesized compounds were also docked against the same three receptor pockets. Docking scores of the synthesized compounds were compared to the corresponding reference insecticide for each receptor.

### Ethics declaration

This study was approved by the Research Ethics Committee at Ain Shams University (Approval code: ASU-SCI/ENTO/2024/1/4) and was performed in accordance with the guidelines of the National Institute of Health (NIH). All methods are reported in accordance with ARRIVE guidelines.

## Conclusion

A novel series of substituted benzochromene, benzochromenopyrimidine, and benzochromenotriazolopyrimidine derivatives were designed and synthesized. Their chemical structures were confirmed using various spectroscopic techniques. Fifteen of these compounds were tested for larvicidal activity against *C. pipiens* larvae and all exhibited significant insecticidal activity compared to the conventional insecticide Chlorpyrifos. Molecular docking studies were performed to elucidate the mechanisms behind the observed larval mortality, and the results suggested that many of the synthesized compounds act as potential insect nerve poisons. Specifically, they were identified as potential acetylcholine esterase inhibitors, which can disrupt the normal nerve physiology of mosquitoes by inhibiting this enzyme, ultimately leading to insect death. These findings offer a promising avenue for combating the insecticidal resistance developed by disease vector mosquitoes against a wide range of conventional insecticides. However, further in vitro and in vivo investigations are necessary to validate these results and to assess the potential impact of these compounds on higher vertebrates.

### Supplementary Information

Supplementary Information 1.

Supplementary Information 2.

## Data Availability

All data generated or analyzed during this study are included in this published article and its supplementary information files.
