# Supplementary material for: Synthesis, molecular modelling and evaluation of larvicidal efficacy of annulated Benzo[h]chromenes against Culex pipiens L. Larvae
Source: Sci Rep. 2024 Aug 8;14:18393. doi: 10.1038/s41598-024-68035-0 (PMC11310521; doi:10.1038/s41598-024-68035-0)
Supplement: Supplementary file 1 — Supplementary Information 1. [file 41598_2024_68035_MOESM1_ESM.pdf]

S. File1: The 15 novel compounds exhibited a 2D interaction profile against three key neural receptors in *Culex pipiens;* Acetylcholine Esterase Enzyme (AChE), nicotinic acetylcholine receptors binding protein (nAChRs), and voltage-gated sodium channels binding protein α subunit (VGSC α subunit) that was comparable to, or even exceeded, the inhibitory patterns of known insecticides targeting the same receptors.

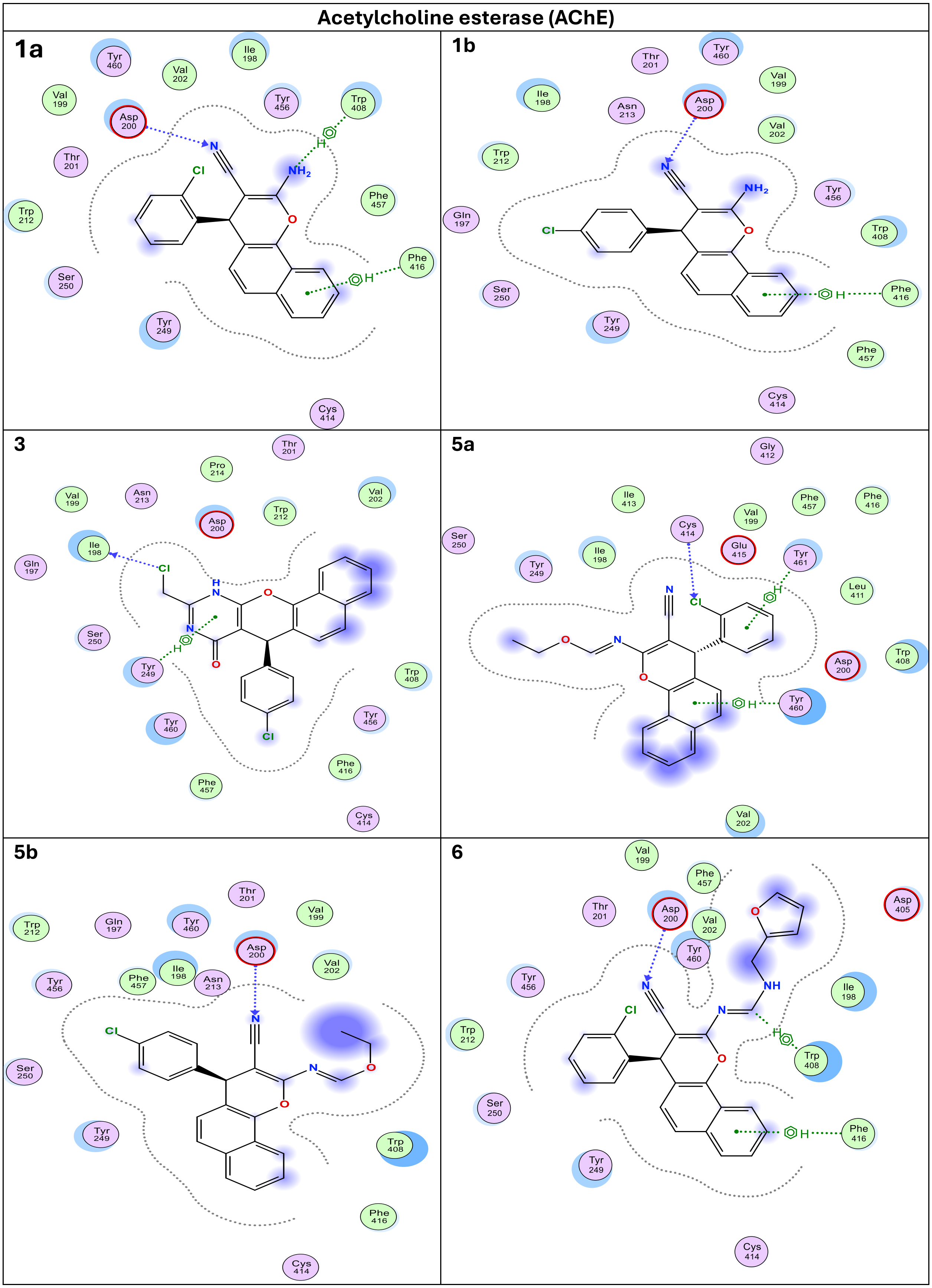

Acetylcholine esterase (AChE)
1a
Tyr 460
Ile 198
Val 202
Val 199
Tyr 456
Trp 408
Asp 200
Thr 201
Trp 212
Phe 457
Phe 416
Ser 250
Tyr 249
Cys 414
N
NH2
H
Cl
O
1b
Thr 201
Tyr 460
Val 199
Ile 198
Asn 213
Asp 200
Val 202
Trp 212
Tyr 456
Gln 197
Trp 408
Ser 250
Phe 416
Tyr 249
Phe 457
Cys 414
N
NH2
Cl
O
H
3
Thr 201
Pro 214
Val 202
Val 199
Asn 213
Asp 200
Trp 212
Ile 198
Gln 197
Ser 250
Tyr 249
Trp 408
Tyr 460
Tyr 456
Phe 416
Phe 457
Cys 414
Cl
H
N
O
5a
Gly 412
Ile 413
Phe 457
Phe 416
Ser 250
Cys 414
Val 199
Glu 415
Ile 198
Tyr 249
Tyr 461
Leu 411
Asp 200
Trp 408
Tyr 460
Val 202
N
Cl
H
O
5b
Thr 201
Trp 212
Gln 197
Tyr 460
Val 199
Asp 200
Val 202
Tyr 456
Ile 198
Phe 457
Asn 213
Ser 250
Tyr 249
Trp 408
Phe 416
Cys 414
Cl
N
O
6
Val 199
Phe 457
Thr 201
Asp 200
Val 202
Asp 405
Tyr 460
Tyr 456
Ile 198
Trp 212
Trp 408
Ser 250
Phe 416
Tyr 249
Cys 414
NH
N
Cl
O
H

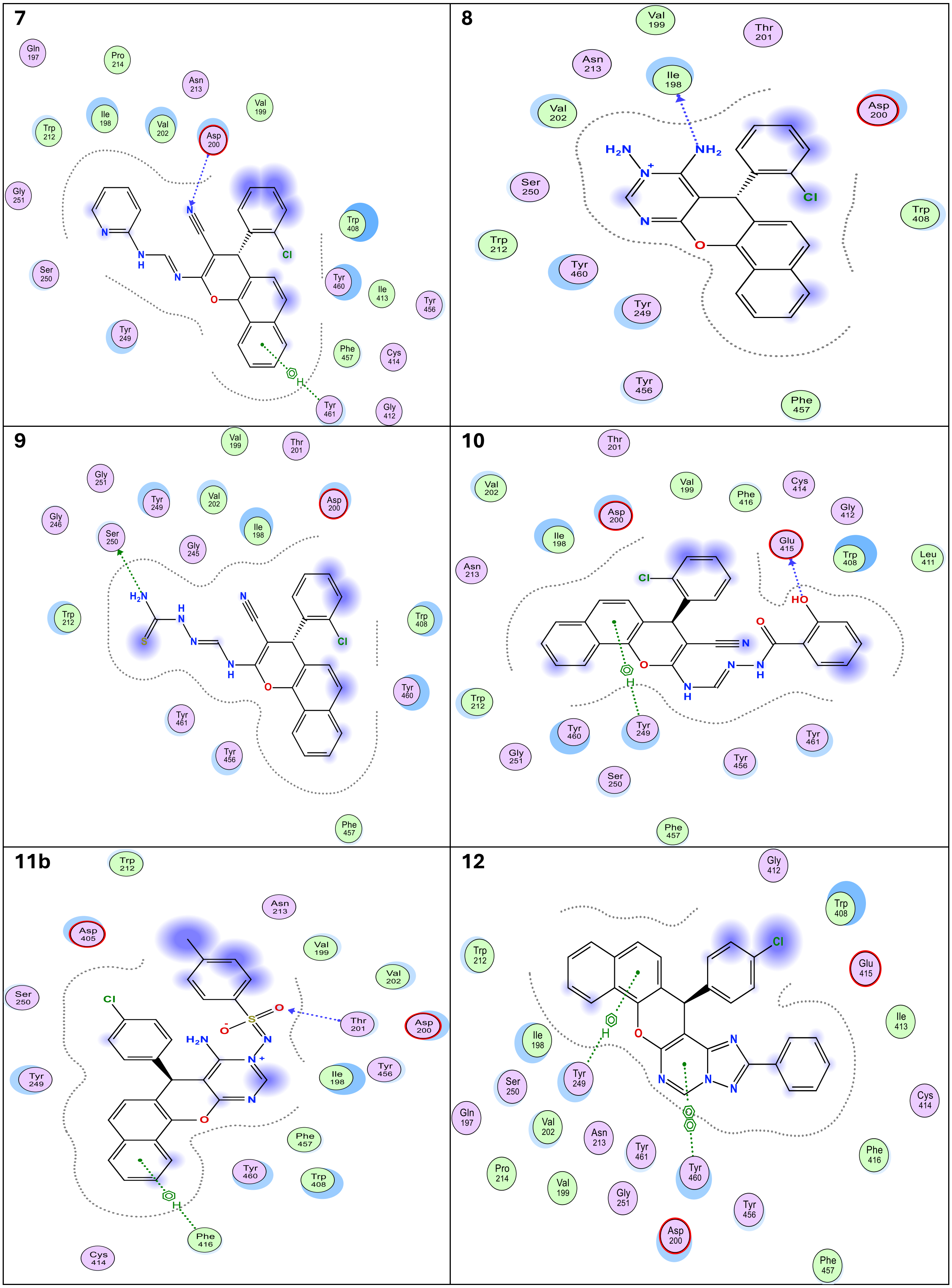
7
Gln 197
Pro 214
Asn 213
Val 199
Trp 212
Ile 198
Val 202
Asp 200
Gly 251
Trp 408
Ser 250
Tyr 460
Ile 413
Tyr 456
Tyr 249
Phe 457
Cys 414
Tyr 461
Gly 412
8
Val 199
Thr 201
Asn 213
Ile 198
Val 202
Asp 200
Ser 250
Trp 408
Trp 212
Tyr 460
Tyr 249
Tyr 456
Phe 457
9
Val 199
Thr 201
Gly 251
Tyr 249
Val 202
Asp 200
Gly 246
Ser 250
Gly 245
Ile 198
Trp 212
Trp 408
Tyr 460
Tyr 461
Tyr 456
Phe 457
10
Thr 201
Val 202
Val 199
Phe 416
Cys 414
Asp 200
Gly 412
Ile 198
Glu 415
Trp 408
Leu 411
Asn 213
Trp 212
Tyr 460
Tyr 249
Tyr 461
Gly 251
Tyr 456
Ser 250
Phe 457
11b
Trp 212
Asn 213
Asp 405
Val 199
Val 202
Ser 250
Thr 201
Asp 200
Ile 198
Tyr 456
Tyr 249
Phe 457
Tyr 460
Trp 408
Phe 416
Cys 414
12
Gly 412
Trp 408
Trp 212
Glu 415
Ile 413
Ile 198
Ser 250
Tyr 249
Cys 414
Gln 197
Val 202
Asn 213
Tyr 461
Phe 416
Pro 214
Val 199
Gly 251
Tyr 460
Tyr 456
Asp 200
Phe 457

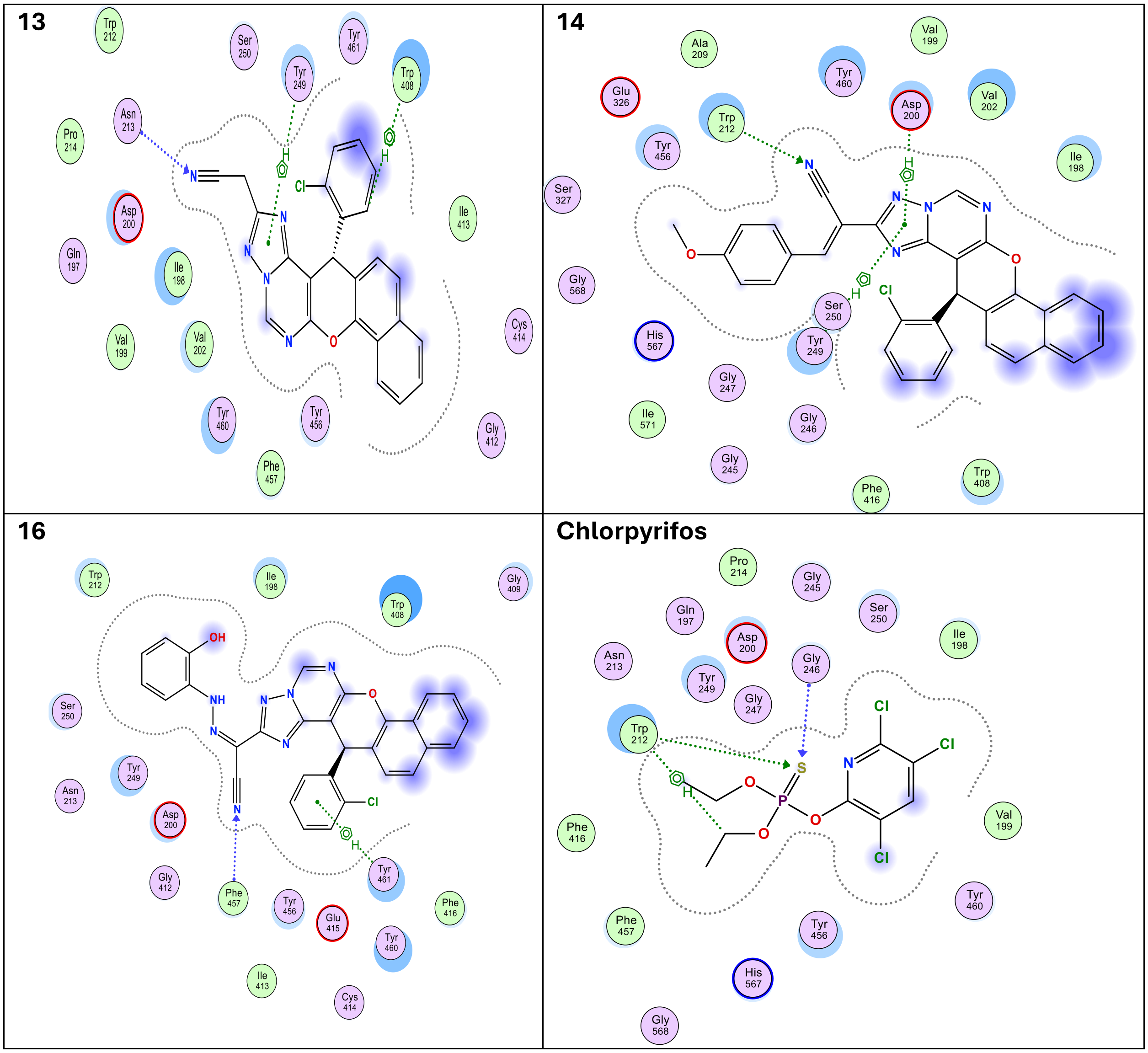
13
Trp 212
Ser 250
Tyr 461
Tyr 249
Trp 408
Asn 213
Pro 214
Asp 200
Gln 197
Ile 198
Ile 413
Val 199
Val 202
Cys 414
Tyr 460
Tyr 456
Gly 412
Phe 457
14
Ala 209
Val 199
Tyr 460
Glu 326
Val 202
Asp 200
Trp 212
Tyr 456
Ile 198
Ser 327
Gly 568
Ser 250
His 567
Tyr 249
Gly 247
Ile 571
Gly 246
Gly 245
Phe 416
Trp 408
16
Trp 212
Ile 198
Trp 408
Gly 409
Ser 250
Tyr 249
Asn 213
Asp 200
Gly 412
Phe 457
Tyr 456
Glu 415
Tyr 461
Tyr 460
Phe 416
Ile 413
Cys 414
Chlorpyrifos
Pro 214
Gly 245
Gln 197
Ser 250
Ile 198
Asn 213
Asp 200
Gly 246
Tyr 249
Gly 247
Trp 212
Phe 416
Val 199
Phe 457
Tyr 456
Tyr 460
His 567
Gly 568

AChE						
**Ligand**	**Ligand interacting atoms**	**Receptor Interacting atoms**	**Receptor Interacting Residues**	**Interaction Bond Type**	**Distance**	**E (kcal/mol)**
**1a**	N 37	CA	ASP 200 (B)	H-acceptor	3.52	-0.8
	N 23	6-ring	TRP 408 (B)	H-pi	4.32	-0.5
	6-ring	CE1	PHE 416 (B)	pi-H	4.19	-0.6
**1b**	N 37	CA	ASP 200 (B)	H-acceptor	3.59	-0.9
	6-ring	CE1	PHE 416 (B)	pi-H	4.36	-0.5
**3**	CL 32	O	ILE 198 (B)	H-donor	3.18	-0.5
	6-ring	CE1	TYR 249 (B)	pi-H	3.61	-0.9
**5a**	CL 43	N	CYS 414 (B)	H-acceptor	3.47	-0.5
	6-ring	CB	TYR 460 (B)	pi-H	4.6	-0.5
	6-ring	CD2	TYR 461 (B)	pi-H	4.14	-0.7
**5b**	N 27	CA	ASP 200 (B)	H-acceptor	3.51	-1
**6**	N 39	CA	ASP 200 (B)	H-acceptor	3.48	-0.8
	C 1	6-ring	TRP 408 (B)	H-pi	3.52	-0.7
	6-ring	CE1	PHE 416 (B)	pi-H	4.21	-0.7
**7**	N 49	CA	ASP 200 (B)	H-acceptor	3.6	-0.6
	6-ring	CB	TYR 461 (B)	pi-H	4.63	-0.6
**8**	N 28	O	ILE 198 (B)	H-donor	3.14	-0.6
**9**	N 23	OG	SER 250 (B)	H-donor	3.13	-0.8
**10**	O 52	O	GLU 415 (B)	H-donor	2.72	-2.3
	6-ring	CE1	TYR 249 (B)	pi-H	3.72	-1.1
**11b**	O 39	N	THR 201 (B)	H-acceptor	3.15	-2.9
	6-ring	CE1	PHE 416 (B)	pi-H	4.26	-0.5
**12**	6-ring	CE1	TYR 249 (B)	pi-H	4	-1
	6-ring	6-ring	TYR 460 (B)	pi-pi	3.42	0
**13**	N 45	CA	ASN 213 (B)	H-acceptor	3.6	-0.8
	C 34	5-ring	TRP 408 (B)	H-pi	3.89	-0.5
	C 34	6-ring	TRP 408 (B)	H-pi	4.16	-0.7
	5-ring	CE1	TYR 249 (B)	pi-H	3.53	-0.5
**14**	N 55	CB	TRP 212 (B)	H-acceptor	3.33	-0.5
	5-ring	CB	ASP 200 (B)	pi-H	3.87	-0.6
	5-ring	CE1	TYR 249 (B)	pi-H	3.48	-0.7
**16**	N 58	CA	PHE 457 (B)	H-acceptor	3.59	-0.8
	6-ring	CD2	TYR 461 (B)	pi-H	4.04	-0.9
**Chlorpyrifos**	S 4	CB	TRP 212 (B)	H-acceptor	3.72	-0.7
	S 4	CA	GLY 246 (B)	H-acceptor	3.78	-1.2
	C 11	6-ring	TRP 212 (B)	H-pi	3.89	-0.6

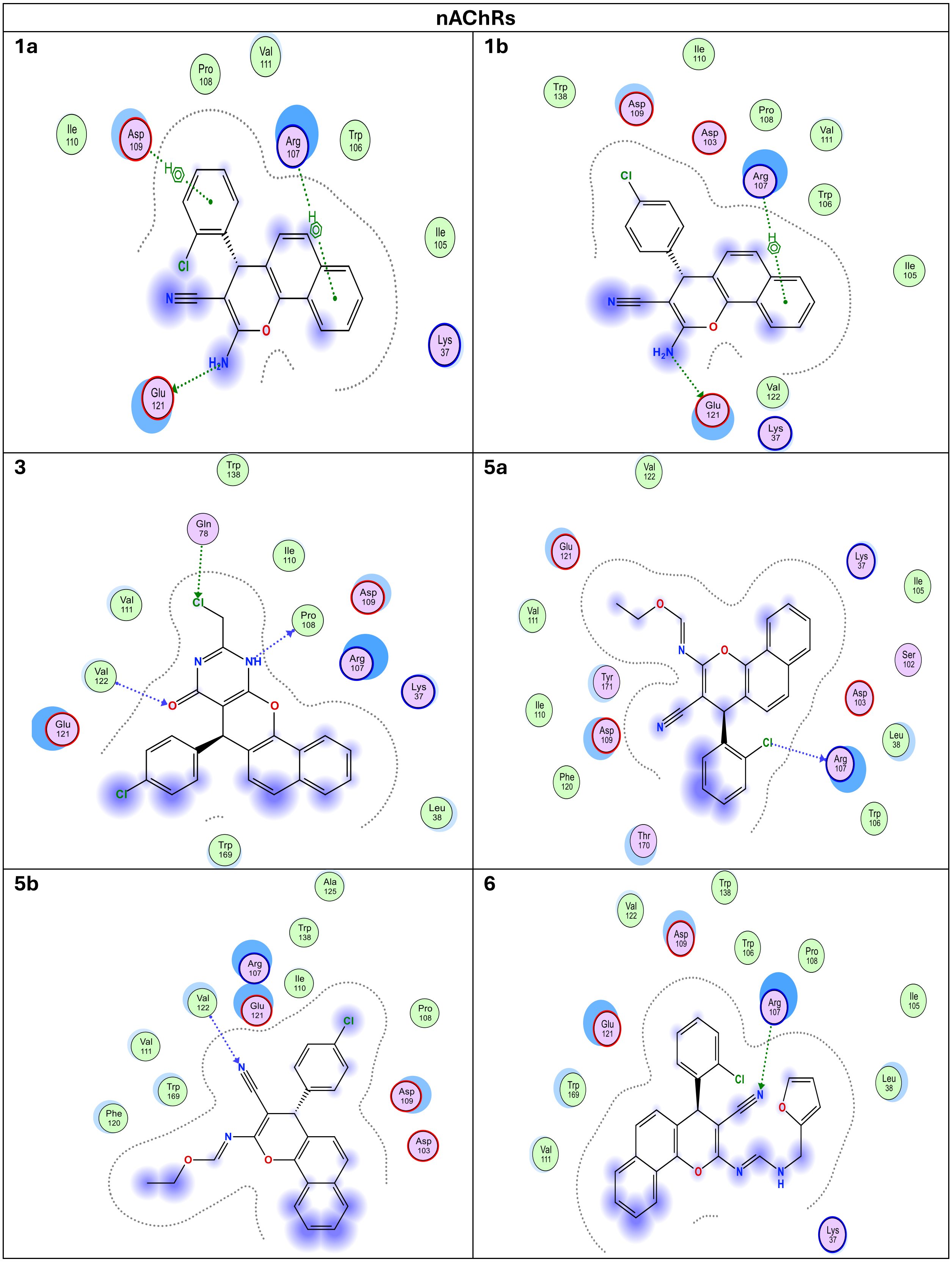
nAChRs
1a
Val 111
Pro 108
Ile 110
Asp 109
Arg 107
Trp 106
Ile 105
Lys 37
Glu 121
Cl
N
O
H2N
1b
Ile 110
Trp 138
Asp 109
Pro 108
Asp 103
Val 111
Arg 107
Trp 106
Ile 105
Val 122
Glu 121
Lys 37
Cl
N
O
H2N
3
Trp 138
Gln 78
Ile 110
Val 111
Asp 109
Pro 108
Arg 107
Val 122
Lys 37
Glu 121
Leu 38
Trp 169
Cl
N
NH
O
5a
Val 122
Glu 121
Lys 37
Ile 105
Val 111
Ser 102
Tyr 171
Asp 103
Ile 110
Asp 109
Leu 38
Phe 120
Arg 107
Trp 106
Thr 170
O
N
Cl
5b
Ala 125
Trp 138
Arg 107
Ile 110
Val 122
Glu 121
Pro 108
Val 111
Trp 169
Asp 109
Phe 120
Asp 103
Cl
N
O
6
Trp 138
Val 122
Asp 109
Trp 106
Pro 108
Arg 107
Ile 105
Glu 121
Trp 169
Leu 38
Val 111
Lys 37
Cl
N
O
H

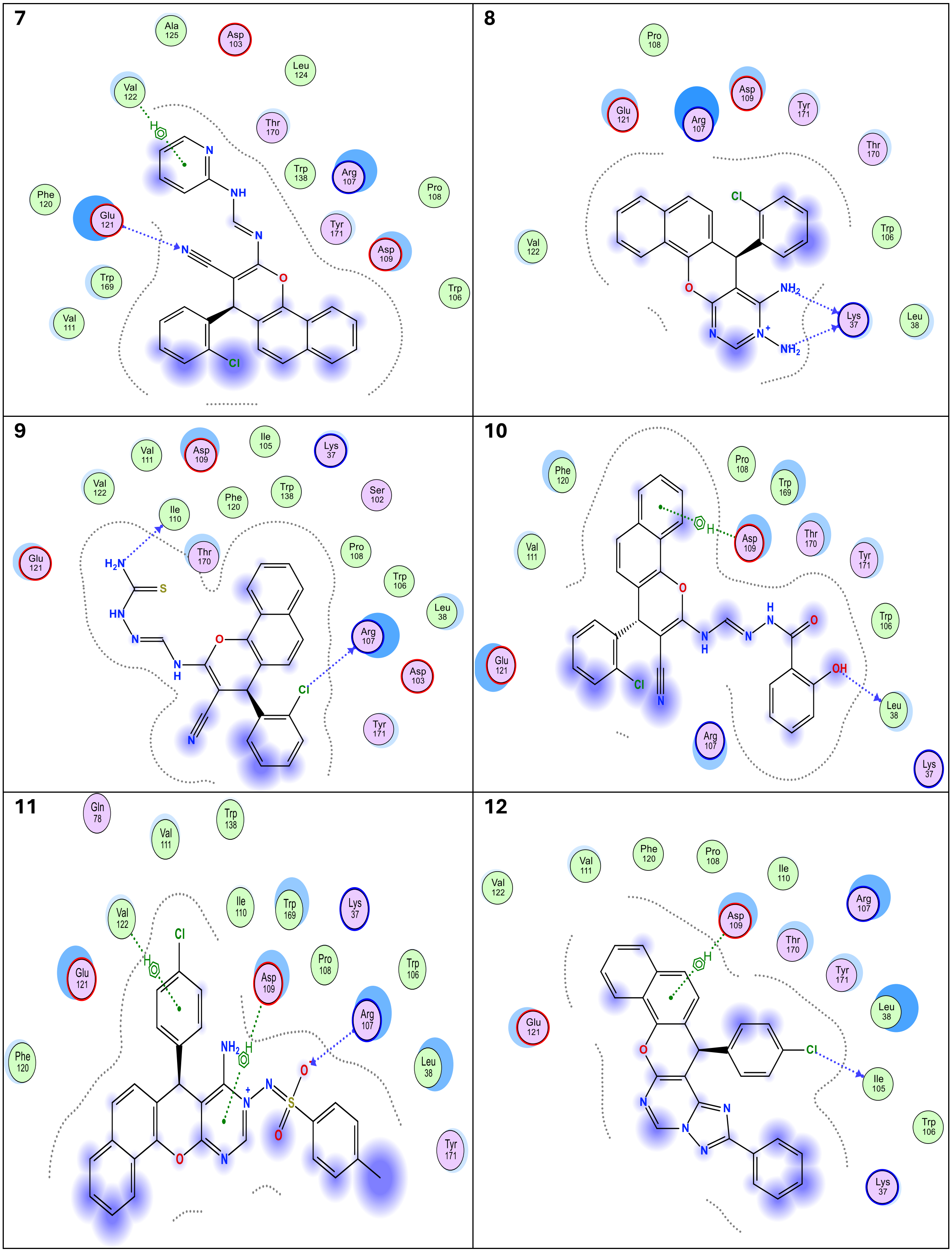

7
Ala 125
Asp 103
Leu 124
Val 122
Thr 170
Trp 138
Arg 107
Pro 108
Phe 120
Glu 121
Tyr 171
Asp 109
Trp 169
Trp 106
Val 111
N
NH
N
N
O
Cl
H
8
Pro 108
Glu 121
Arg 107
Asp 109
Tyr 171
Thr 170
Val 122
Trp 106
Lys 37
Leu 38
Cl
O
NH_2
N
N^+
NH_2
9
Val 111
Asp 109
Ile 105
Lys 37
Val 122
Phe 120
Trp 138
Ser 102
Ile 110
Glu 121
Thr 170
Pro 108
Trp 106
Leu 38
Arg 107
Asp 103
Tyr 171
H_2N
S
HN
N
O
N
H
Cl
N
10
Pro 108
Phe 120
Trp 169
Val 111
Asp 109
Thr 170
Tyr 171
Trp 106
Glu 121
Leu 38
Arg 107
Lys 37
H
O
N
H
N
H
N
O
OH
Cl
N
11
Gln 78
Val 111
Trp 138
Ile 110
Trp 169
Lys 37
Val 122
Glu 121
Cl
Pro 108
Asp 109
Trp 106
Arg 107
Leu 38
Phe 120
Tyr 171
NH_2
H
N^+
N
O
S
O
O
N
H
12
Val 122
Val 111
Phe 120
Pro 108
Ile 110
Arg 107
Asp 109
Thr 170
Tyr 171
Leu 38
Glu 121
Cl
Ile 105
Trp 106
Lys 37
O
H
N
N
N
N

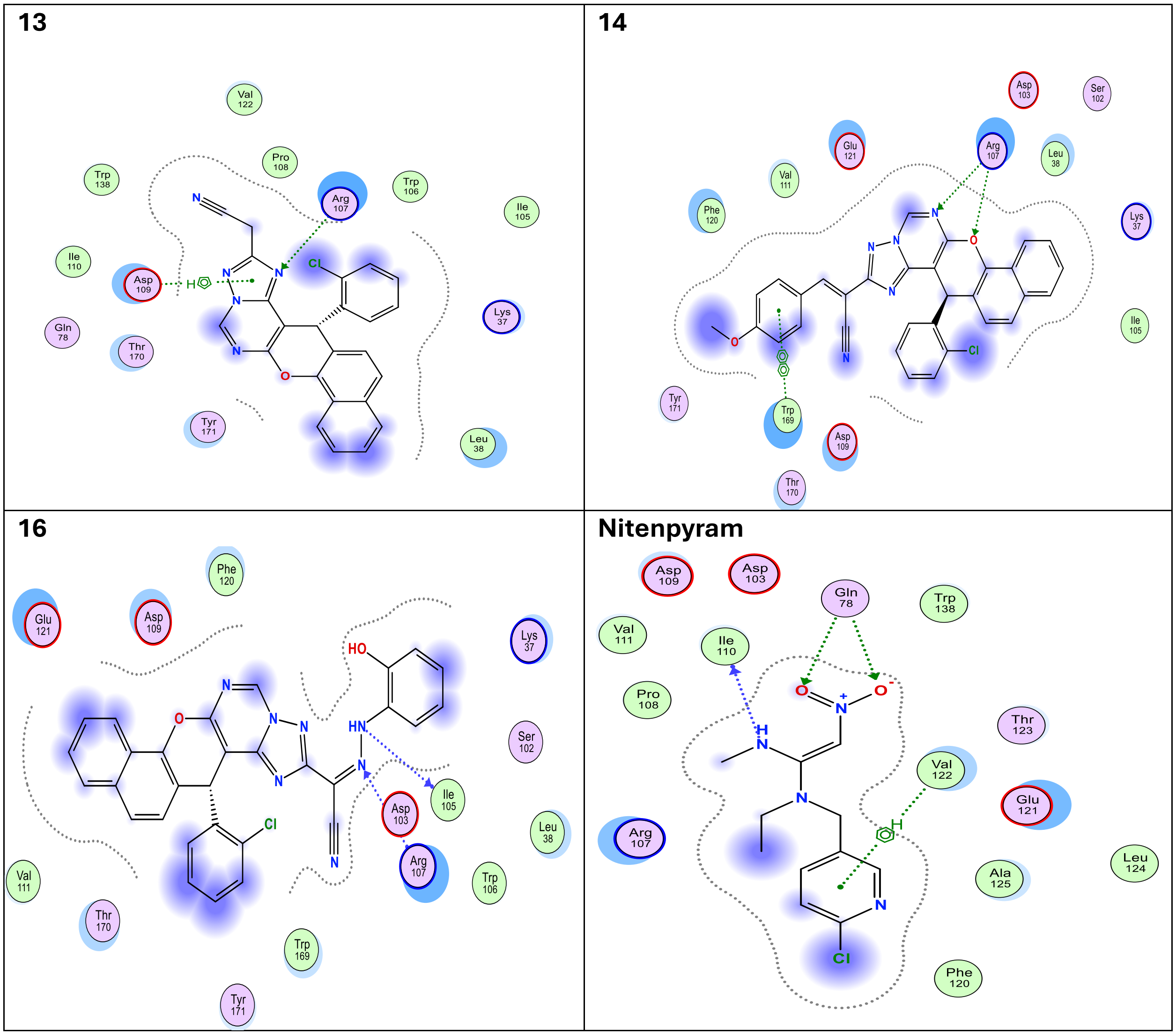

13
Val 122
Pro 108
Trp 138
Arg 107
Trp 106
Ile 105
Ile 110
Asp 109
Gln 78
Thr 170
Lys 37
Tyr 171
Leu 38
14
Asp 103
Ser 102
Glu 121
Arg 107
Leu 38
Val 111
Phe 120
Lys 37
Ile 105
Tyr 171
Trp 169
Asp 109
Thr 170
16
Phe 120
Glu 121
Asp 109
Lys 37
Ser 102
Ile 105
Asp 103
Leu 38
Arg 107
Trp 106
Val 111
Thr 170
Trp 169
Tyr 171
Nitenpyram
Asp 109
Asp 103
Gln 78
Trp 138
Val 111
Ile 110
Pro 108
Thr 123
Val 122
Glu 121
Arg 107
Leu 124
Ala 125
Phe 120

nAChRs						
**Ligand**	**Ligand interacting atoms**	**Receptor Interacting atoms**	**Receptor Interacting Residues**	**Interaction Bond Type**	**Distance**	**E (kcal/mol)**
**1a**	N 23	OE1	GLU 121 (A)	H-donor	3.04	-2.8
	6-ring	CG	ARG 107 (A)	pi-H	3.8	-0.7
	6-ring	CA	ASP 109 (A)	pi-H	3.51	-0.5
**1b**	N 23	OE1	GLU 121 (A)	H-donor	2.95	-3.2
	6-ring	CG	ARG 107 (A)	pi-H	3.95	-0.5
**3**	N 14	O	PRO 108 (A)	H-donor	2.81	-3.9
	O 28	N	VAL 122 (A)	H-acceptor	3.18	-1.5
	CL 32	NE2	GLN 78 (A)	H-acceptor	3.16	-0.7
**5a**	CL 43	O	ARG 107 (A)	H-donor	3.94	-0.5
**5b**	N 27	N	VAL 122 (A)	H-acceptor	3.52	-0.8
**6**	N 39	NH2	ARG 107 (A)	H-acceptor	3.07	-0.7
**7**	N 49	CA	GLU 121 (A)	H-acceptor	3.65	-0.5
	6-ring	CB	VAL 122 (A)	pi-H	4.27	-0.6
**8**	N 28	O	LYS 37 (A)	H-donor	3.41	-1.7
	N 31	O	LYS 37 (A)	H-donor	3.35	-0.7
**9**	N 23	O	ILE 110 (A)	H-donor	3.38	-0.5
	CL 44	O	ARG 107 (A)	H-donor	3.42	-2
**10**	O 52	O	LEU 38 (A)	H-donor	3.21	-1.3
	6-ring	CA	ASP 109 (A)	pi-H	3.8	-0.9
**11b**	O 40	N	ARG 107 (A)	H-acceptor	3.39	-1.2
	6-ring	CB	ASP 109 (A)	pi-H	3.68	-1.1
	6-ring	N	VAL 122 (A)	pi-H	4.78	-0.6
**12**	CL 40	O	ILE 105 (A)	H-donor	3.15	-0.5
	6-ring	CA	ASP 109 (A)	pi-H	3.88	-0.7
**13**	N 22	NE	ARG 107 (A)	H-acceptor	3.16	-0.9
	5-ring	CA	ASP 109 (A)	pi-H	3.77	-0.7
**14**	O 9	NH2	ARG 107 (A)	H-acceptor	3.21	-0.8
	N 14	NH2	ARG 107 (A)	H-acceptor	3.59	-0.6
	6-ring	6-ring	TRP 169 (A)	pi-pi	3.9	0
**16**	N 45	O	ILE 105 (A)	H-donor	3.54	-1.1
	N 24	N	ARG 107 (A)	H-acceptor	3.45	-0.5
**Nitenpyram**	N 5	O	ILE 110 (A)	H-donor	3.19	-1.5
	O 2	NE2	GLN 78 (A)	H-acceptor	2.92	-2.1
	O 3	NE2	GLN 78 (A)	H-acceptor	3.32	-0.7
	6-ring	CB	VAL 122 (A)	pi-H	4.44	-0.6
	N 5	O	ILE 110 (A)	H-donor	3.19	-1.5

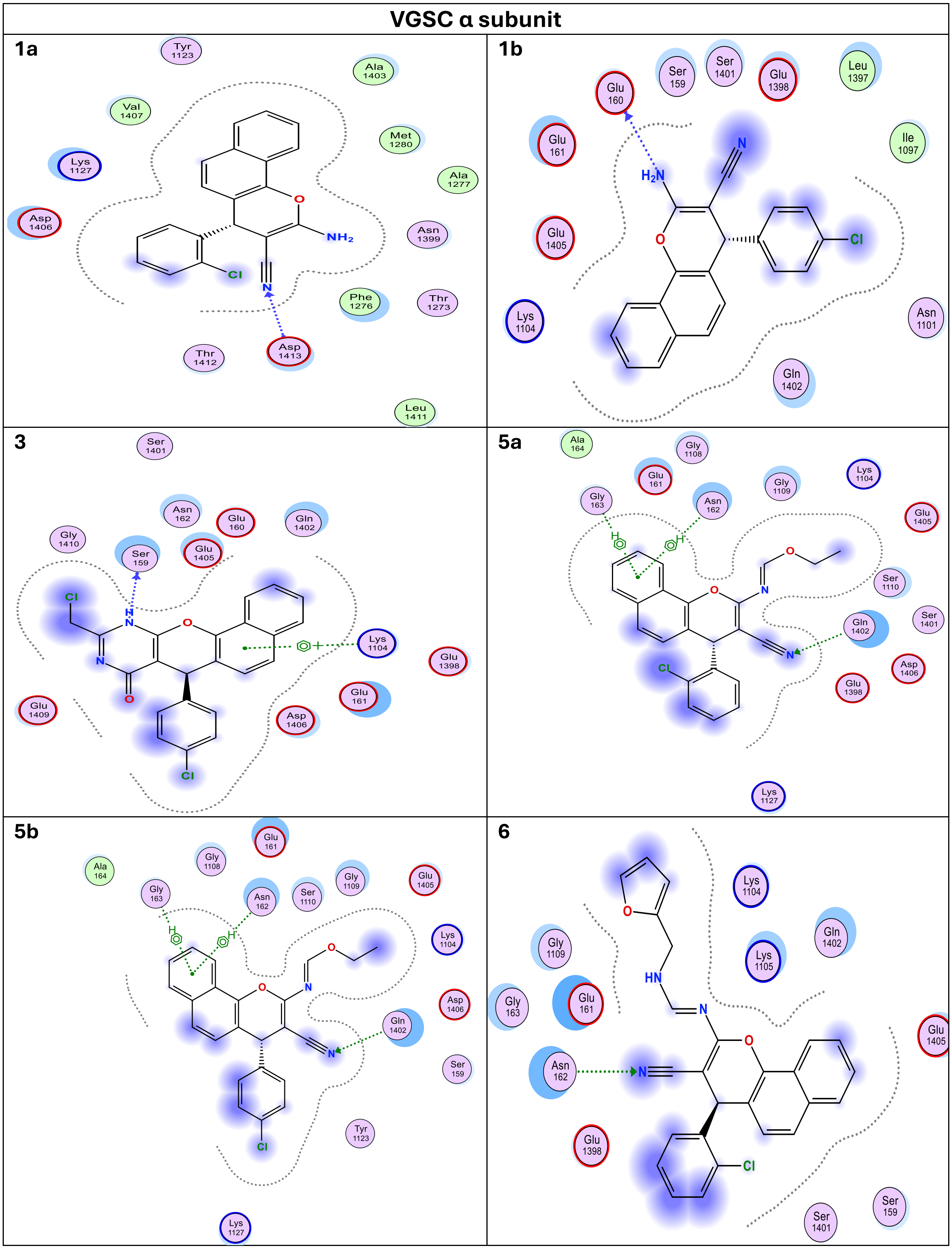

VGSC α subunit
1a
Tyr 1123
Ala 1403
Val 1407
Met 1280
Lys 1127
Ala 1277
Asp 1406
Asn 1399
O
NH2
Cl
N
Phe 1276
Thr 1273
Asp 1413
Thr 1412
Leu 1411
1b
Glu 160
Ser 159
Ser 1401
Glu 1398
Leu 1397
Glu 161
Ile 1097
N
H2N
Glu 1405
O
Cl
Lys 1104
Asn 1101
Gln 1402
3
Ser 1401
Asn 162
Glu 160
Gln 1402
Gly 1410
Ser 159
Glu 1405
Cl
H
N
O
Lys 1104
N
Glu 1398
O
Glu 161
Glu 1409
Asp 1406
Cl
5a
Ala 164
Gly 1108
Glu 161
Gly 1109
Lys 1104
Gly 163
Asn 162
Glu 1405
H
H
O
N
O
Ser 1110
Ser 1401
Gln 1402
Cl
N
Asp 1406
Glu 1398
Lys 1127
5b
Glu 161
Ala 164
Gly 1108
Gly 163
Asn 162
Ser 1110
Gly 1109
Glu 1405
H
H
O
Lys 1104
O
N
Asp 1406
Gln 1402
N
Ser 159
Cl
Tyr 1123
Lys 1127
6
Lys 1104
O
Gln 1402
Gly 1109
HN
Lys 1105
Gly 163
Glu 161
N
O
Glu 1405
Asn 162
N
Glu 1398
Cl
Ser 1401
Ser 159

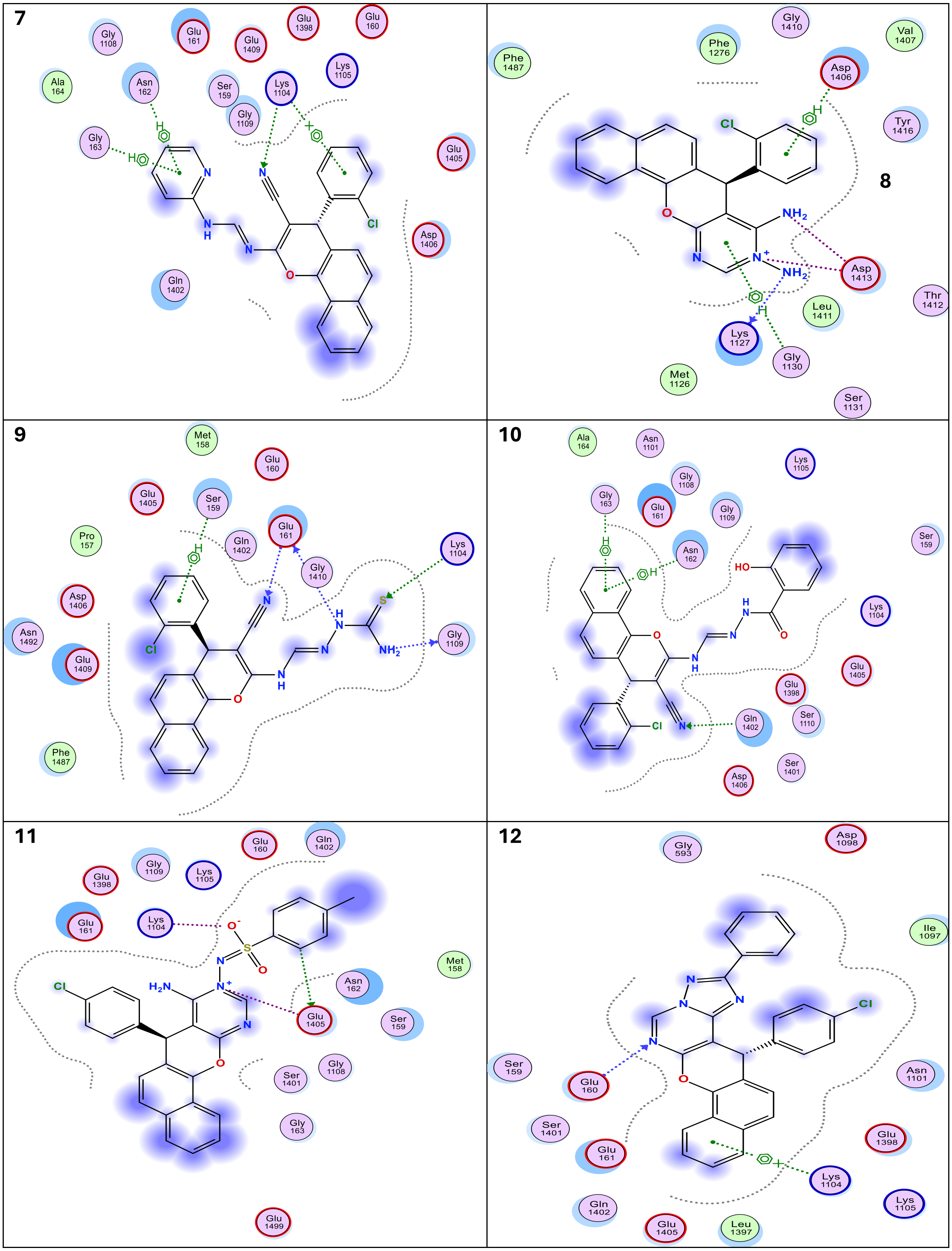

7
Gly 1108
Glu 161
Glu 1409
Glu 1398
Glu 160
Lys 1105
Ala 164
Asn 162
Ser 159
Lys 1104
Gly 1109
Gly 163
Glu 1405
Asp 1406
Gln 1402
N
H
N
O
Cl
8
Gly 1410
Val 1407
Phe 1276
Phe 1487
Asp 1406
Tyr 1416
Cl
H
O
NH_2
N
N^+
Asp 1413
Thr 1412
Leu 1411
Lys 1127
Gly 1130
Met 1126
Ser 1131
9
Met 158
Glu 160
Glu 1405
Ser 159
Gln 1402
Glu 161
Pro 157
Gly 1410
Lys 1104
Asp 1406
S
Asn 1492
Cl
NH_2
Gly 1109
Glu 1409
Phe 1487
10
Ala 164
Asn 1101
Lys 1105
Gly 1108
Gly 163
Glu 161
Gly 1109
Ser 159
Asn 162
HO
Lys 1104
Glu 1405
Glu 1398
Ser 1110
Gln 1402
Cl
Ser 1401
Asp 1406
11
Glu 160
Gln 1402
Gly 1109
Lys 1105
Glu 1398
Glu 161
Lys 1104
O^-
S
Met 158
Cl
H_2N
N^+
Asn 162
Glu 1405
Ser 159
Ser 1401
Gly 1108
Gly 163
Glu 1499
12
Gly 593
Asp 1098
Ile 1097
Cl
Ser 159
Glu 160
Asn 1101
Ser 1401
Glu 1398
Glu 161
Lys 1104
Lys 1105
Gln 1402
Glu 1405
Leu 1397

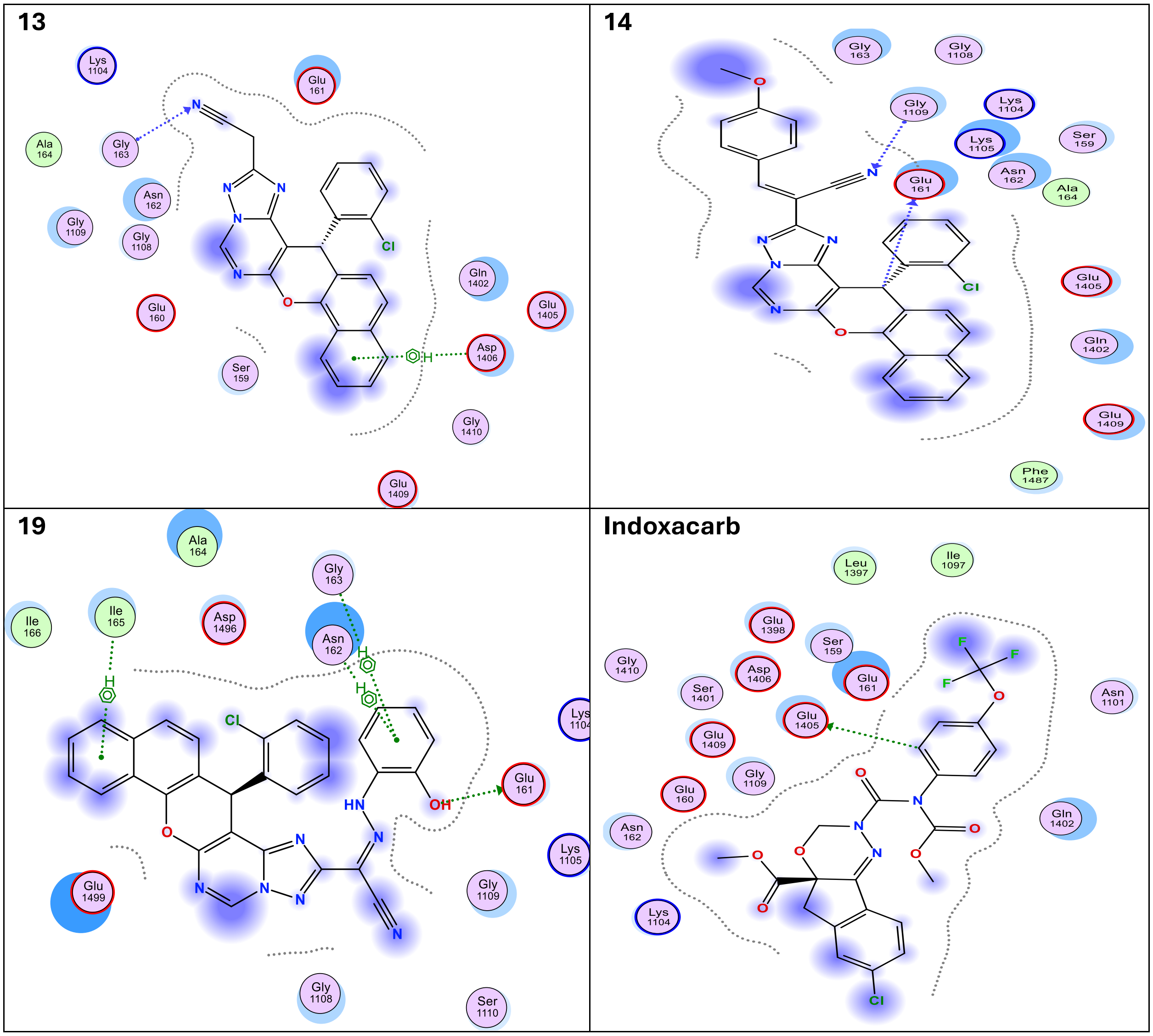
13
Lys 1104
Glu 161
Ala 164
Gly 163
Asn 162
Gly 1109
Gly 1108
Glu 160
Gln 1402
Glu 1405
Asp 1406
Ser 159
Gly 1410
Glu 1409
14
Gly 163
Gly 1108
Gly 1109
Lys 1104
Lys 1105
Ser 159
Asn 162
Glu 161
Ala 164
Glu 1405
Gln 1402
Glu 1409
Phe 1487
19
Ala 164
Ile 166
Ile 165
Asp 1496
Gly 163
Asn 162
Glu 161
Lys 1105
Gly 1109
Glu 1499
Gly 1108
Ser 1110
Indoxacarb
Leu 1397
Ile 1097
Glu 1398
Ser 159
Gly 1410
Asp 1406
Glu 161
Ser 1401
Asn 1101
Glu 1405
Glu 1409
Gly 1109
Glu 160
Asn 162
Gln 1402
Lys 1104

VGSC α subunit						
**Ligand**	**Ligand interacting atoms**	**Receptor Interacting atoms**	**Receptor Interacting Residues**	**Interaction Bond Type**	**Distance**	**E (kcal/mol)**
**1a**	N 37	N	ASP 1413 (A)	H-acceptor	3.08	-4.3
**1b**	N 23	O	GLU 160 (A)	H-donor	3.11	-1
**3**	N 14	O	SER 159 (A)	H-donor	2.95	-2.2
	6-ring	NZ	LYS 1104 (A)	pi-cation	4.41	-1
**5a**	N 45	NE2	GLN 1402 (A)	H-acceptor	3.37	-2
	6-ring	CA	ASN 162 (A)	pi-H	4.19	-1
	6-ring	N	GLY 163 (A)	pi-H	4.26	-1
**5b**	N 27	NE2	GLN 1402 (A)	H-acceptor	3.46	-1.7
	6-ring	CA	ASN 162 (A)	pi-H	4.25	-1
	6-ring	N	GLY 163 (A)	pi-H	4.29	-1.3
**6**	N 39	ND2	ASN 162 (A)	H-acceptor	3.32	-1.5
**7**	N 49	NZ	LYS 1104 (A)	H-acceptor	3.28	-1.1
	6-ring	CA	ASN 162 (A)	pi-H	4.78	-0.9
	6-ring	N	GLY 163 (A)	pi-H	4.51	-1.2
	6-ring	NZ	LYS 1104 (A)	pi-cation	4.15	-1.3
**8**	N 31	O	LYS 1127 (A)	H-donor	2.78	-3.4
	N 26	OD1	ASP 1413 (A)	Ionic	2.89	-5.3
	N 26	OD2	ASP 1413 (A)	Ionic	3.29	-2.8
	N 28	OD1	ASP 1413 (A)	Ionic	3.44	-2.1
	N 28	OD2	ASP 1413 (A)	Ionic	3.46	-2
	6-ring	CA	GLY 1130 (A)	pi-H	4.77	-0.5
	6-ring	CB	ASP 1406 (A)	pi-H	3.78	-0.6
**9**	N 20	O	GLU 161 (A)	H-donor	3.08	-2
	N 23	O	GLY 1109 (A)	H-donor	3.08	-0.8
	S 34	CE	LYS 1104 (A)	H-acceptor	4.33	-0.7
	N 46	N	GLU 161 (A)	H-acceptor	3.04	-4.3
	6-ring	N	SER 159 (A)	pi-H	4.32	-0.7
**10**	N 55	NE2	GLN 1402 (A)	H-acceptor	3.37	-1.8
	6-ring	CA	ASN 162 (A)	pi-H	4.22	-0.9
	6-ring	N	GLY 163 (A)	pi-H	4.32	-1.6
**11b**	C 32	OE1	GLU 1405 (A)	H-donor	3.26	-0.5
	N 18	OE2	GLU 1405 (A)	Ionic	3.59	-1.6
	O 40	NZ	LYS 1104 (A)	Ionic	2.96	-4.8
**12**	N 23	N	GLU 160 (A)	H-acceptor	3.14	-0.8
	6-ring	NZ	LYS 1104 (A)	pi-cation	3.9	-1.5
**13**	N 45	N	GLY 163 (A)	H-acceptor	3.39	-2.8
	6-ring	CA	ASP 1406 (A)	pi-H	4.69	-0.6

**14**	C 12	O	GLU 161 (A)	H-donor	3.31	-0.5
	N 55	CA	GLY 1109 (A)	H-acceptor	3.26	-0.7
**16**	O 43	OE2	GLU 161 (A)	H-donor	2.99	-4.4
	6-ring	CA	ASN 162 (A)	pi-H	4.39	-0.9
	6-ring	N	GLY 163 (A)	pi-H	4.43	-1.5
	6-ring	CA	ILE 165 (A)	pi-H	3.98	-0.6
**Indoxacarb**	C 30	OE1	GLU 1405 (A)	H-donor	3.44	-0.8
