# Supplementary material for: Synthesis, molecular modelling and evaluation of larvicidal efficacy of annulated Benzo[h]chromenes against Culex pipiens L. Larvae
Source: Sci Rep. 2024 Aug 8;14:18393. doi: 10.1038/s41598-024-68035-0 (PMC11310521; doi:10.1038/s41598-024-68035-0)
Supplement: Supplementary file 2 — Supplementary Information 2. [file 41598_2024_68035_MOESM2_ESM.docx]

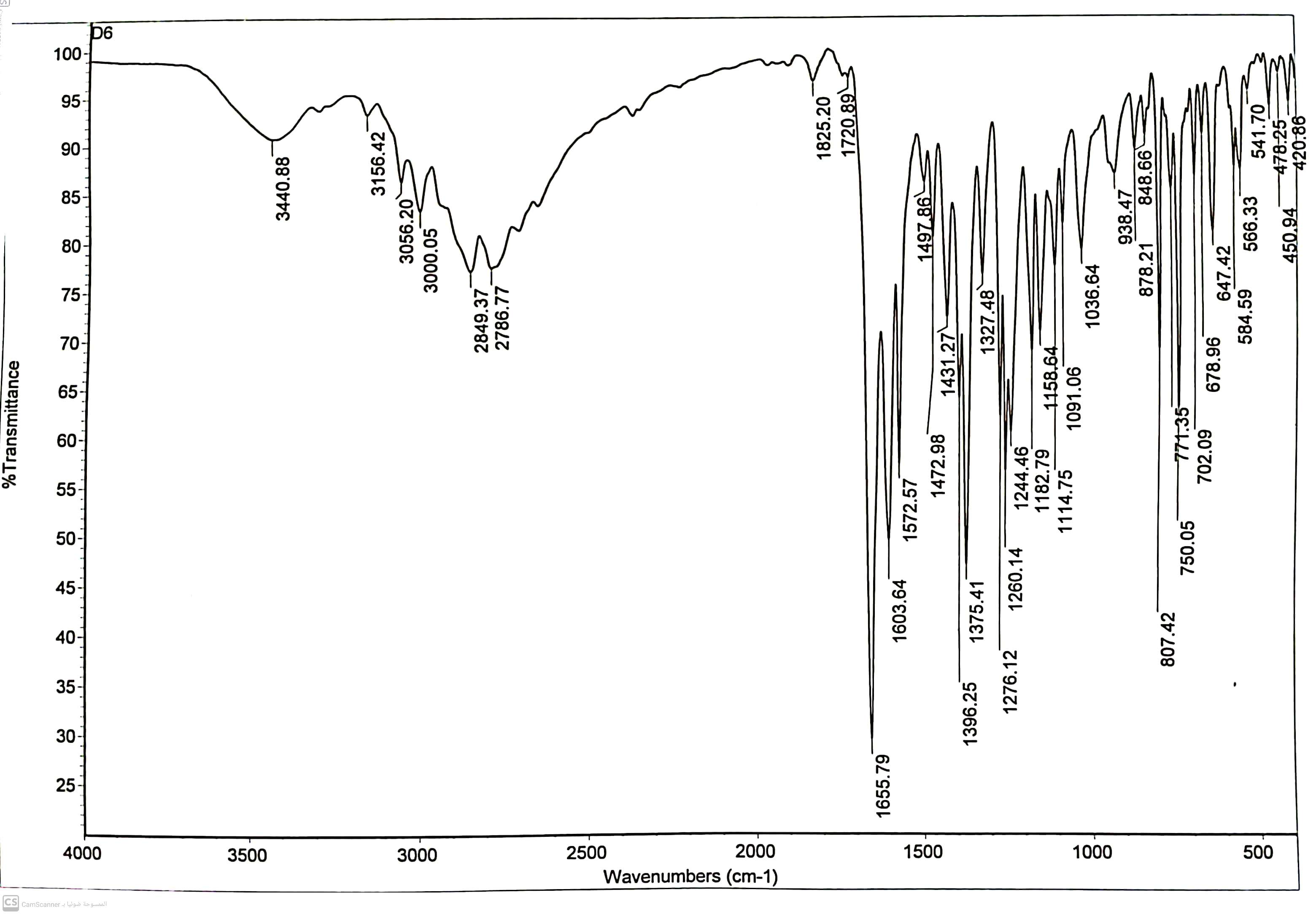

**IR spectrum of compound (2)**


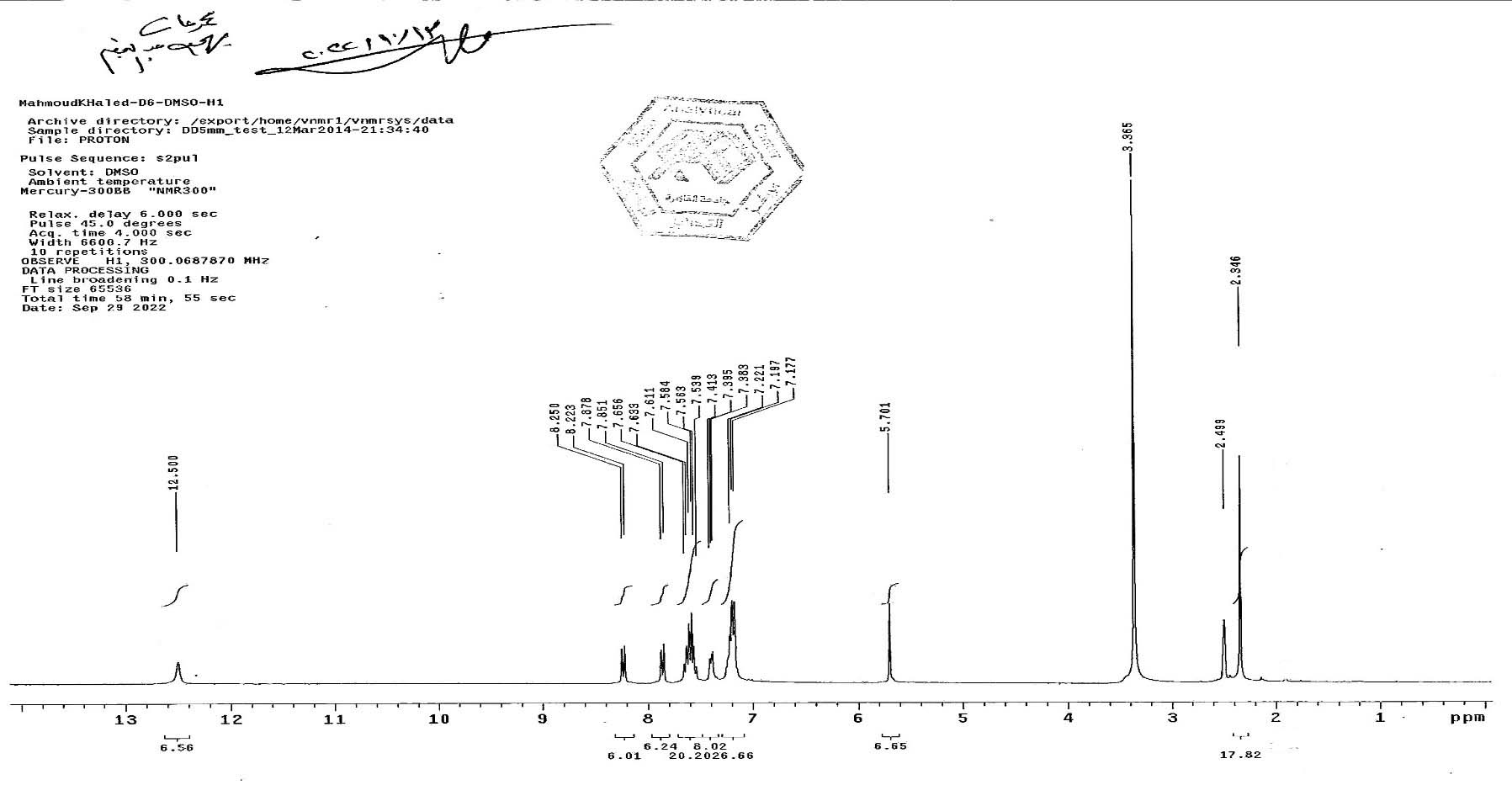

**^1^H-NMR (DMSO- d_6_) of Compound (2)**


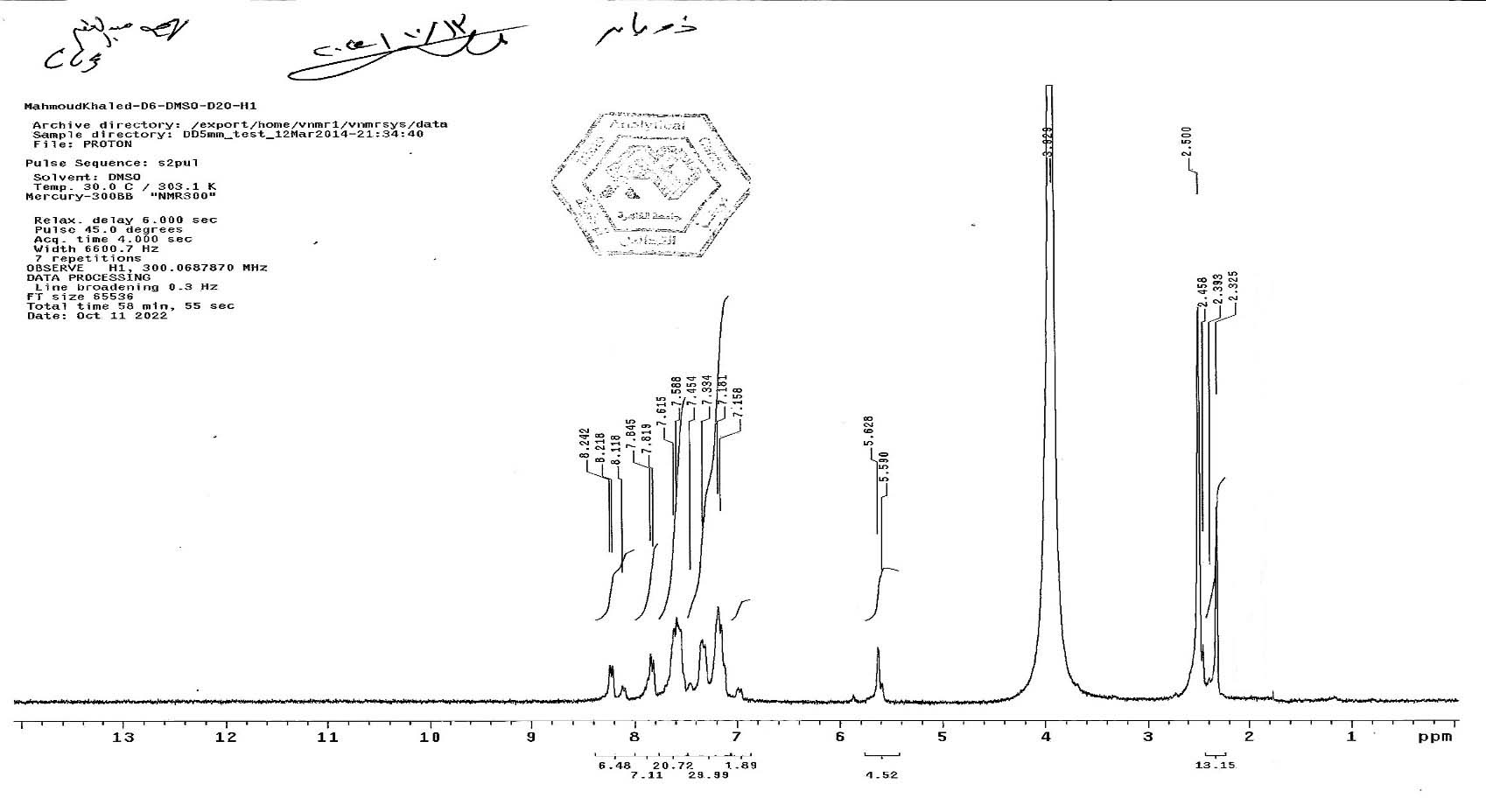

**^1^H-NMR spectrum (DMSO-d_6_ + D_2_O) of Compound (2)**


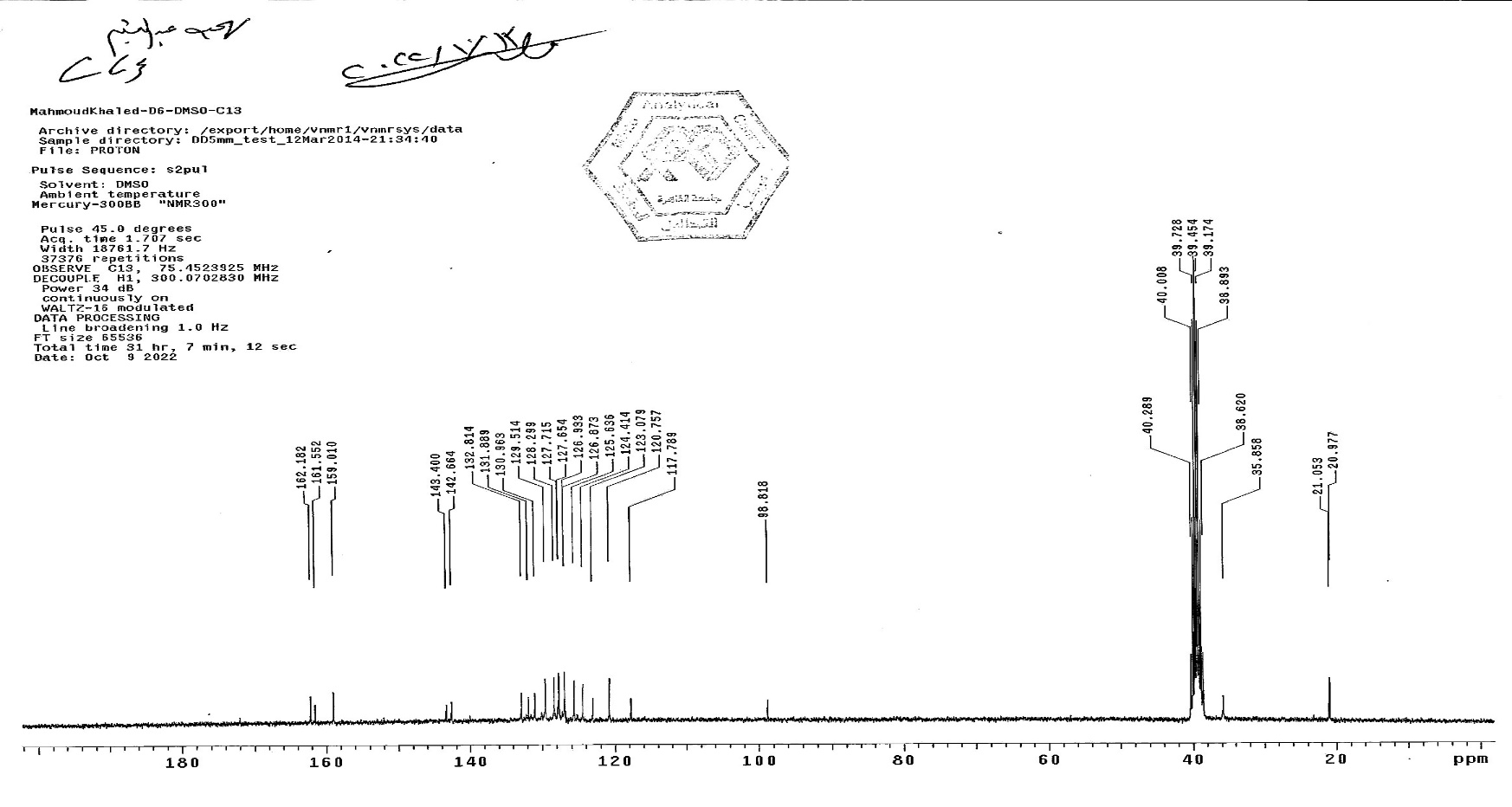

**^13^C-NMR spectrum (DMSO-d_6_) of Compound (2)**


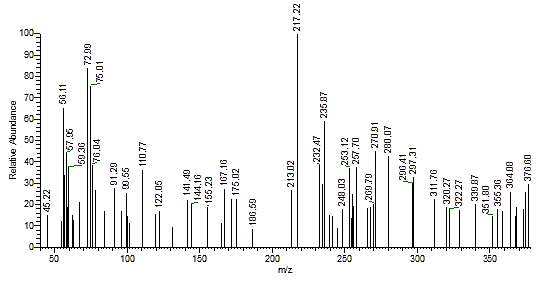

**Mass spectrum of Compound (2)**


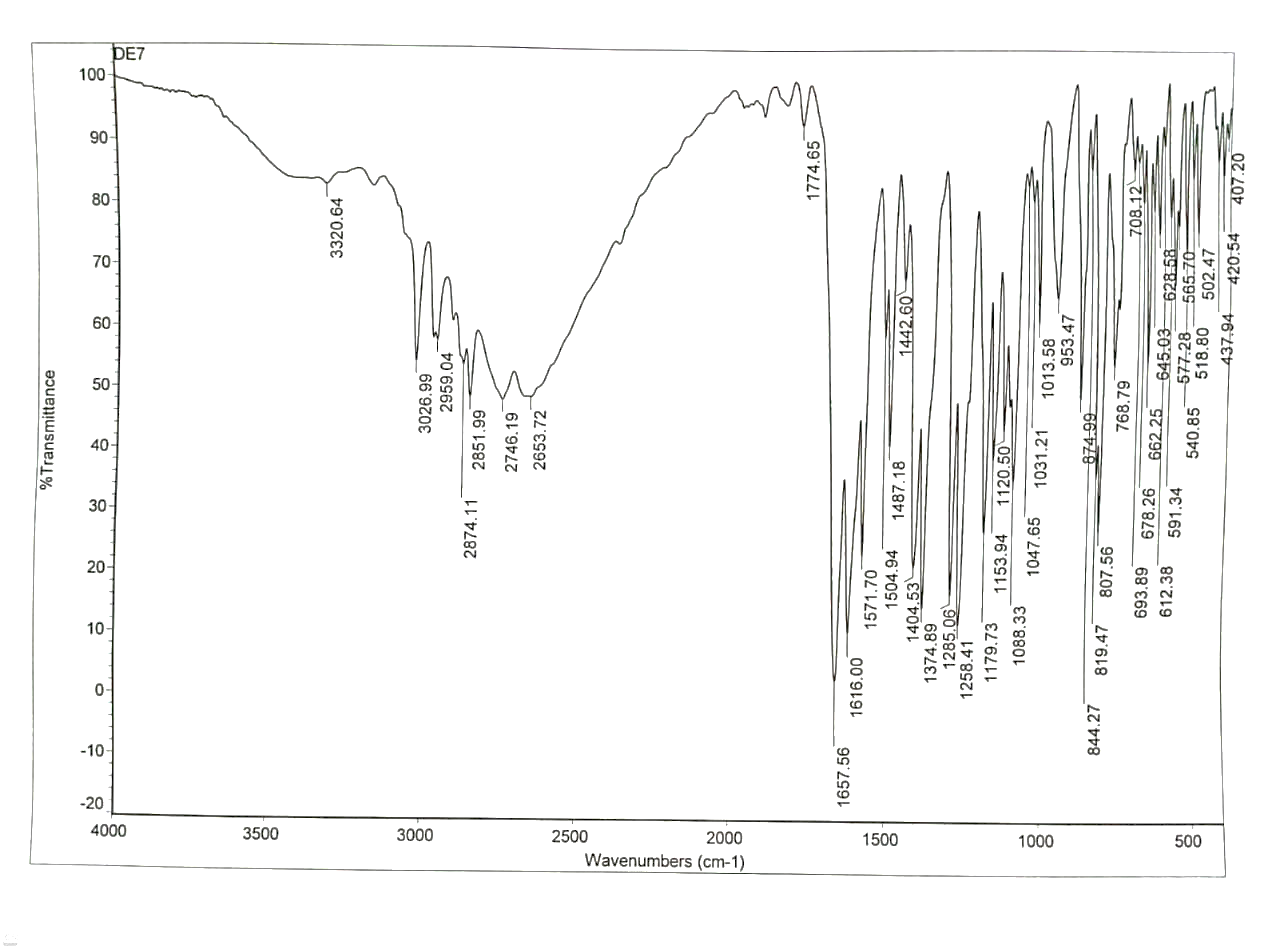

**IR spectrum of compound (3)**


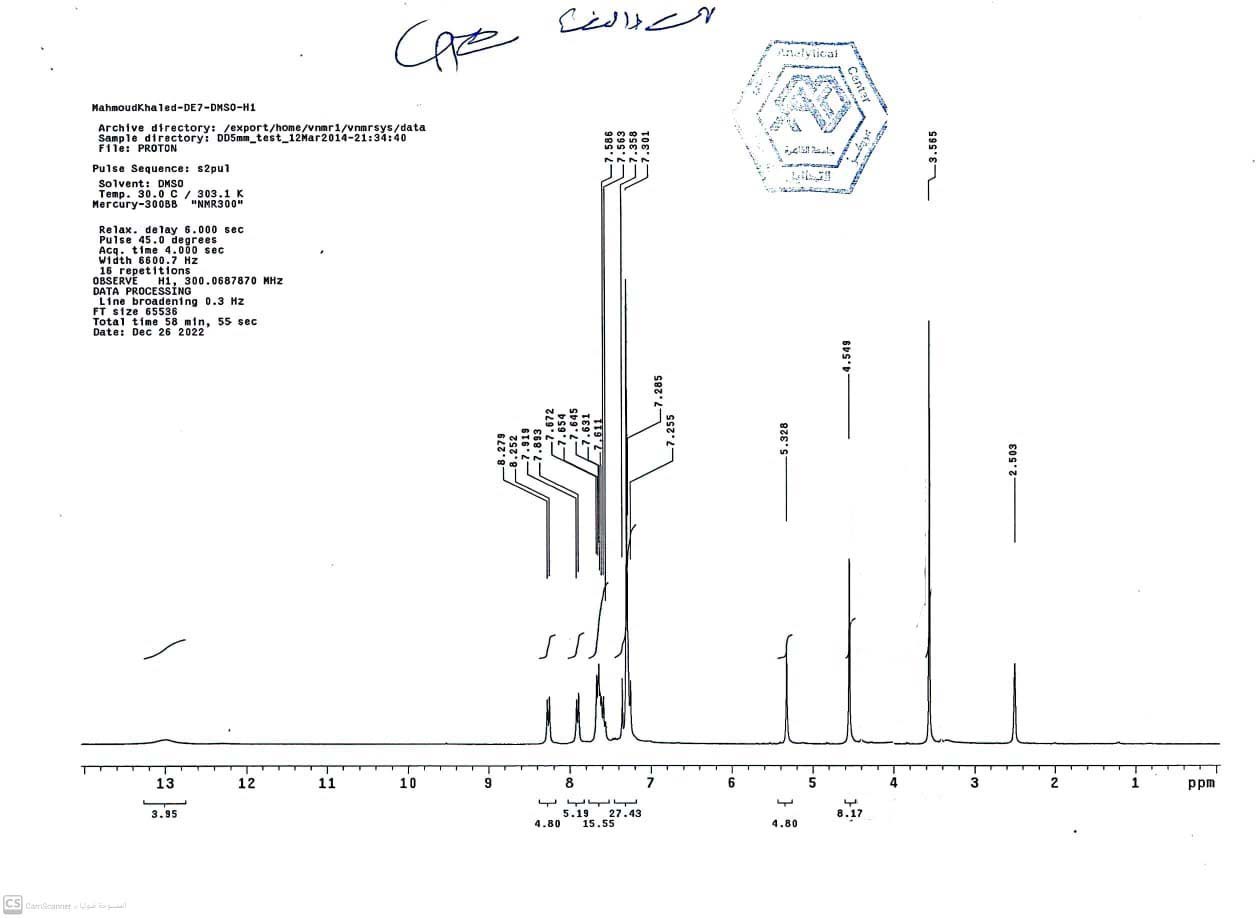

**^1^H-NMR (DMSO-d_6_) of Compound (3)**

**
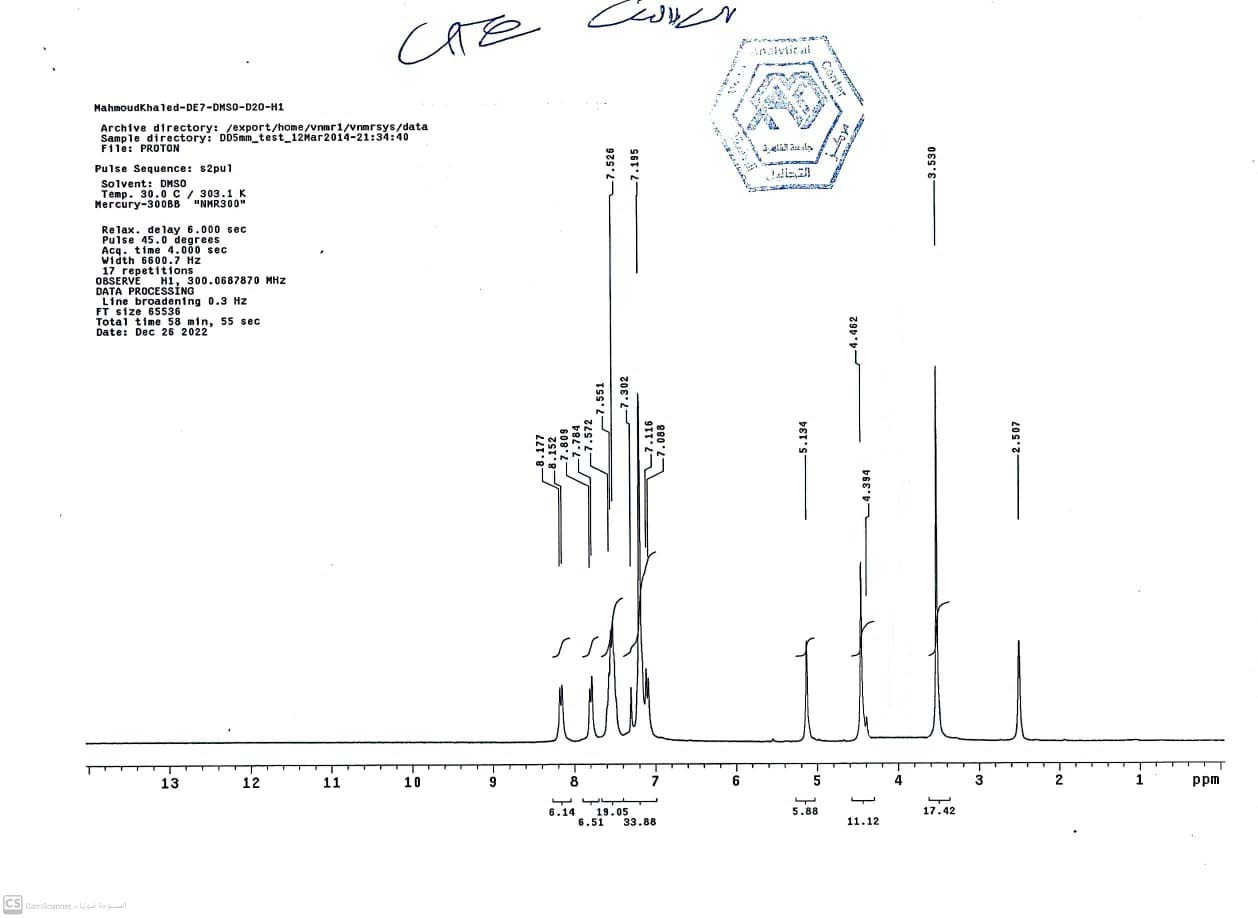
**

**^1^H-NMR spectrum (DMSO-d_6_ + D_2_O) of Compound (3)**


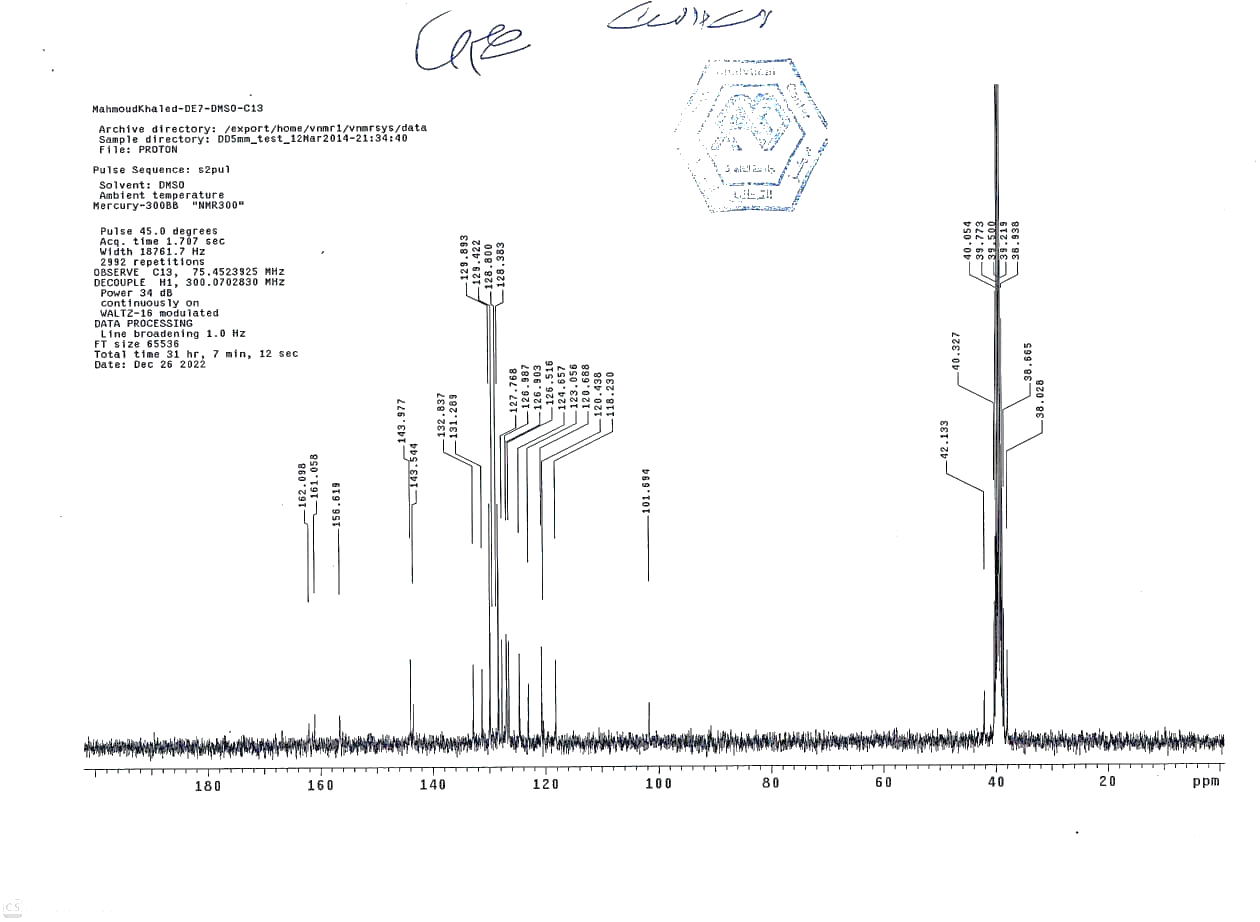

**^13^C-NMR spectrum (DMSO-d_6_) of Compound (3)**

**
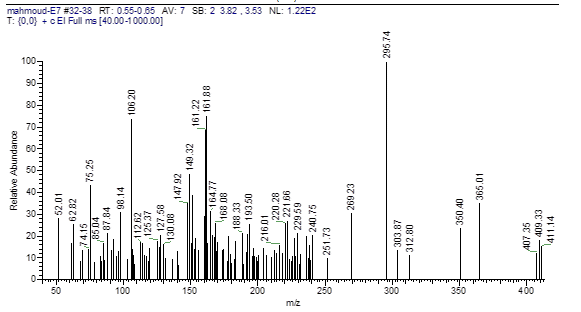
**

**Mass spectrum of Compound (3)**


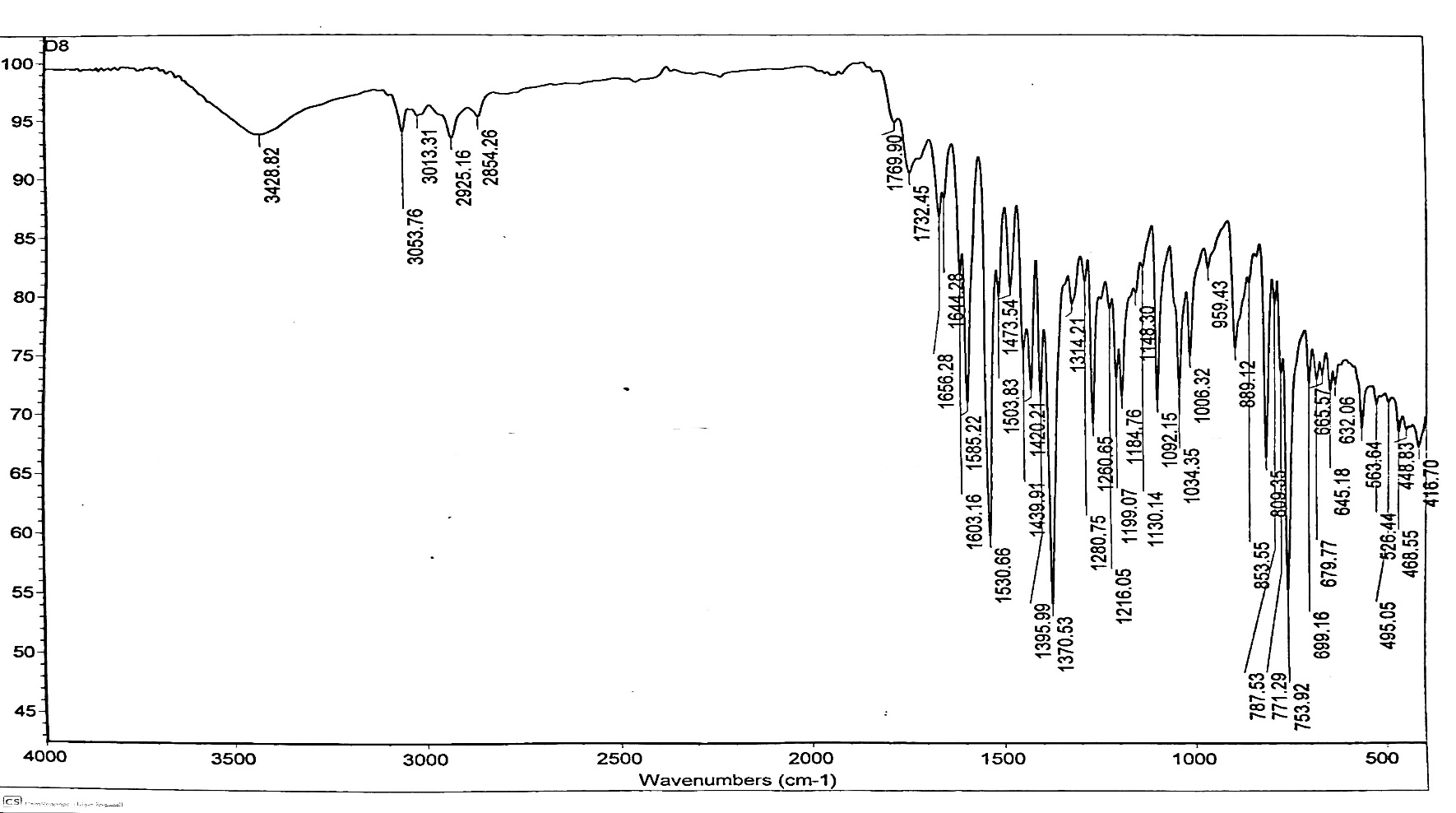

**IR spectrum of compound (4)**


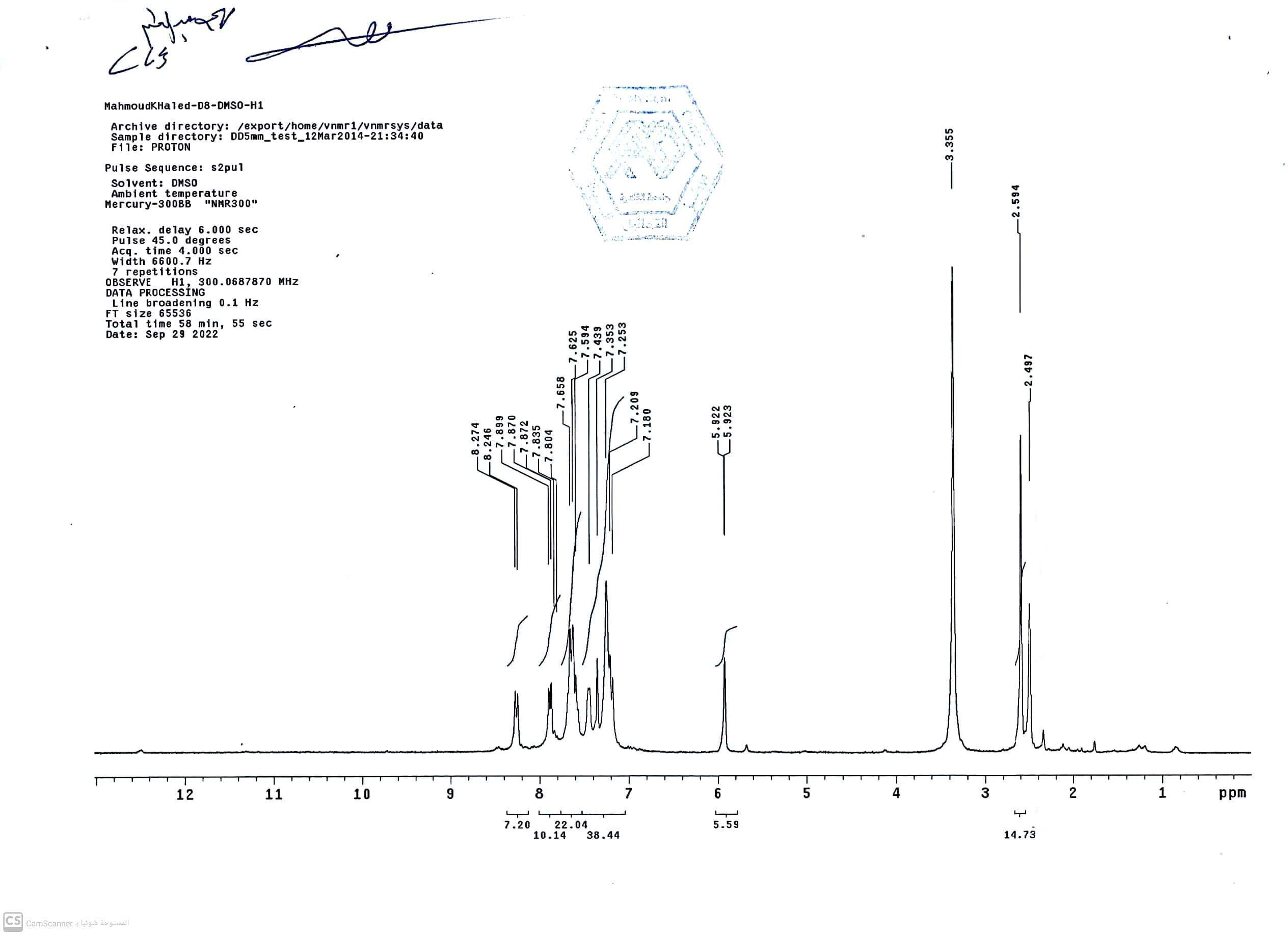

**^1^H-NMR spectrum (DMSO-d_6_) of Compound (4)**


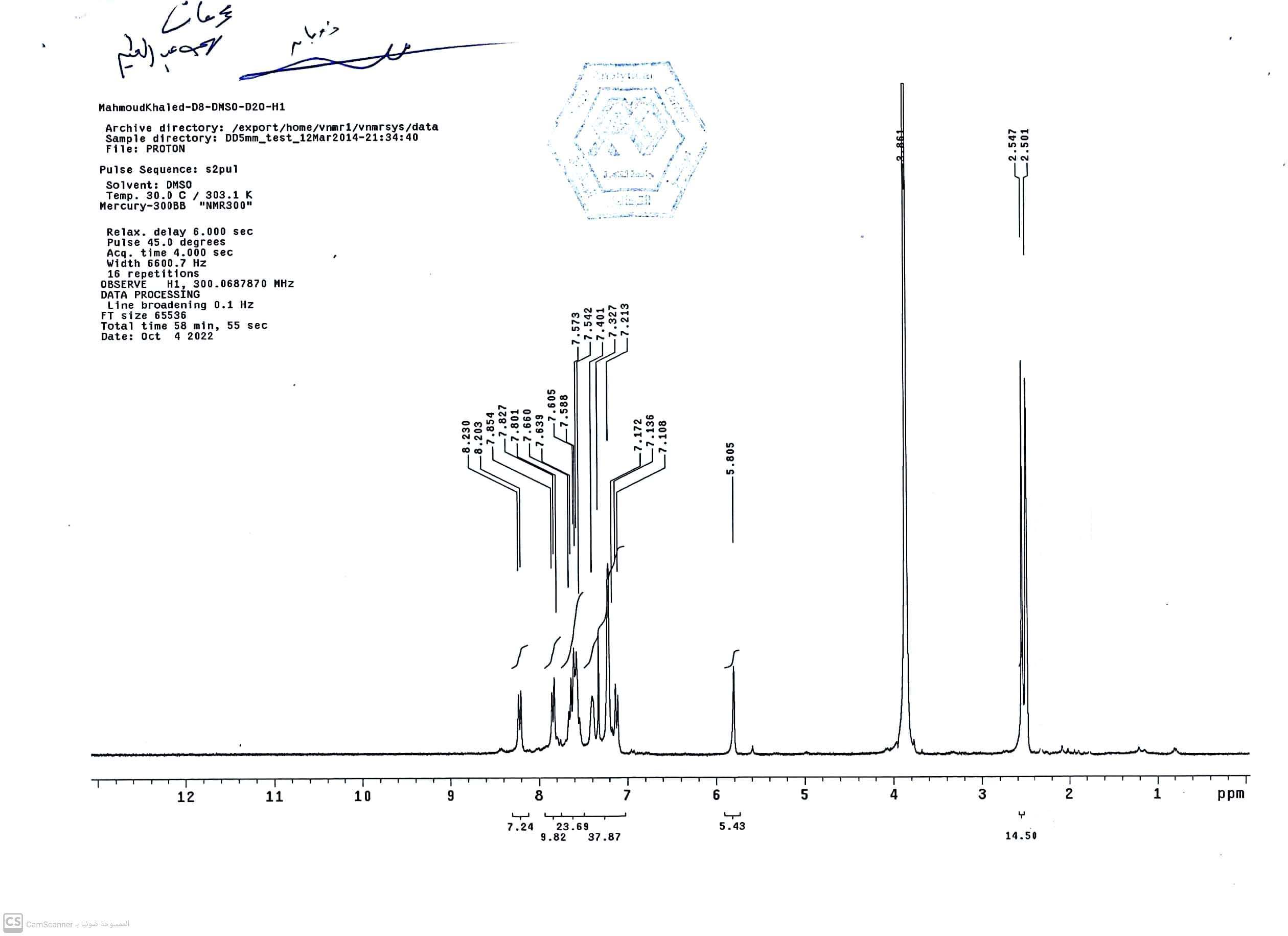

**^1^H-NMR spectrum (DMSO-d_6_ + D_2_O) of Compound (4)**


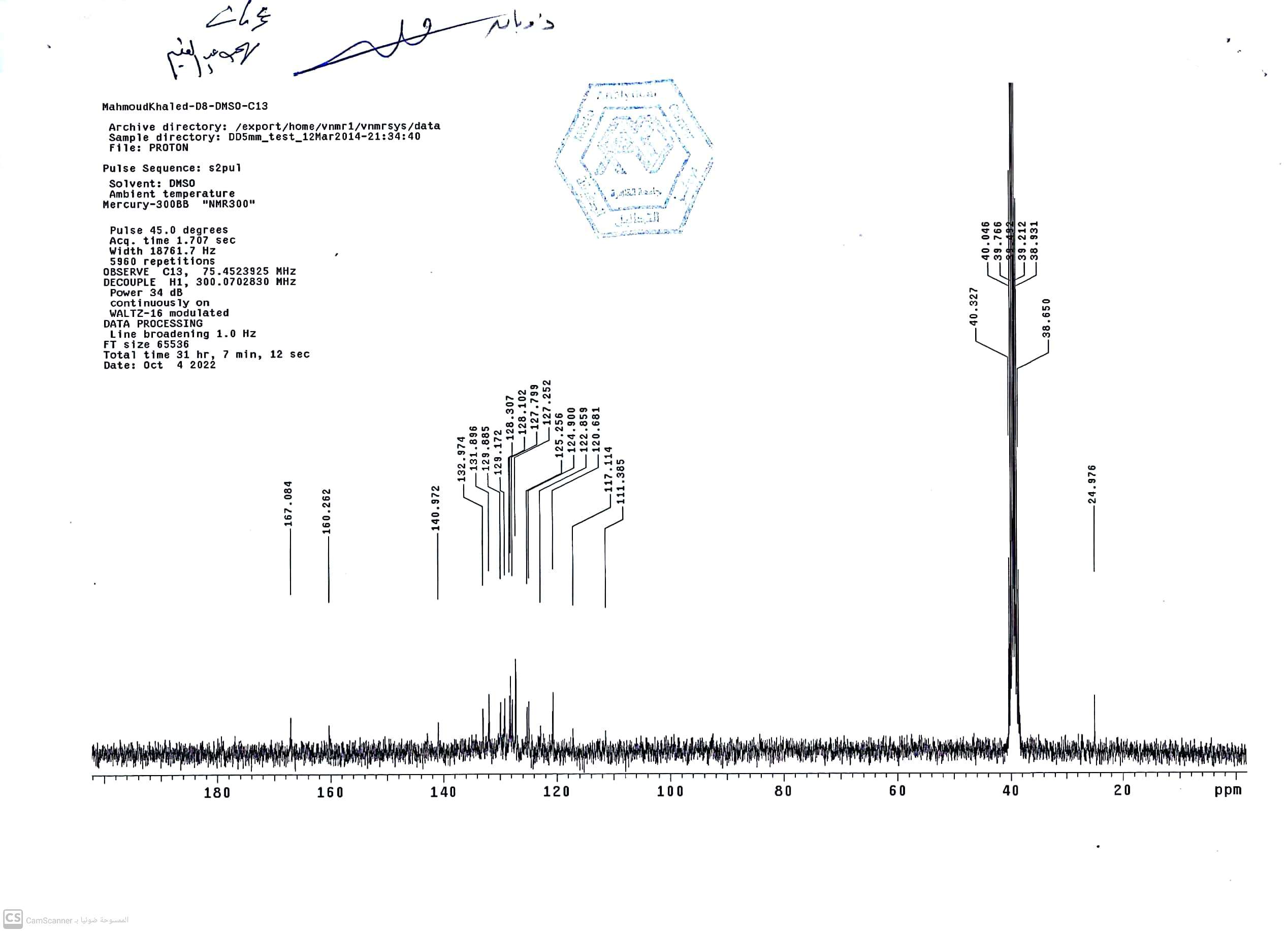

**^13^C-NMR spectrum (DMSO-d_6_) of Compound (4)**


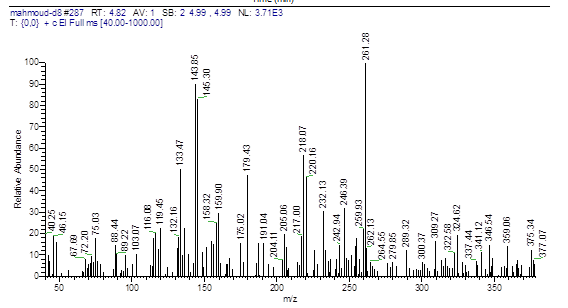

**Mass spectrum of Compound (4)**


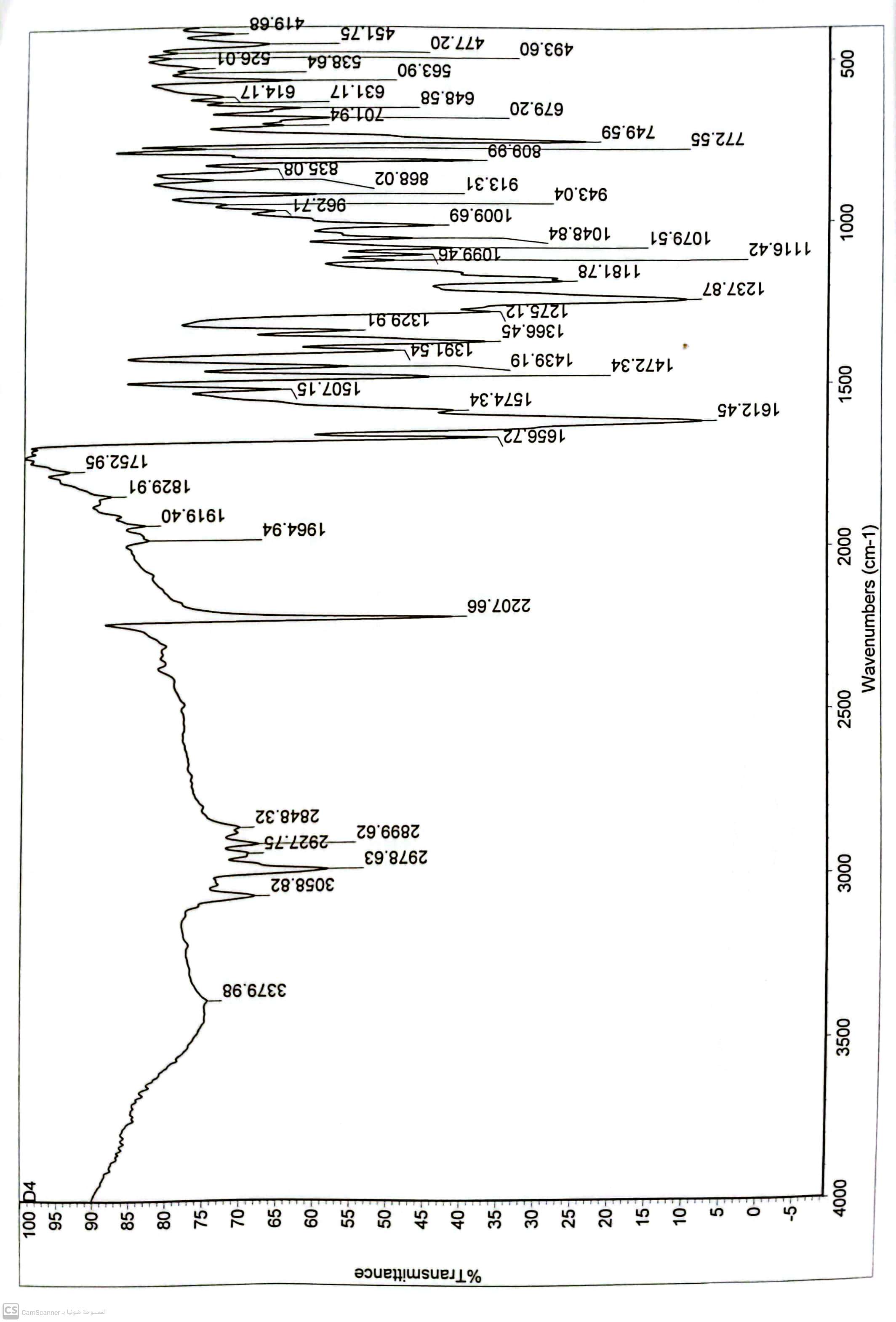

**IR spectrum of compound (5a)**

**
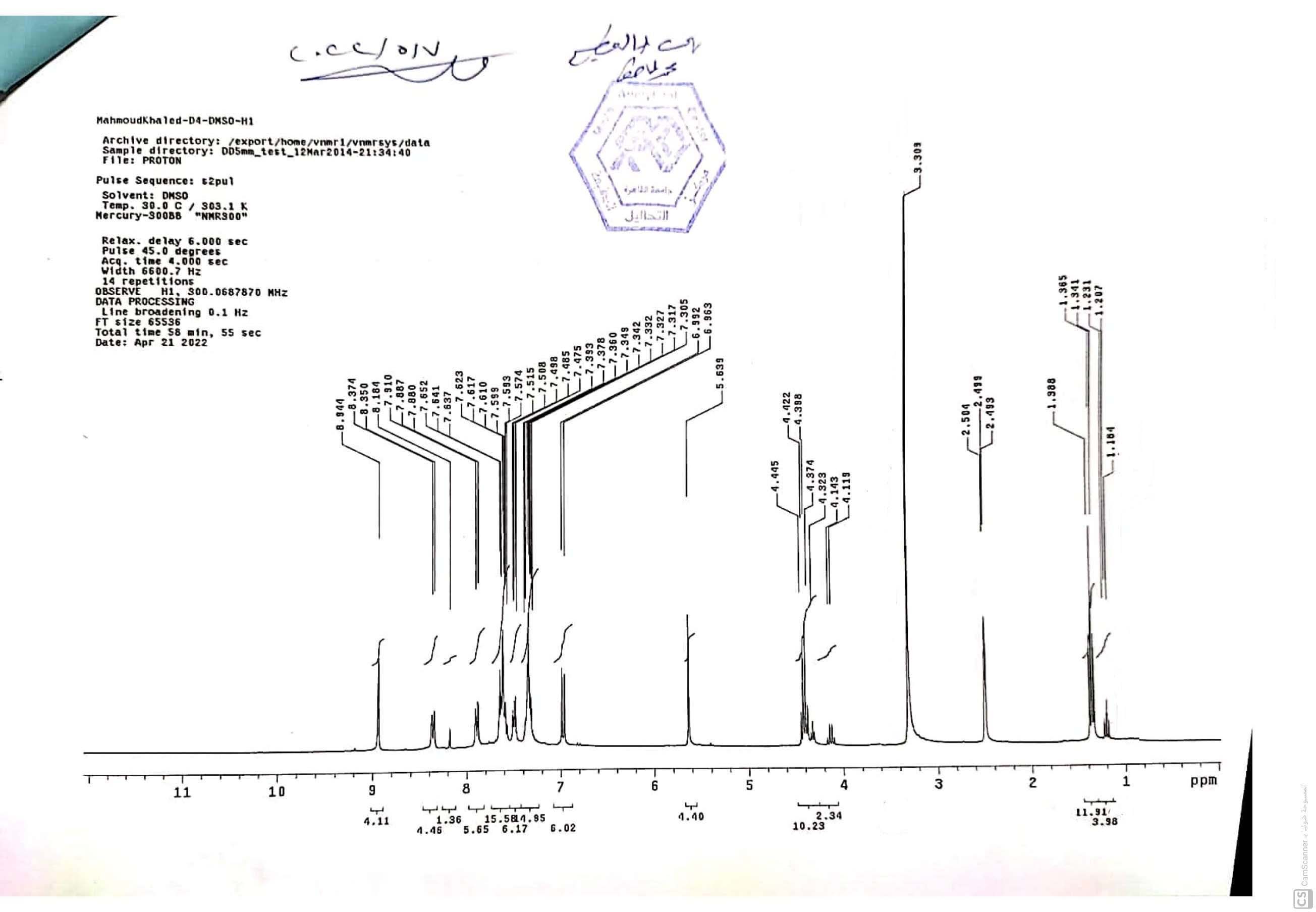
**

**^1^H-NMR spectrum (DMSO-d_6_) of Compound (5a)**


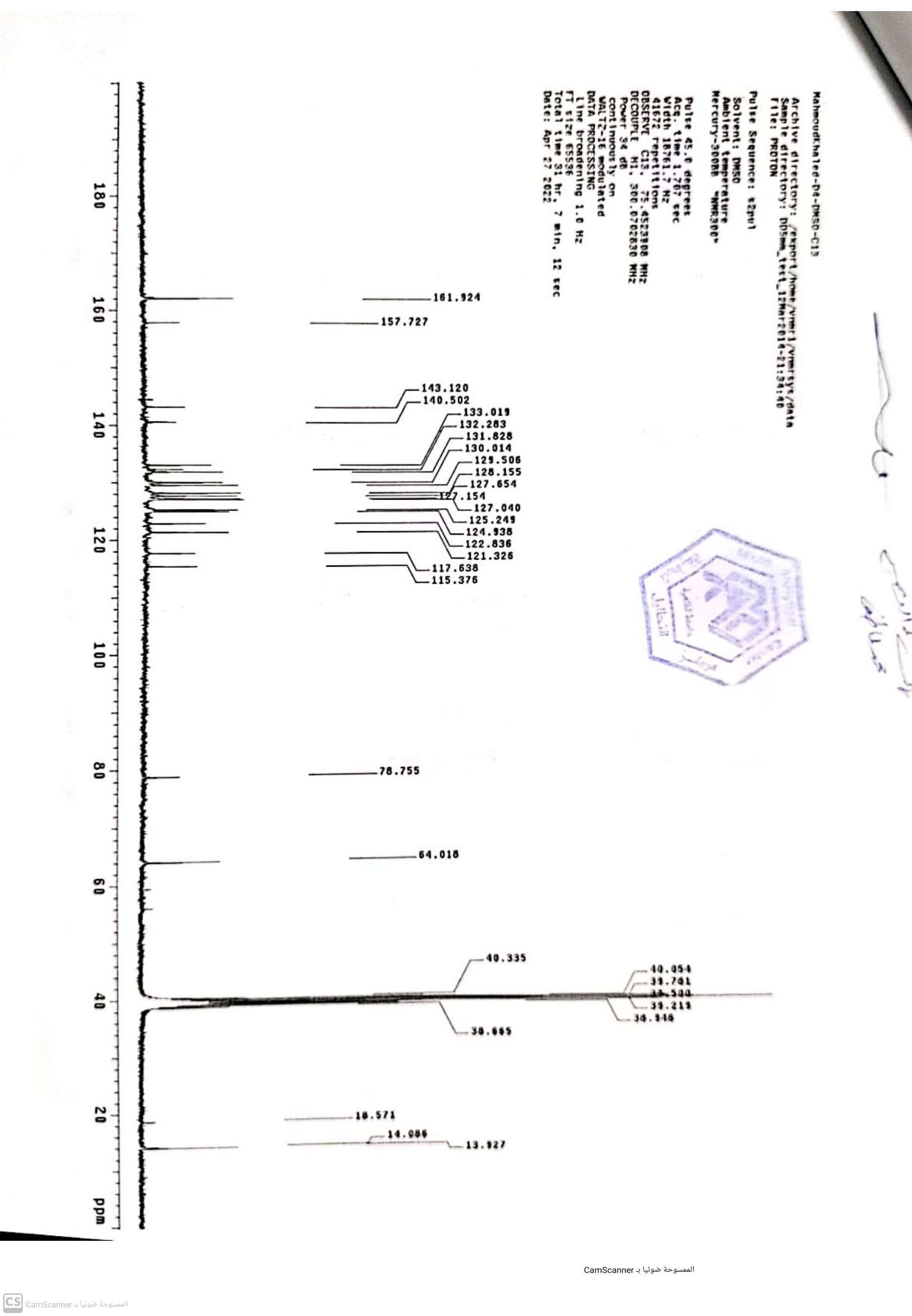

**^13^C-NMR spectrum (DMSO-d_6_) of Compound (5a)**


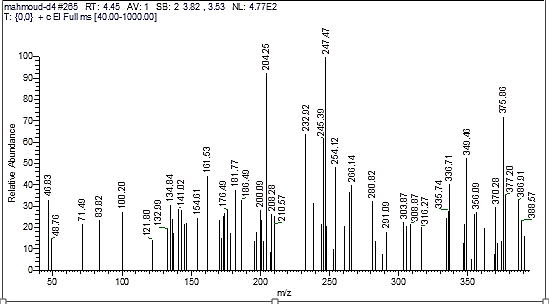

**Mass spectrum of Compound (5a)**


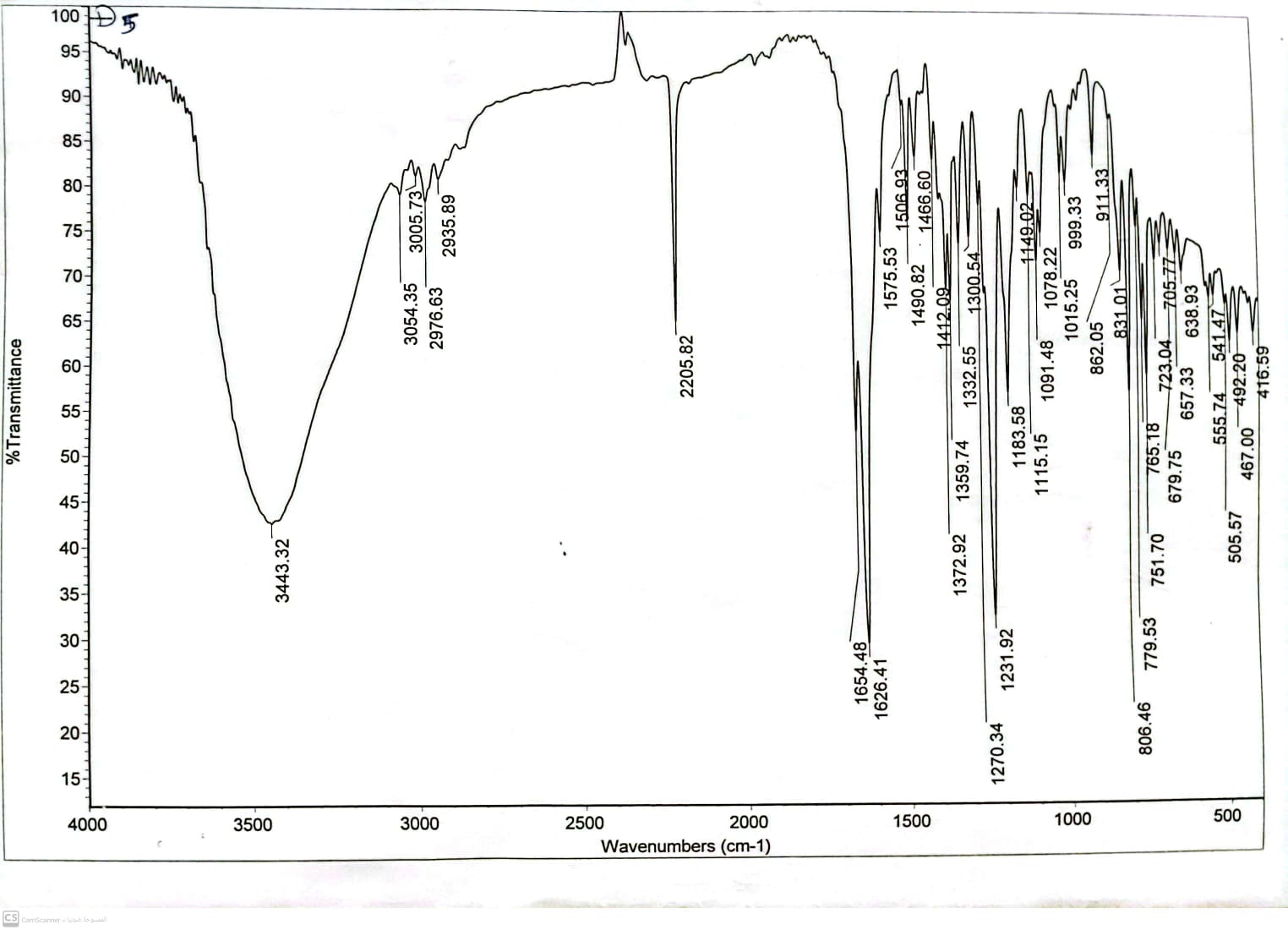

**IR spectrum of compound (5b)**

**
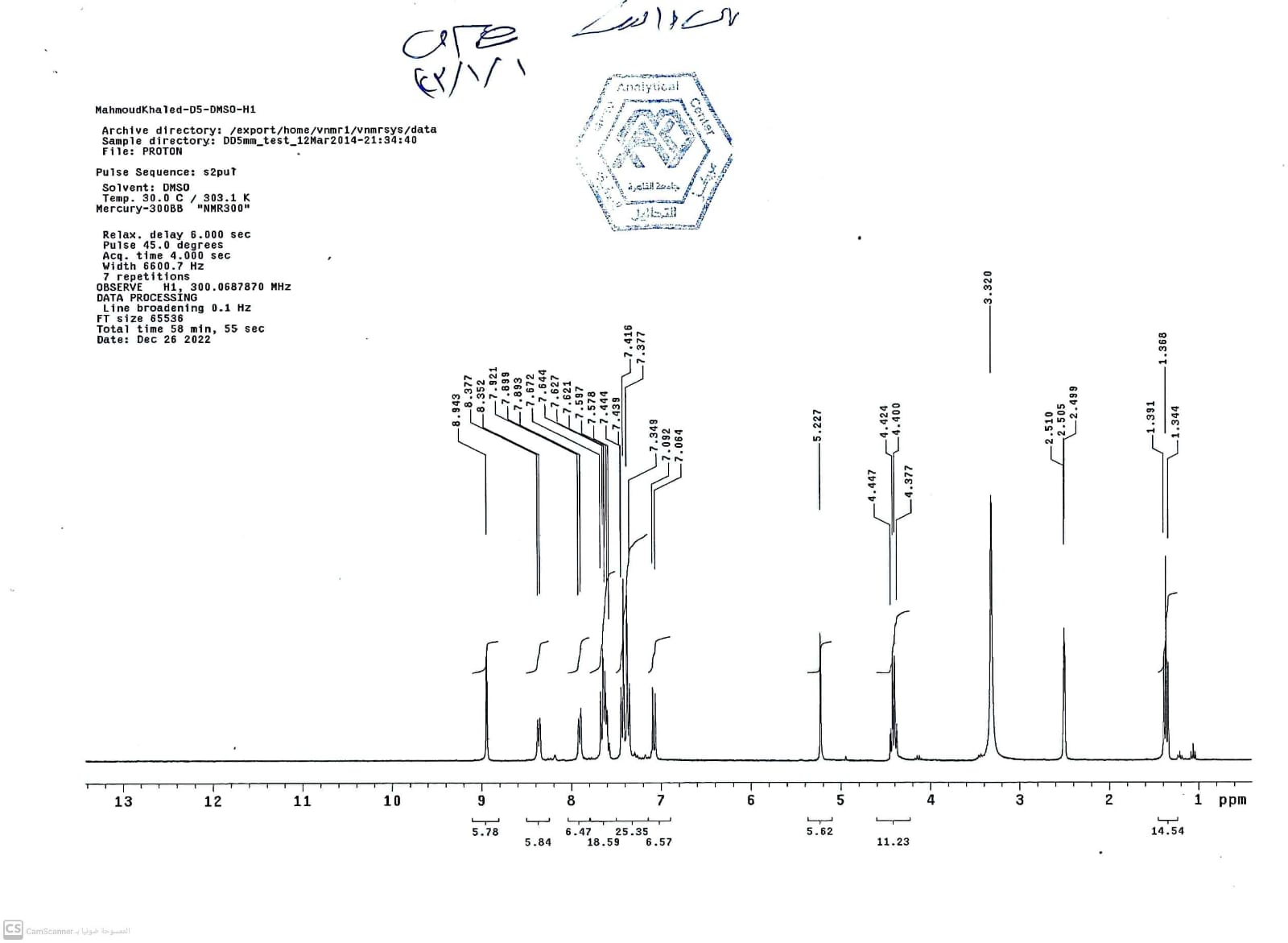
**

**^1^H-NMR (DMSO-d_6_) of Compound (5b)**

**
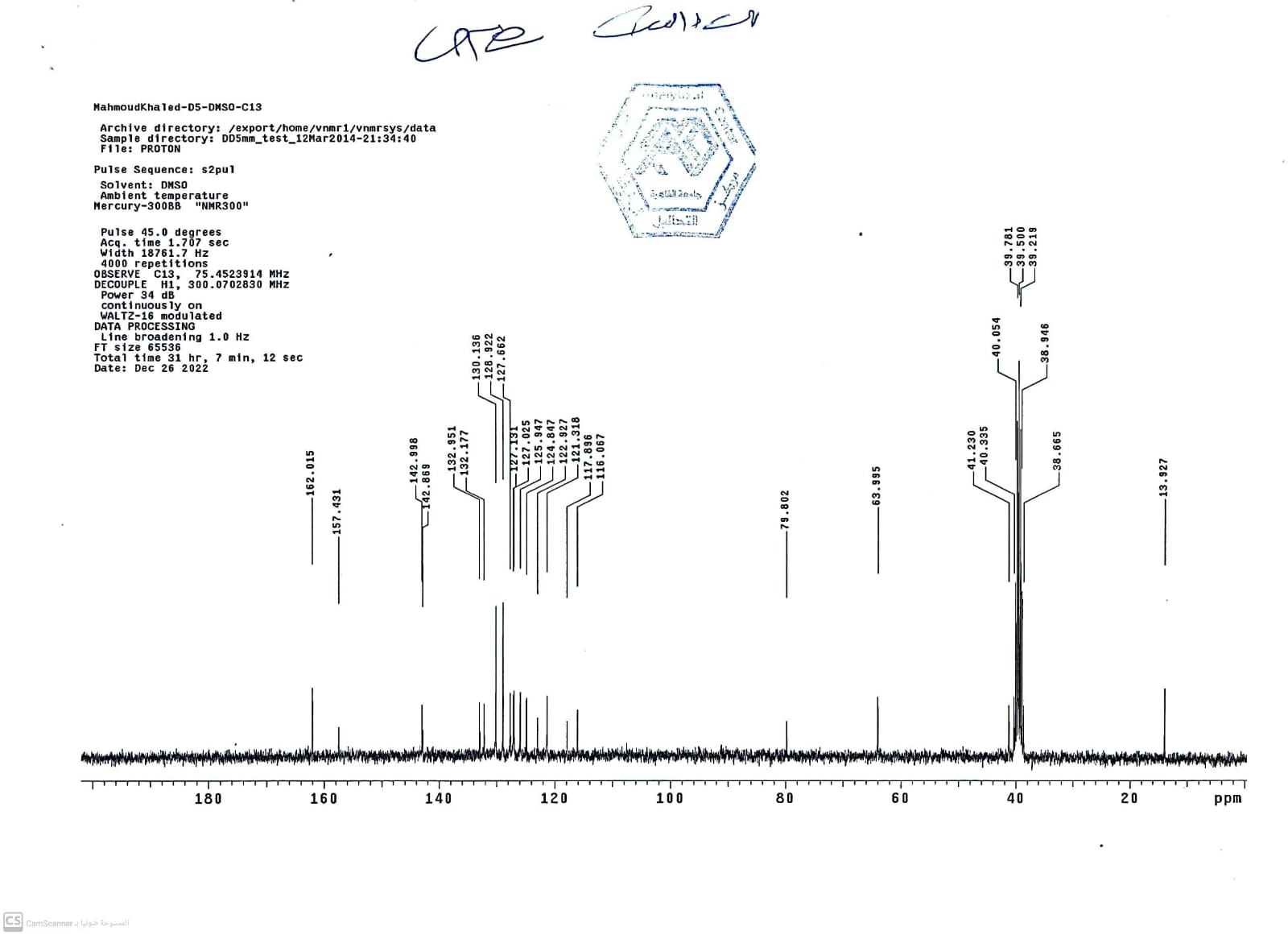
**

**^13^C-NMR spectrum (DMSO-d_6_) of Compound (5b)**


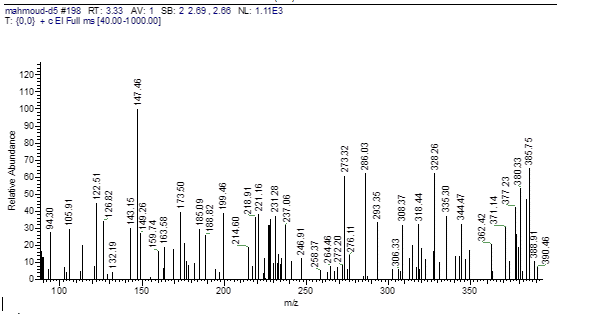

**Mass spectrum of Compound (5b)**


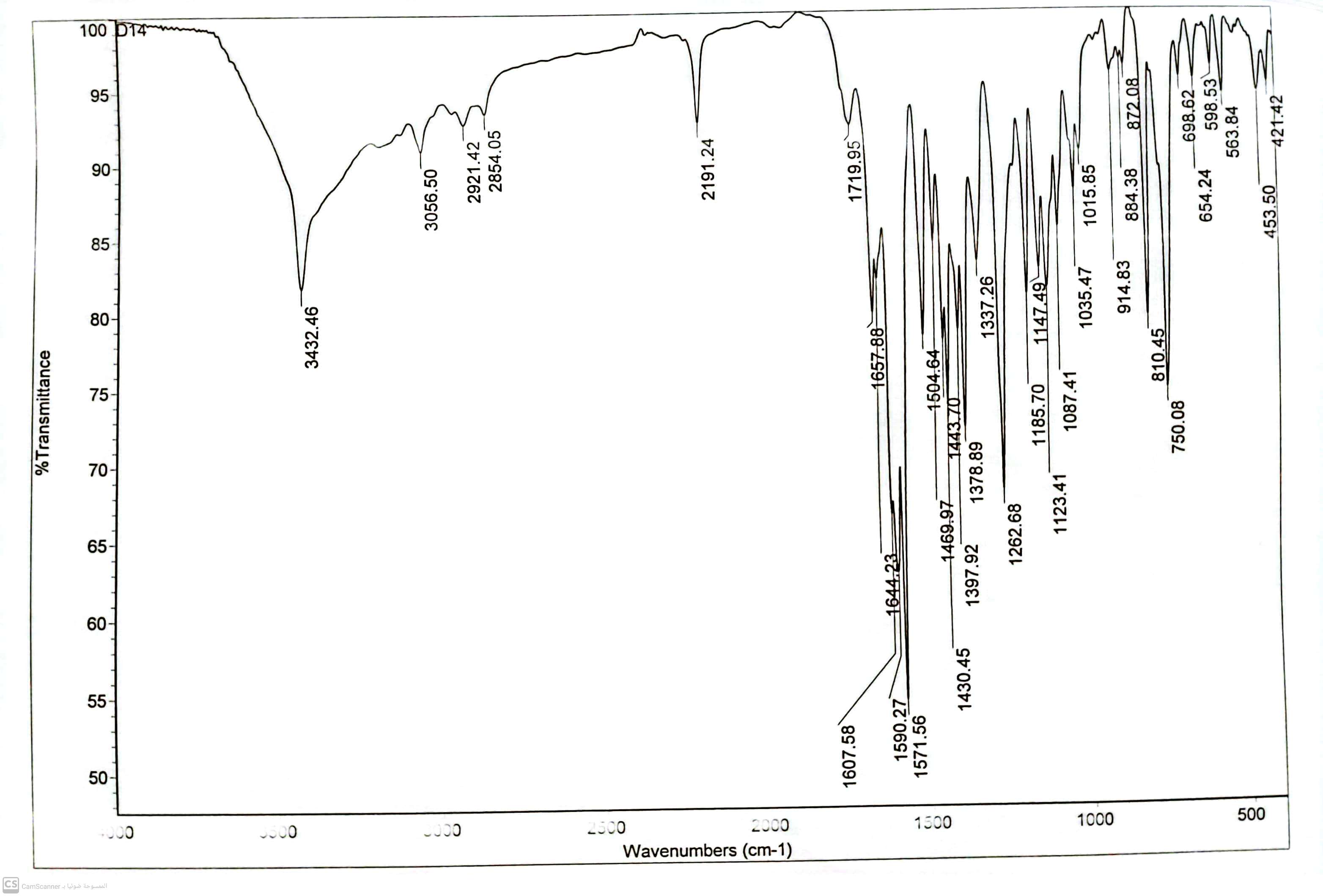

**IR spectrum of compound (6)**


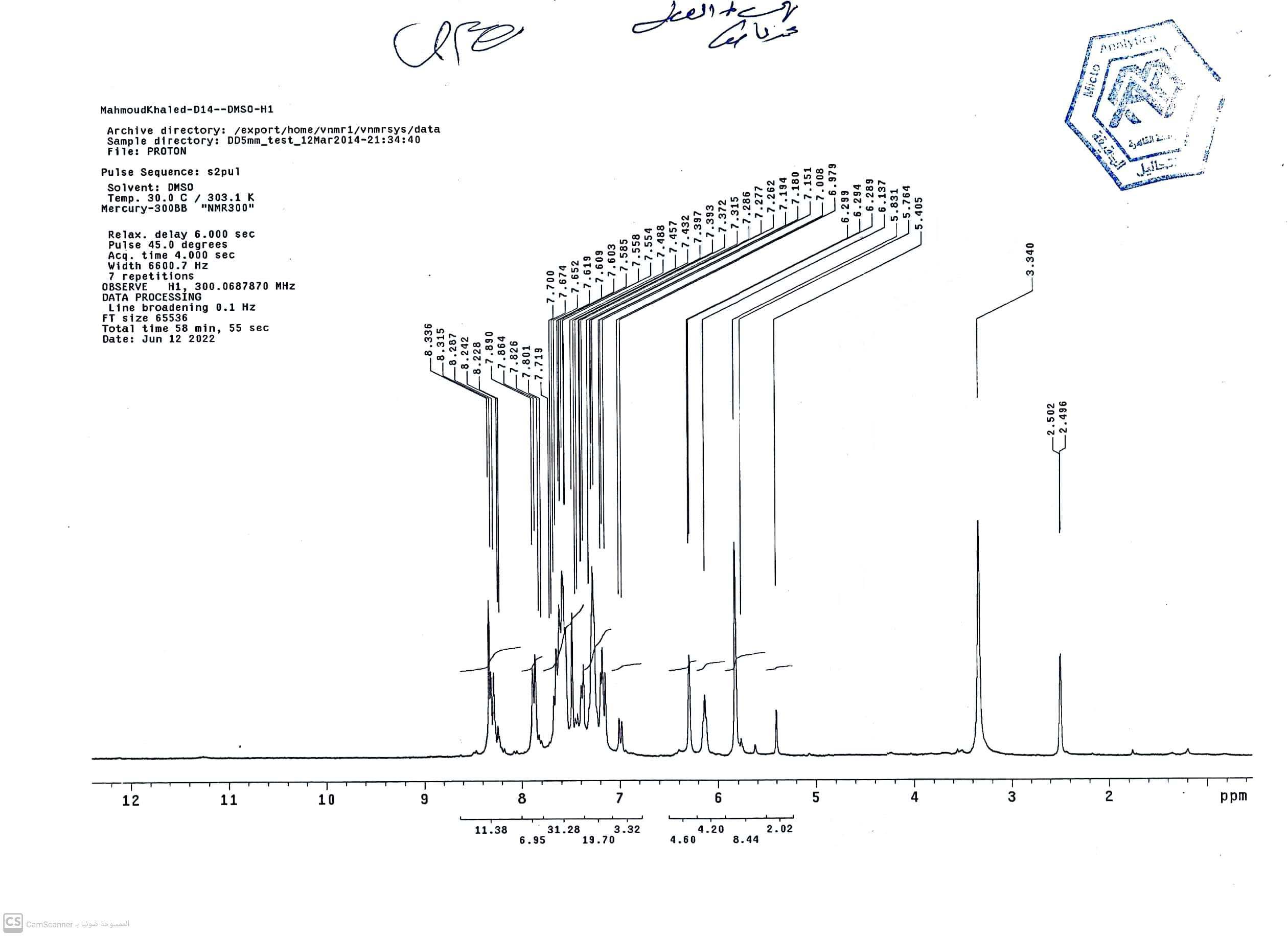

**^1^H-NMR (DMSO- d_6_) of Compound (6)**


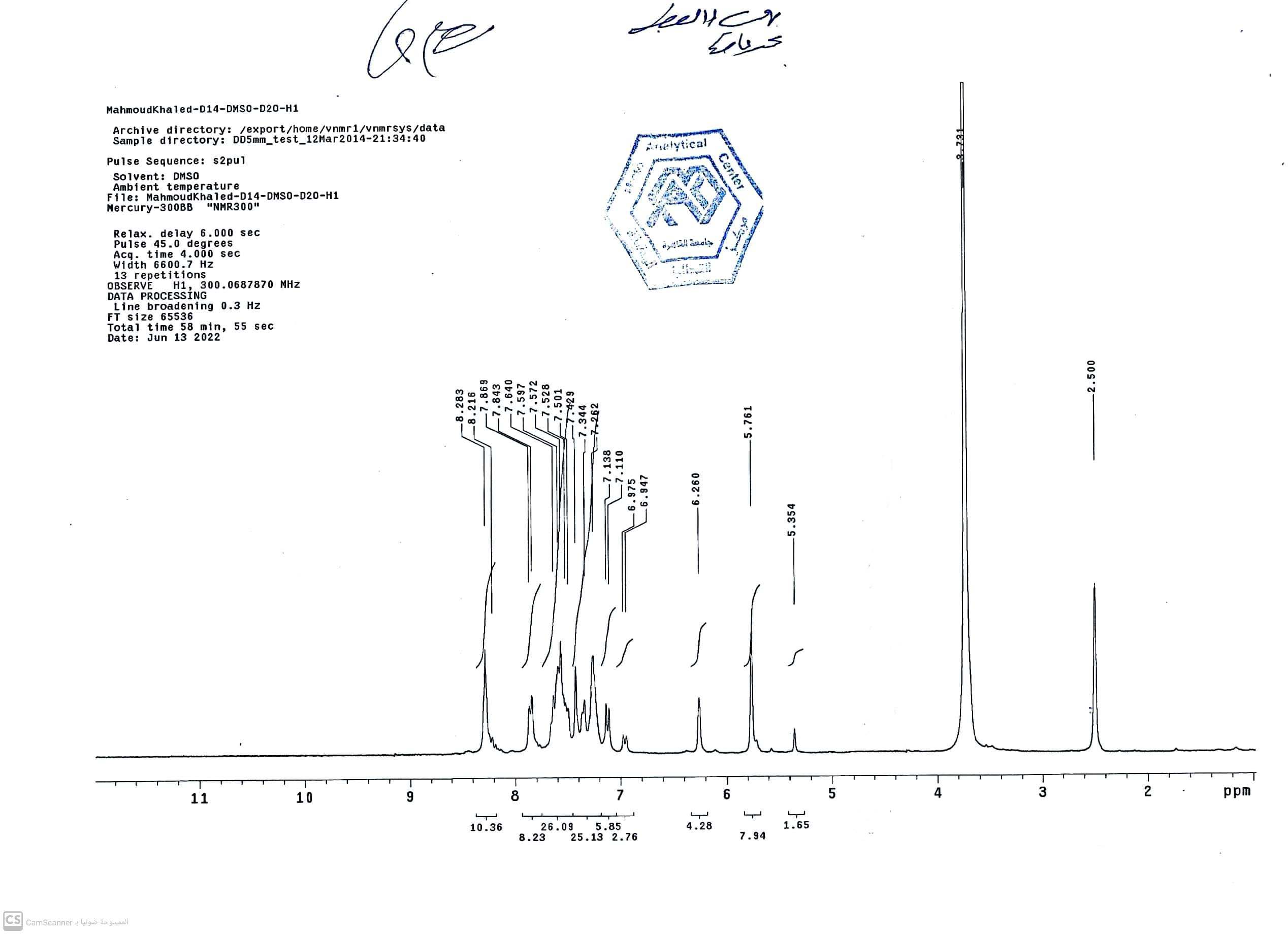

**^1^H-NMR spectrum (DMSO-d_6_ + D_2_O) of Compound (6)**


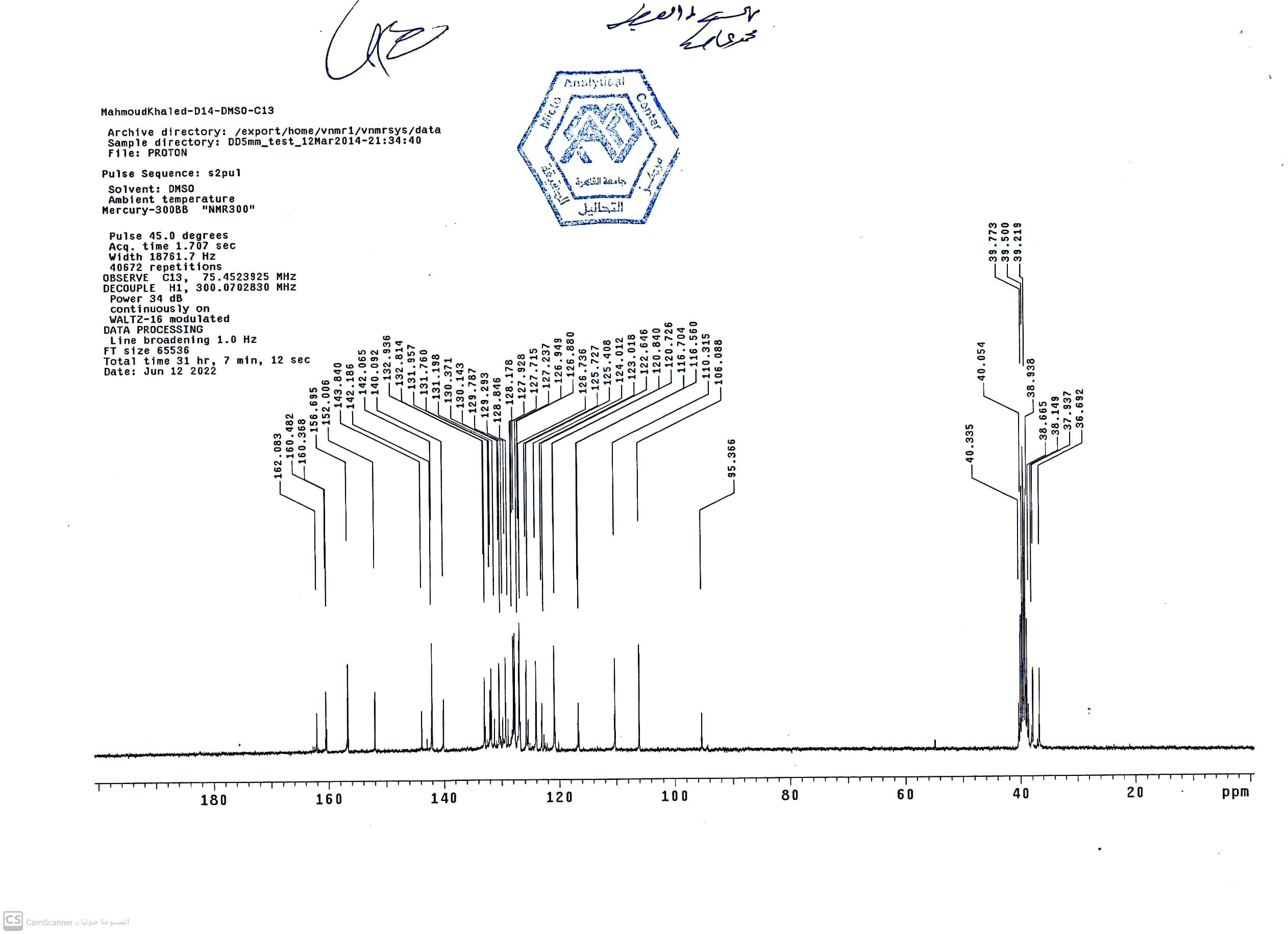


**^13^C-NMR spectrum (DMSO-d_6_) of Compound (6)**


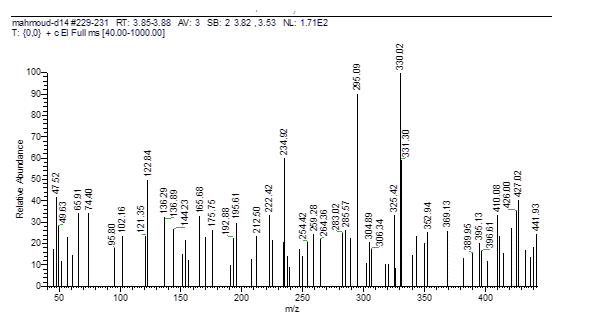

**Mass spectrum of Compound (6)**


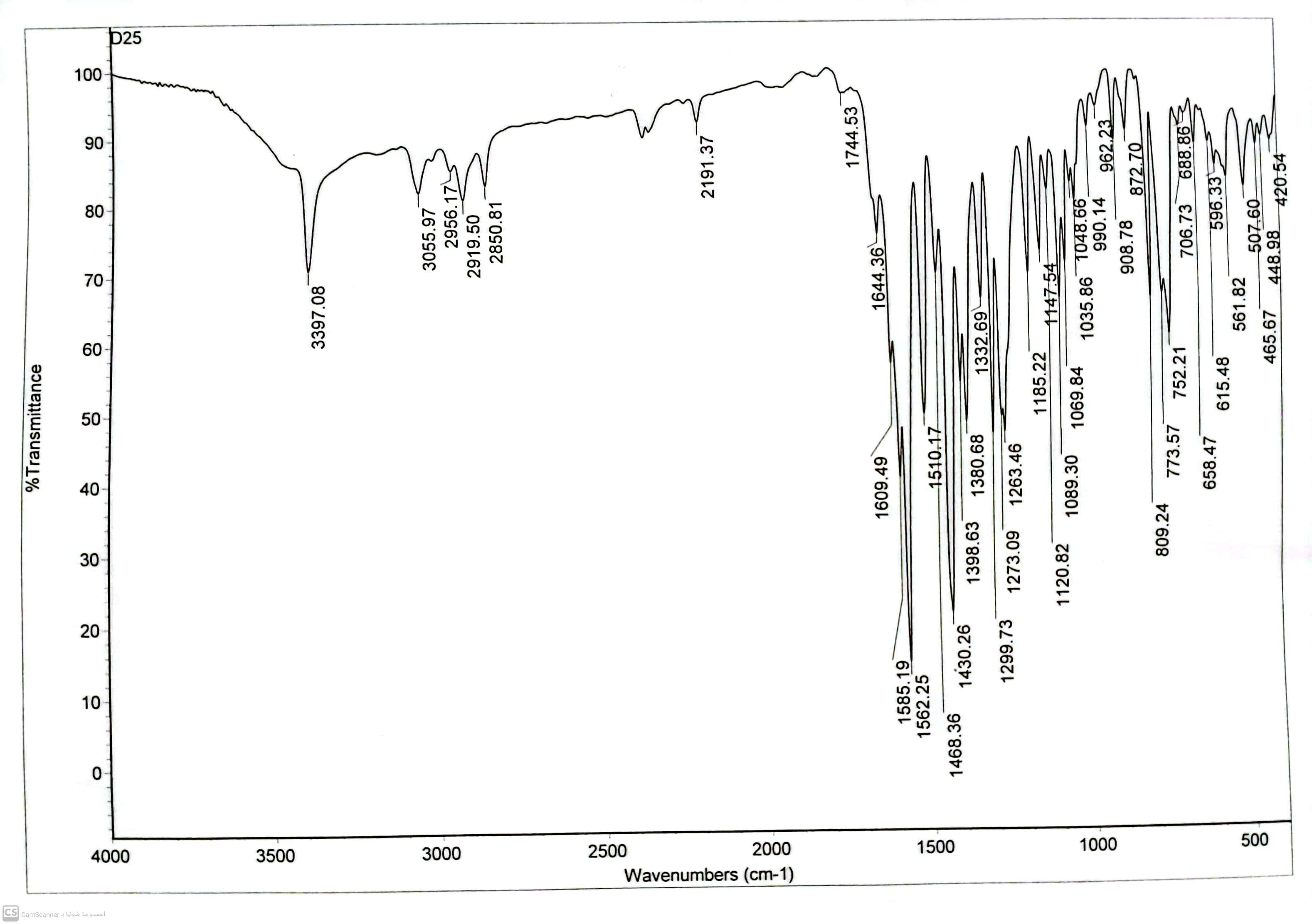

**IR spectrum of compound (7)**


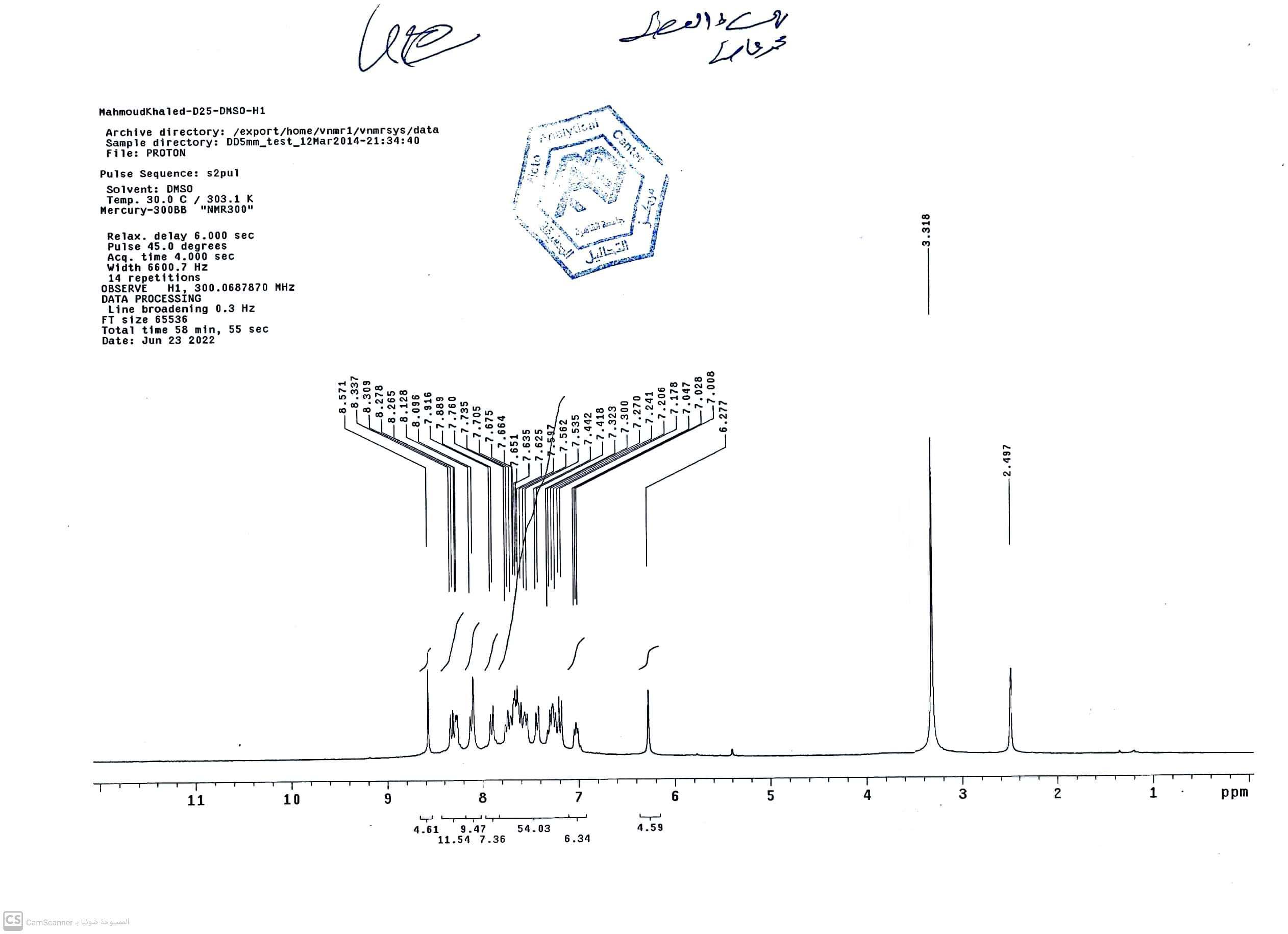

**^1^H-NMR (DMSO-d_6_) of Compound (7)**


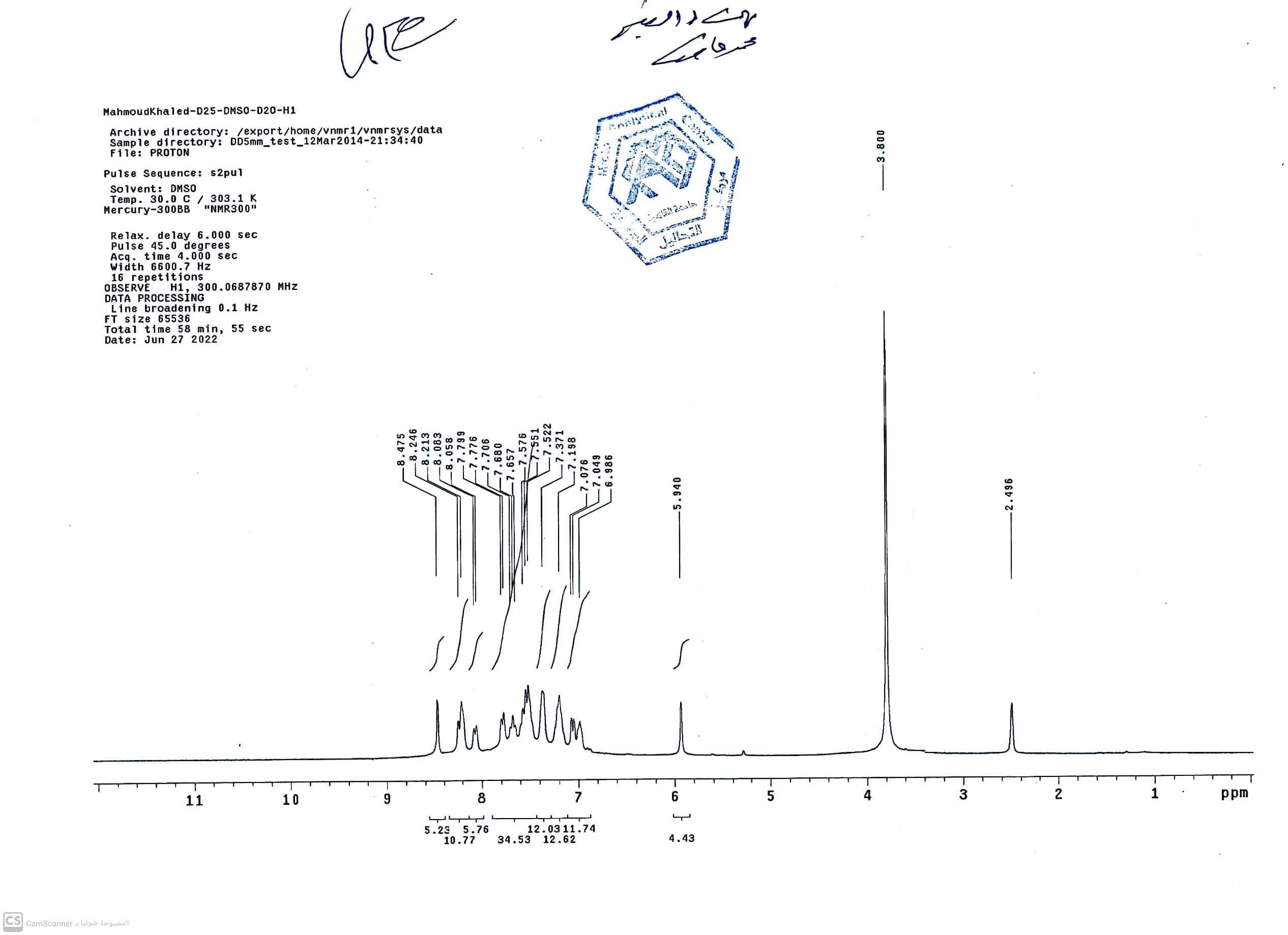

**^1^H-NMR spectrum (DMSO-d_6_ + D_2_O) of Compound (7)**


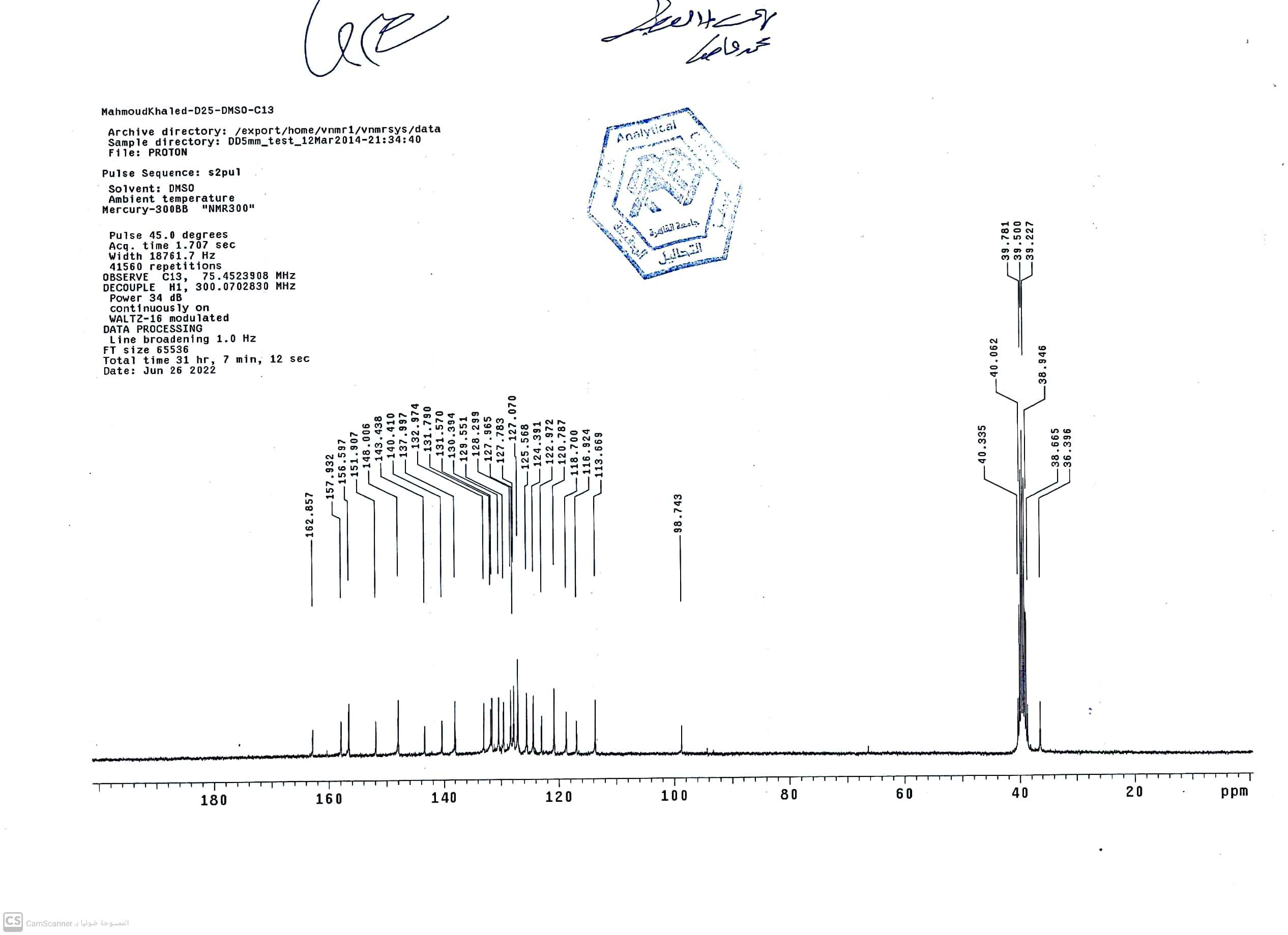

**^13^C-NMR spectrum (DMSO- d_6_) of Compound (7)**


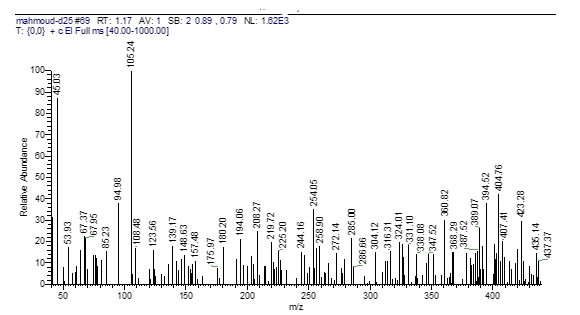

**Mass spectrum of Compound (7)**


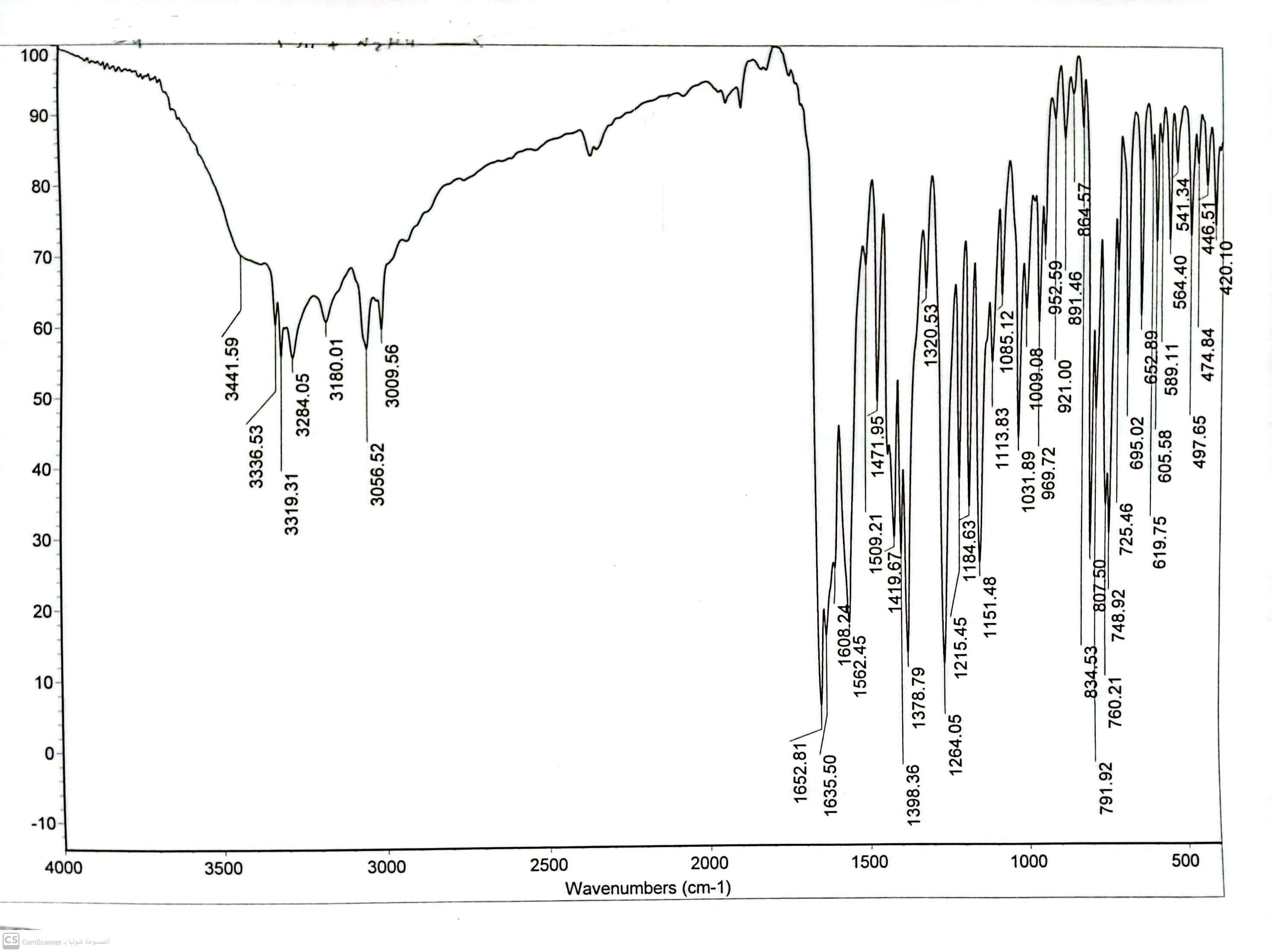

**IR spectrum of compound (8)**


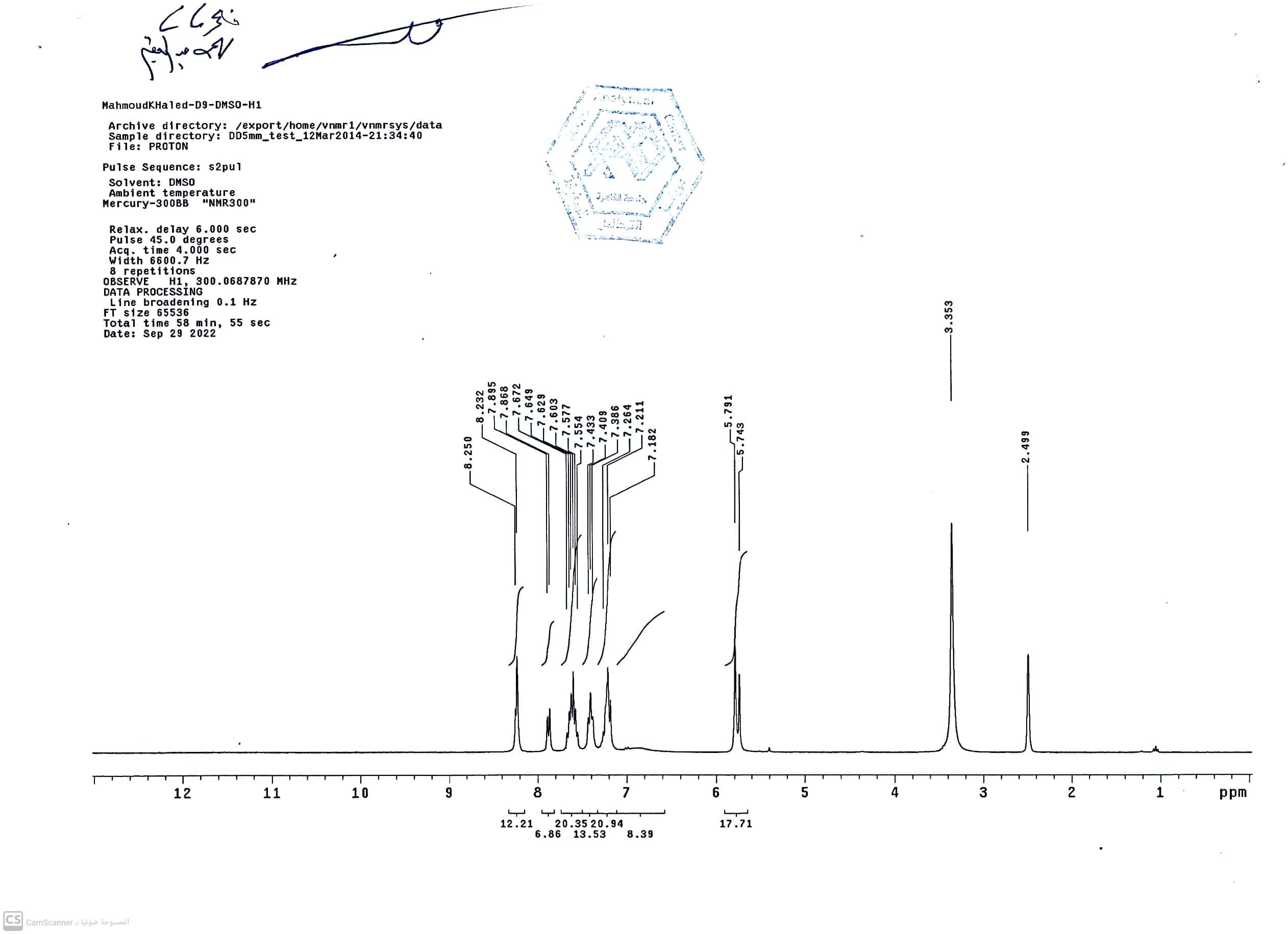

**^1^H-NMR (DMSO- d_6_) of Compound (8)**


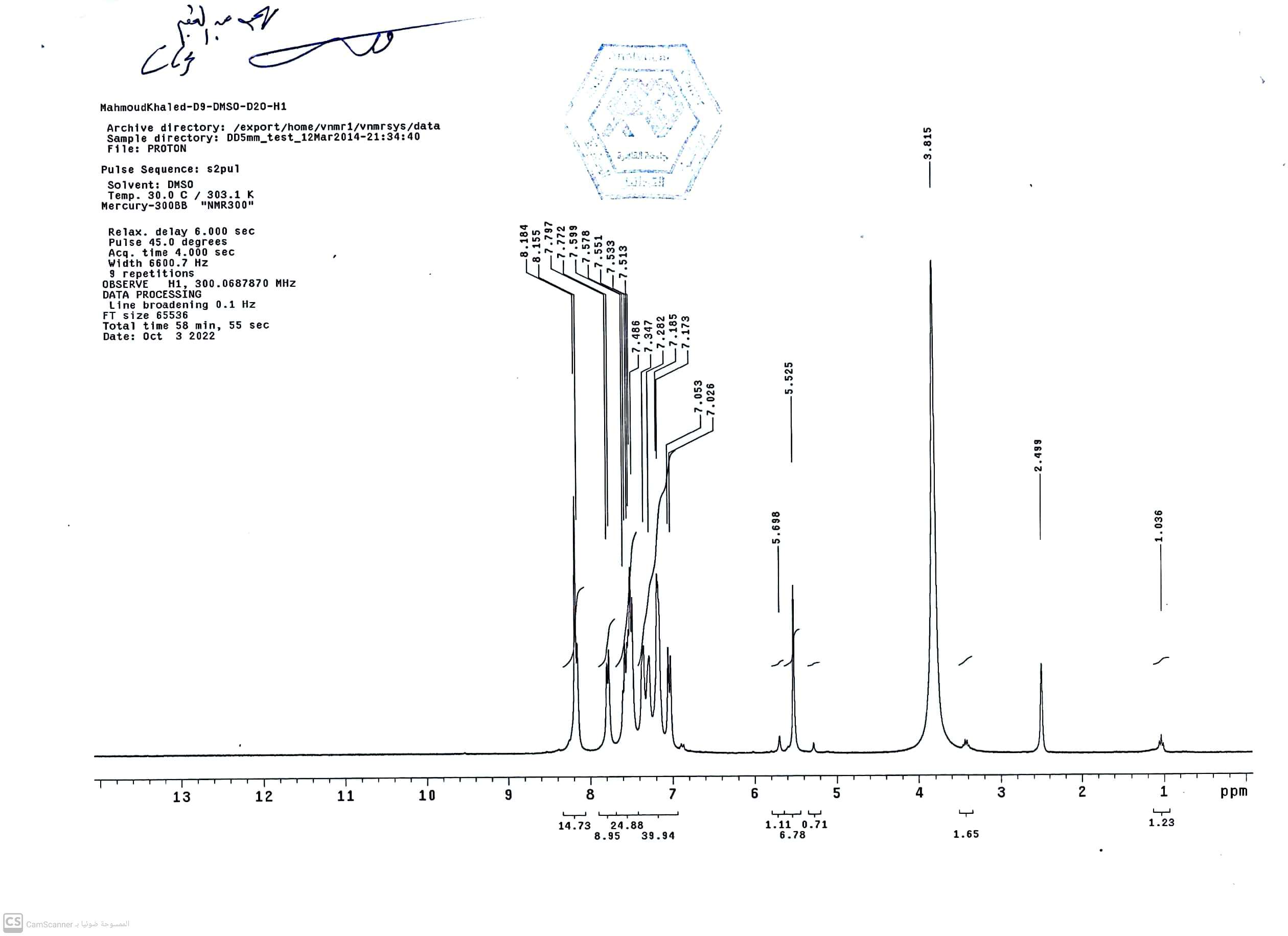

**^1^H-NMR spectrum (DMSO- d_6_ + D_2_O) of Compound (8)**


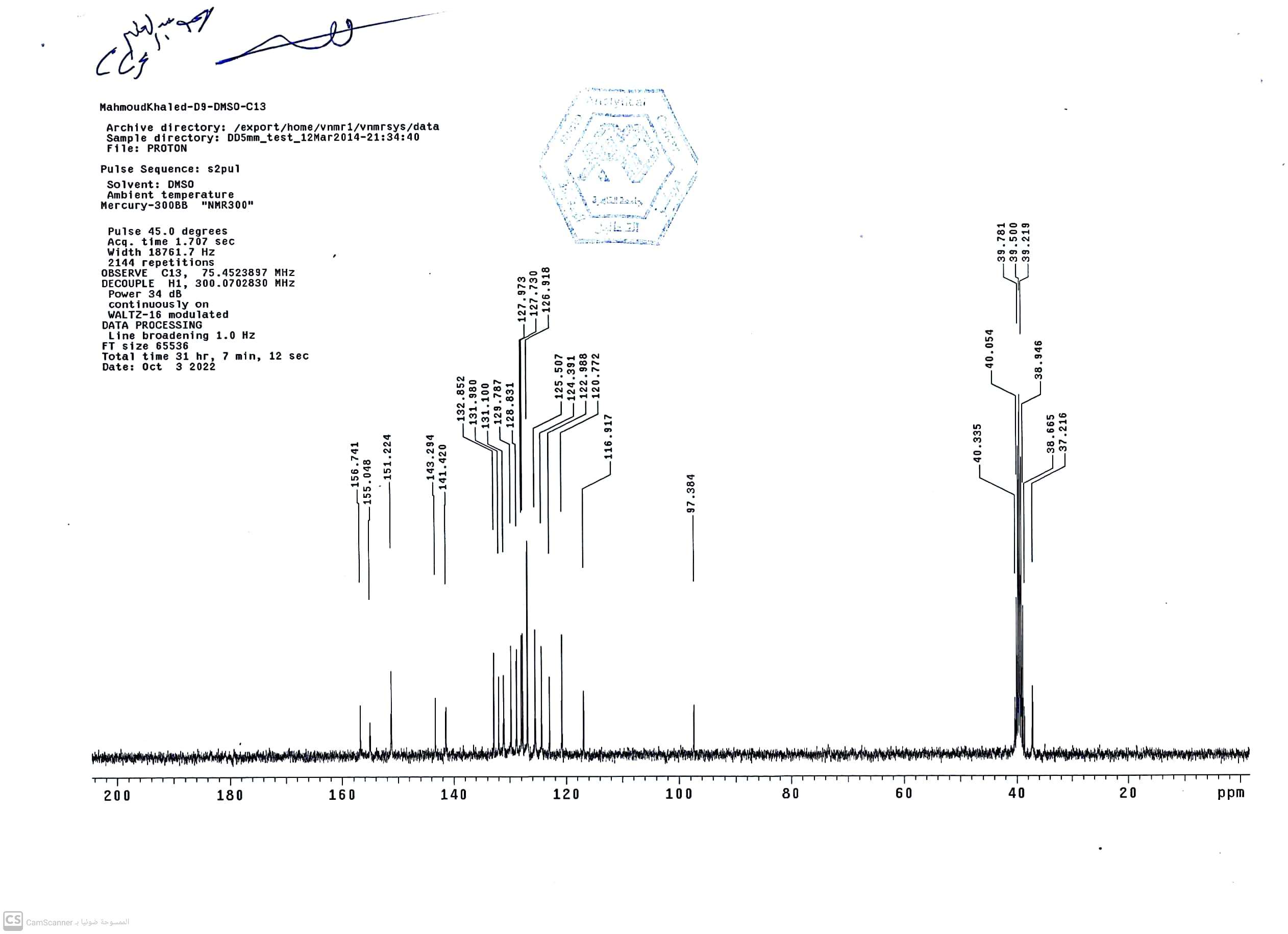

**^13^C-NMR spectrum (DMSO-d_6_) of Compound (8)**


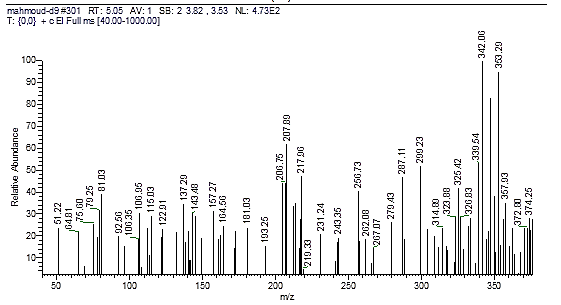

**Mass spectrum of Compound (8)**


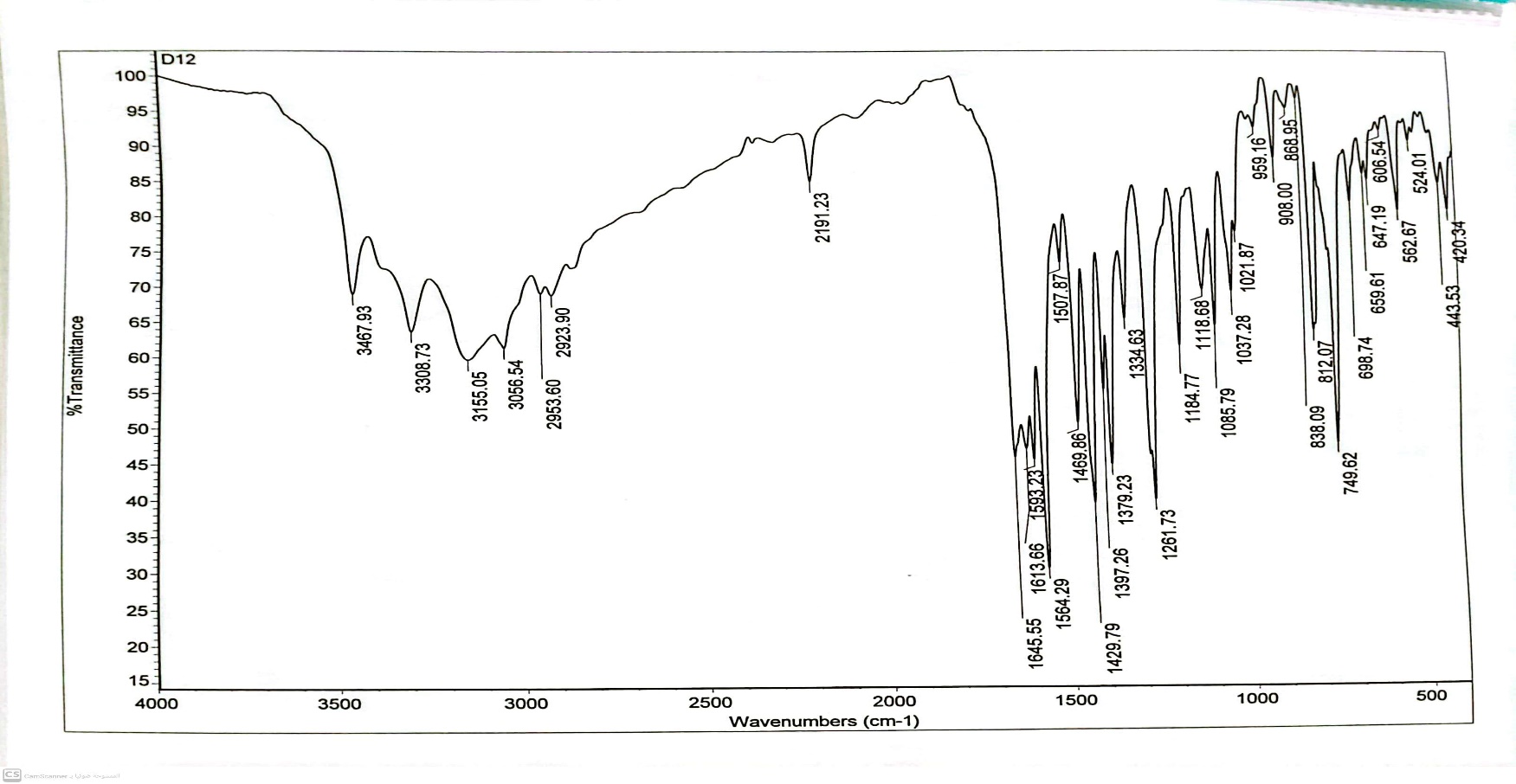

**IR spectrum of compound (9)**


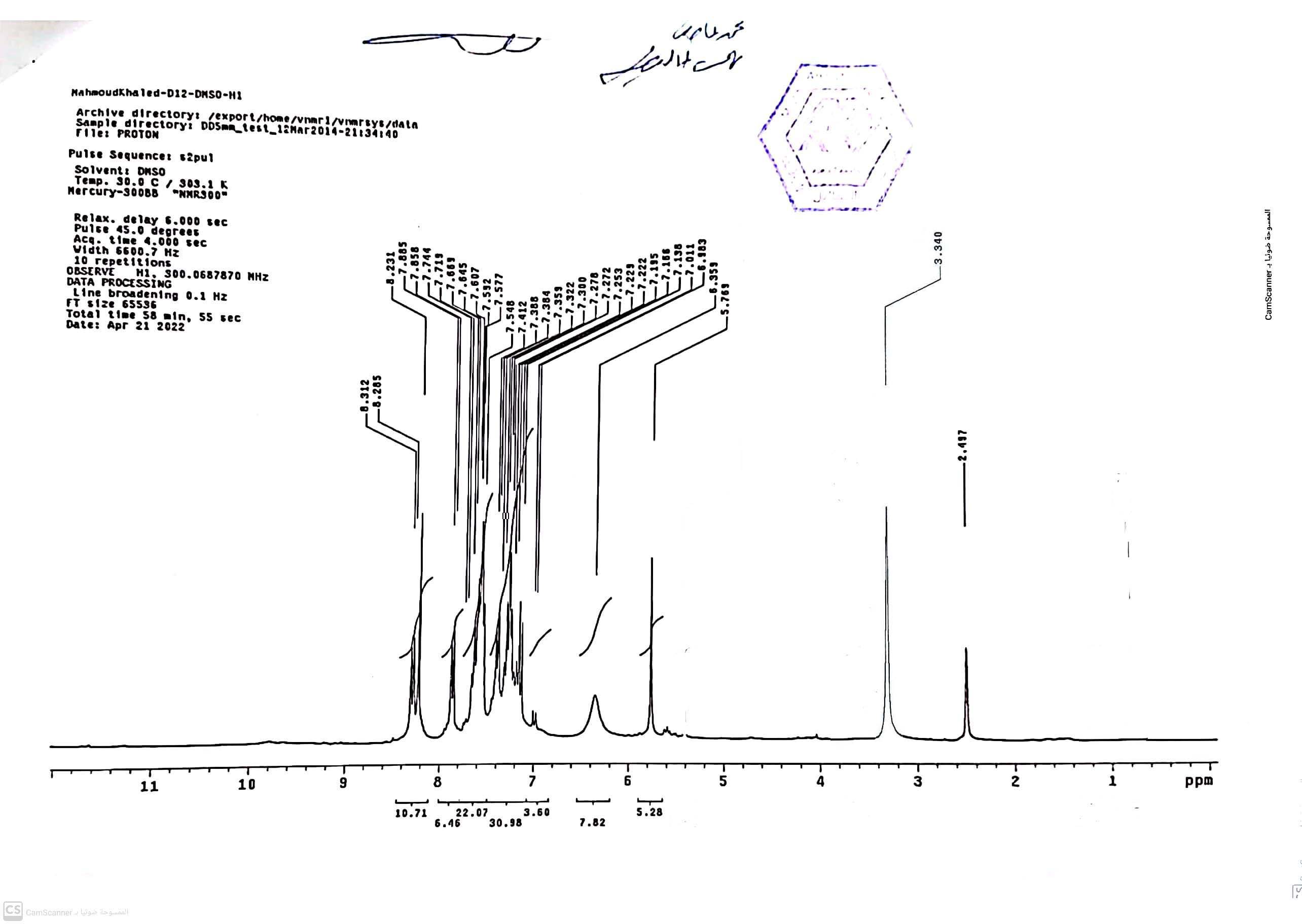

**^1^H-NMR spectrum (DMSO- d_6_) of compound (9)**


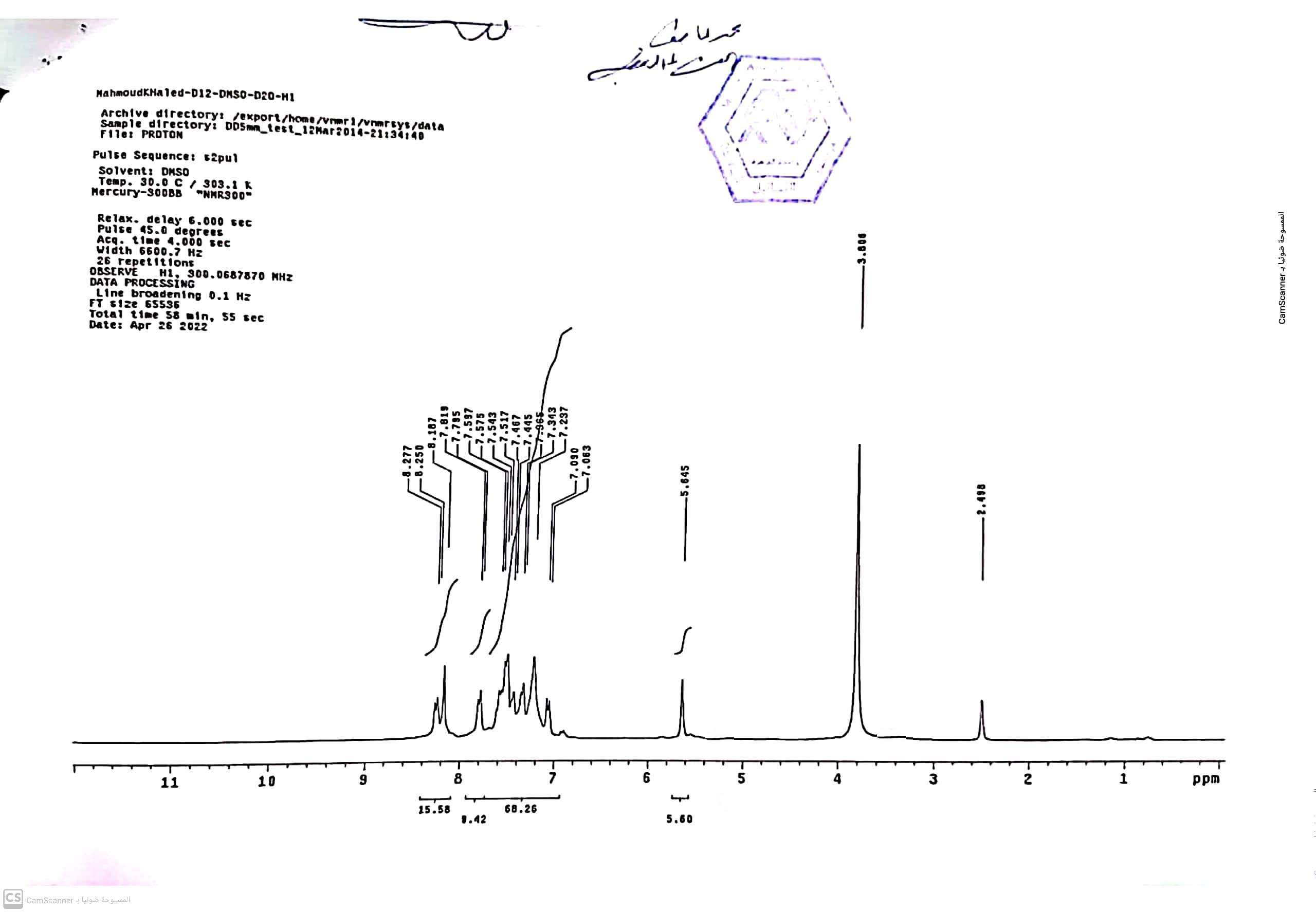

**^1^H-NMR spectrum (DMSO- d_6_+ D_2_O) of Compound (9)**


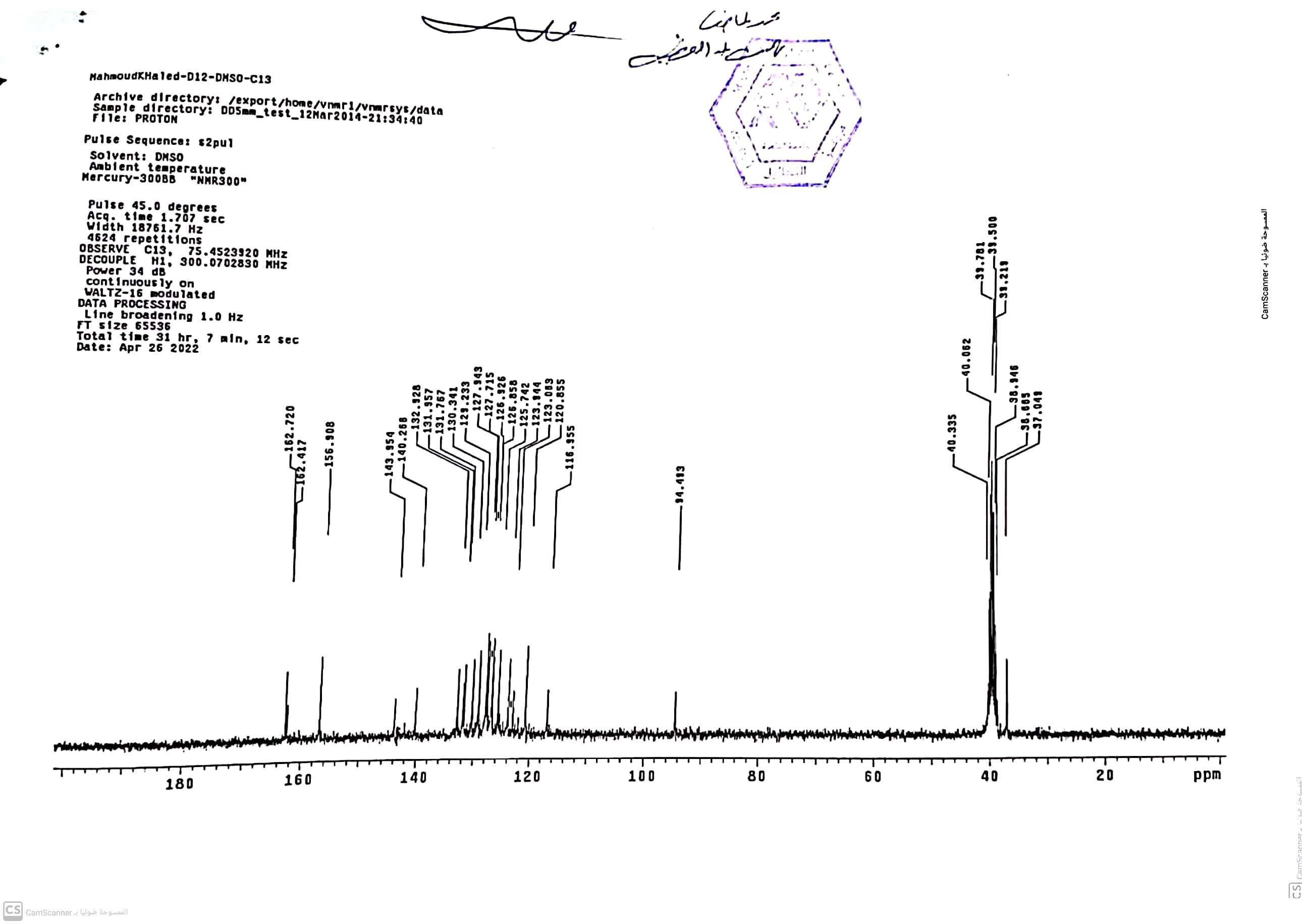

**^13^C -NMR spectrum (DMSO- d_6_) of Compound (9)**


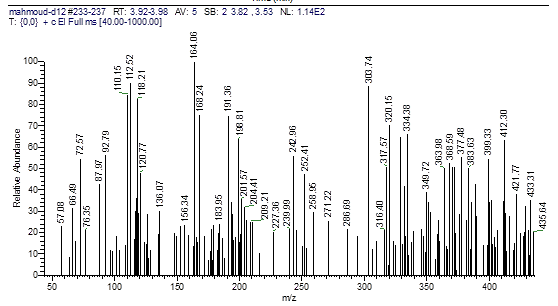

**Mass spectrum of Compound (9)**


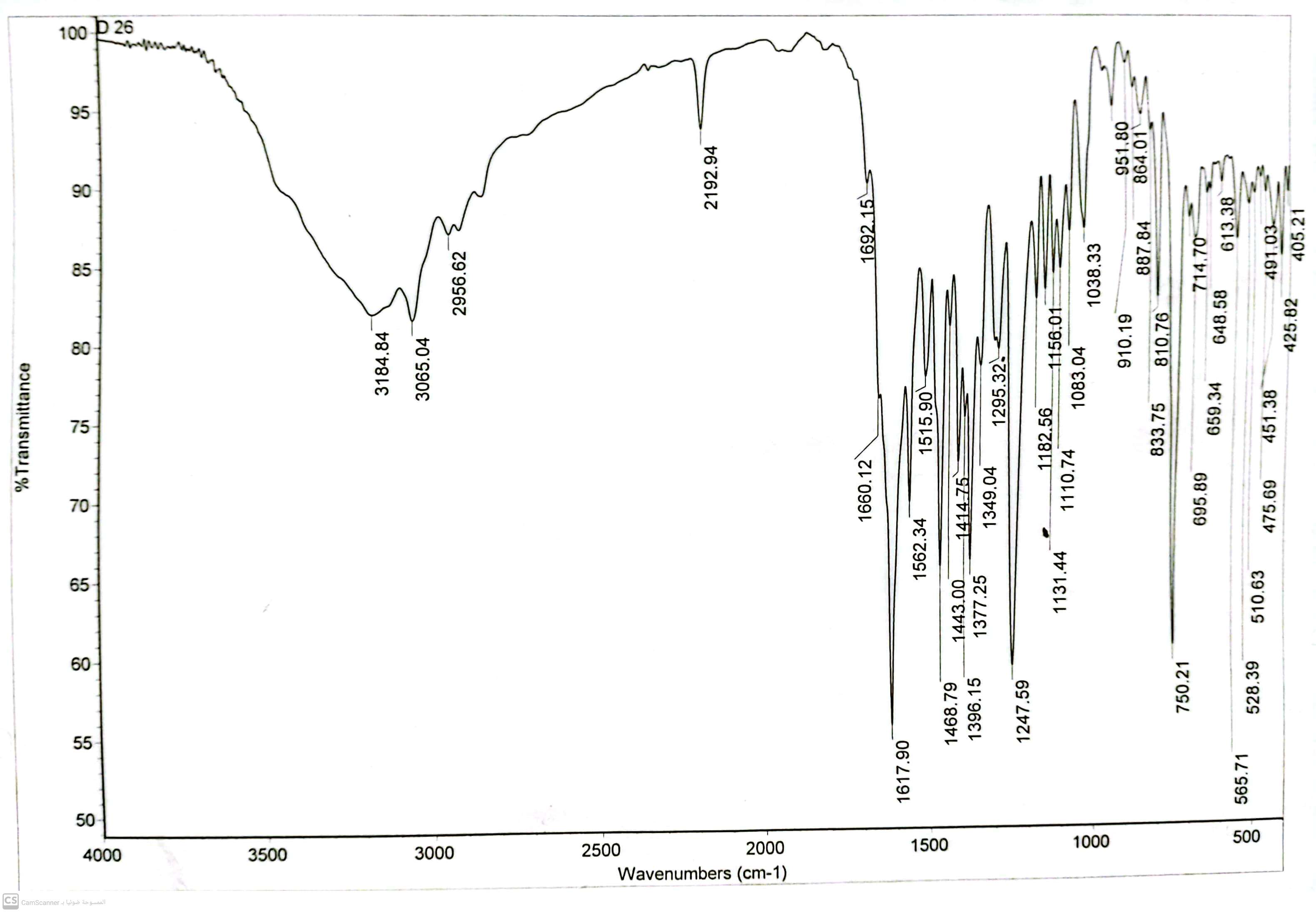

**IR spectrum of compound (10)**


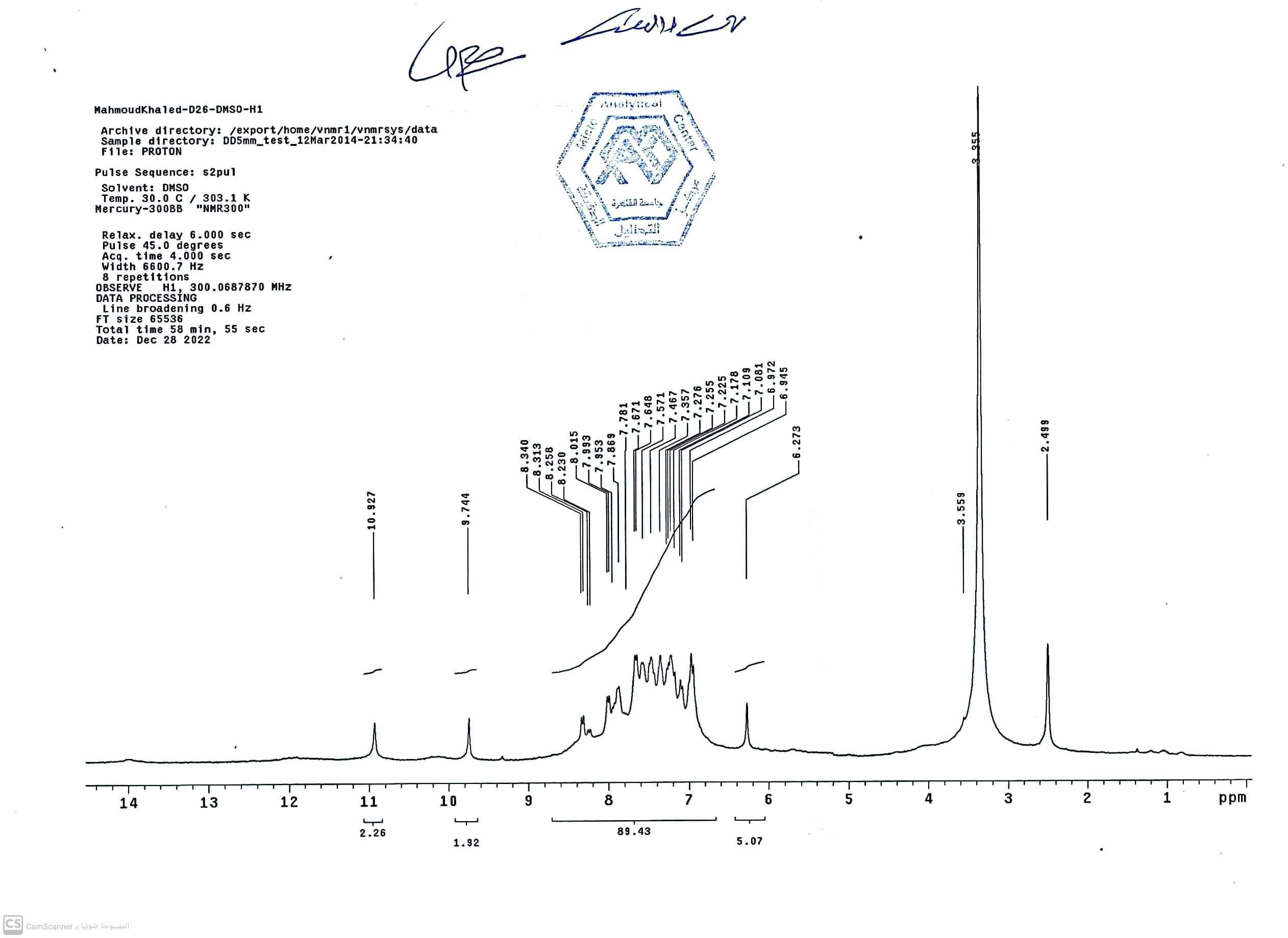

**^1^H-NMR (DMSO- d_6_) of Compound (10)**


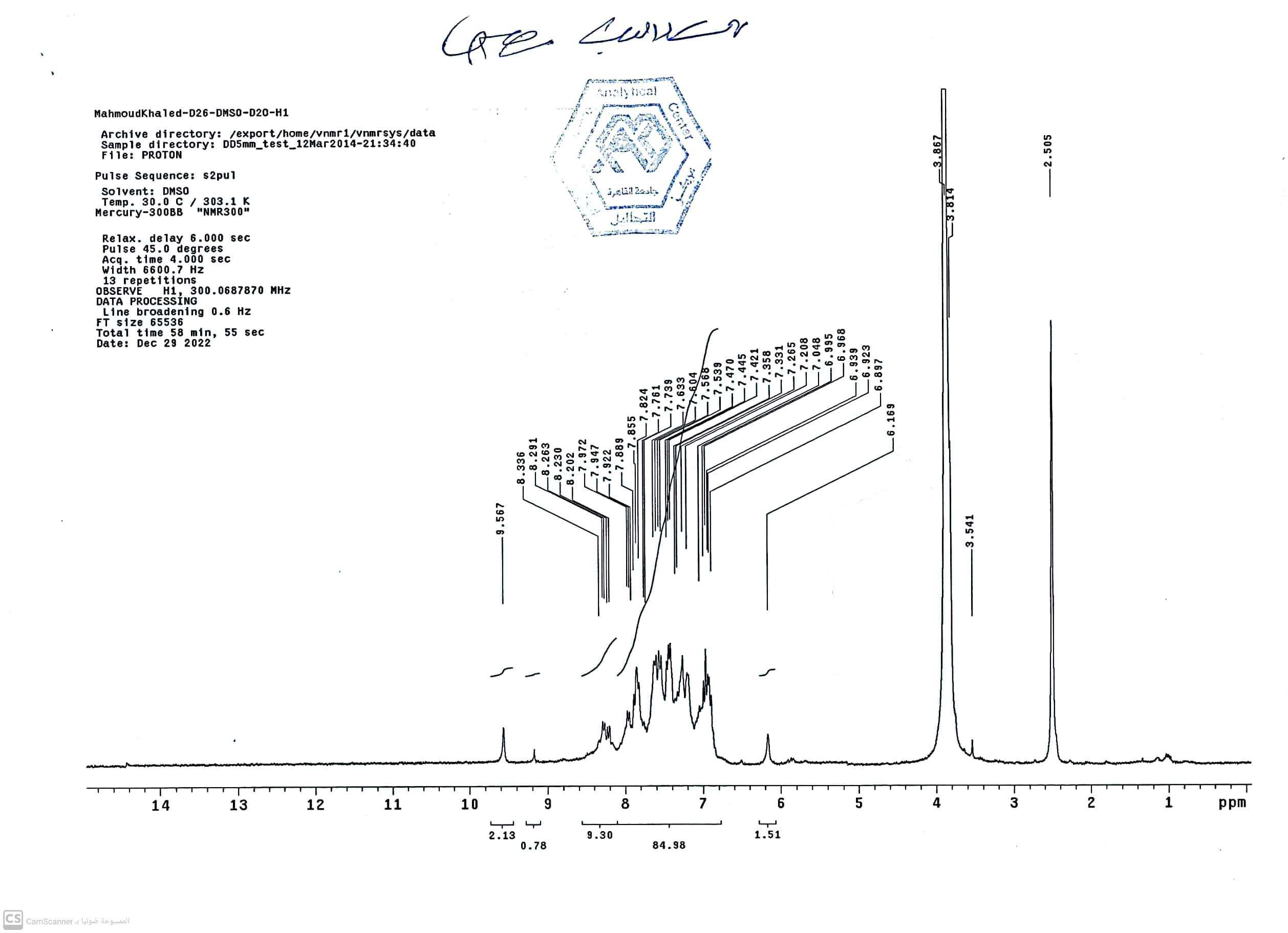

**^1^H-NMR spectrum (DMSO- d_6_ + D_2_O) of Compound (10(**


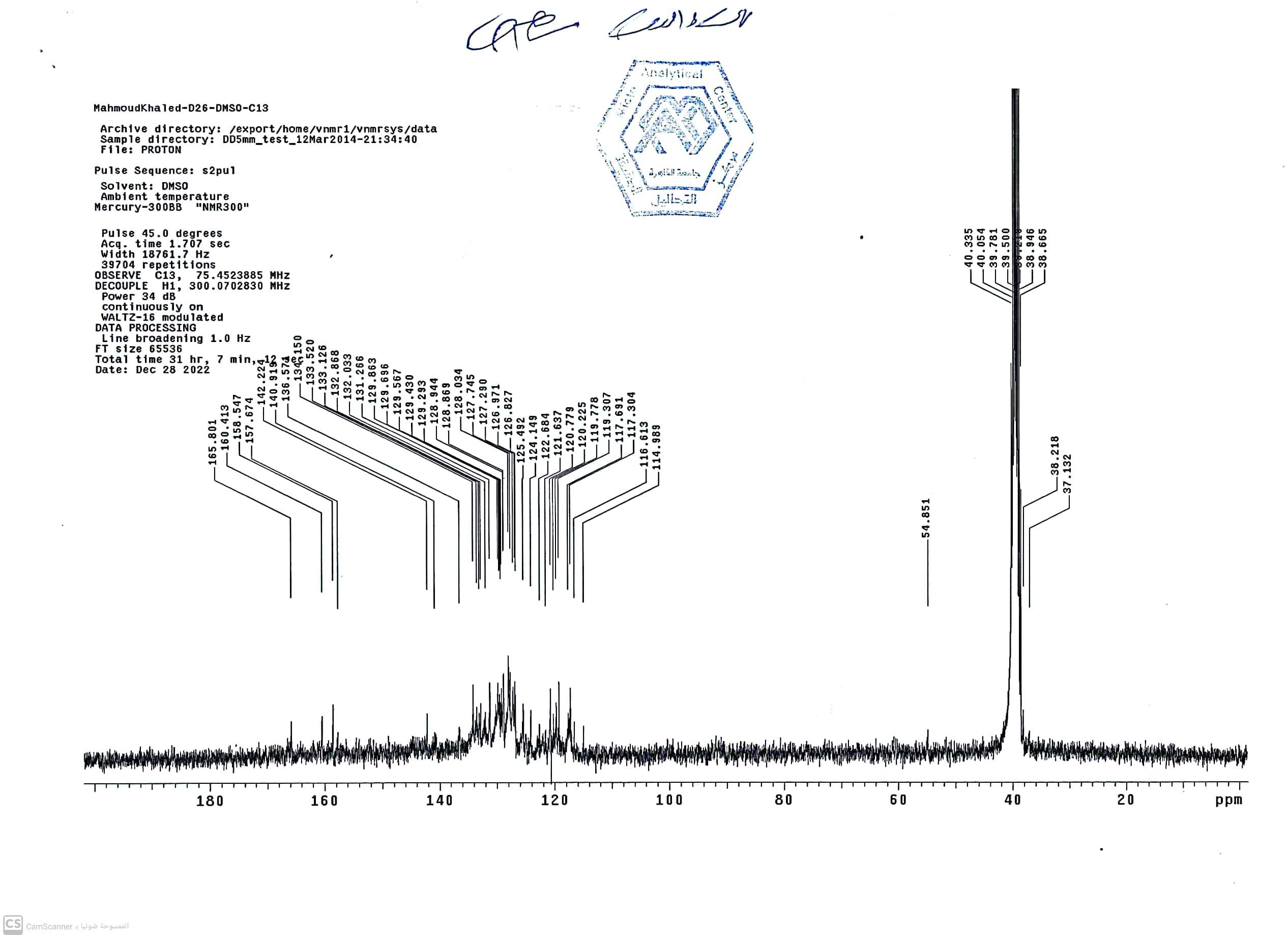

**^13^C-NMR spectrum (DMSO-d_6_) of Compound (10)**


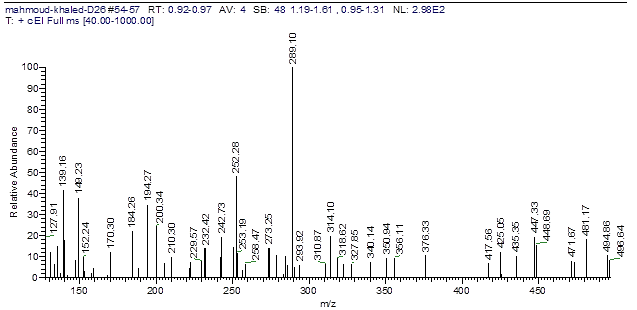

**Mass spectrum of Compound (10)**


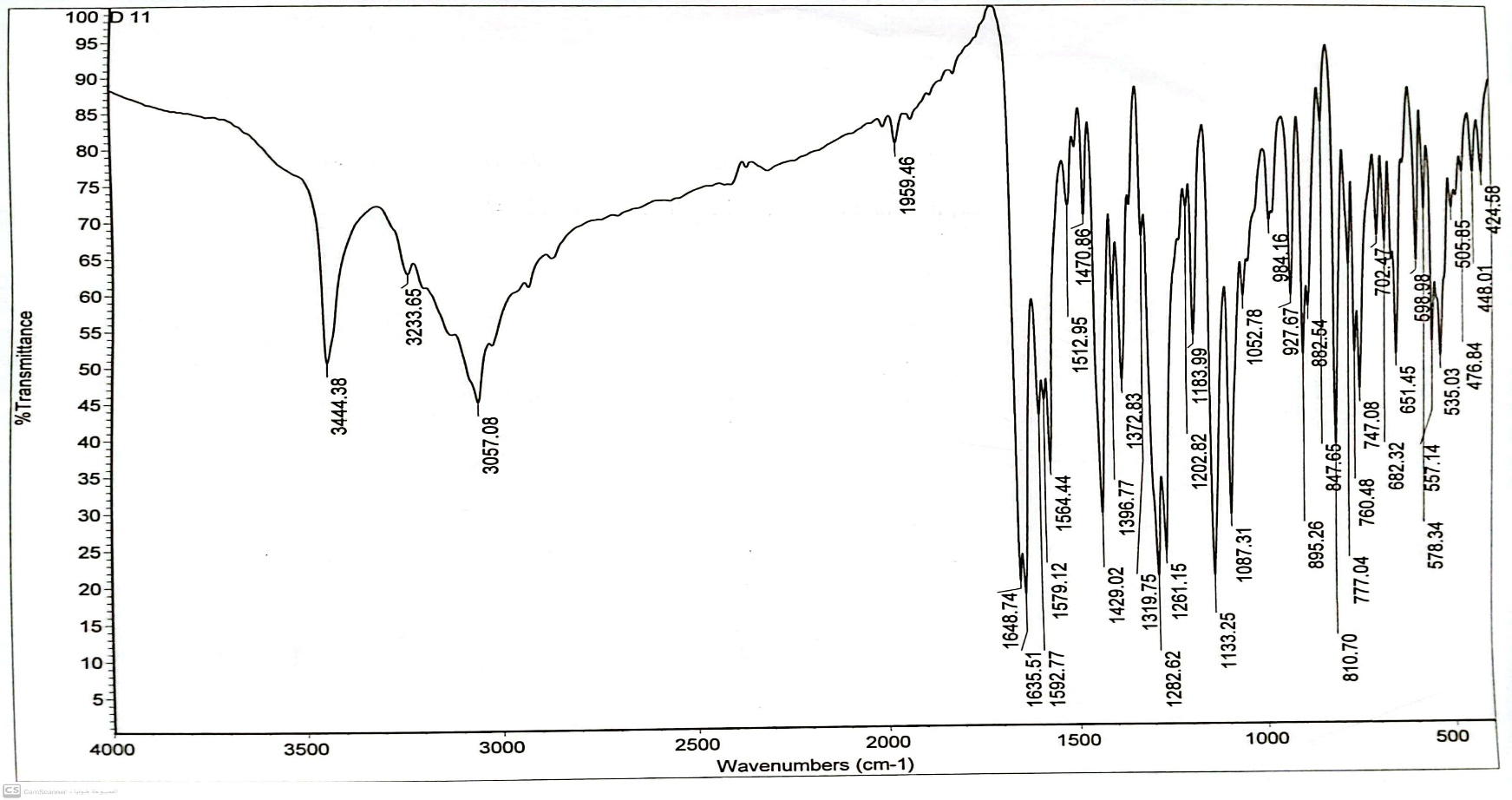

**IR spectrum of compound (11a)**


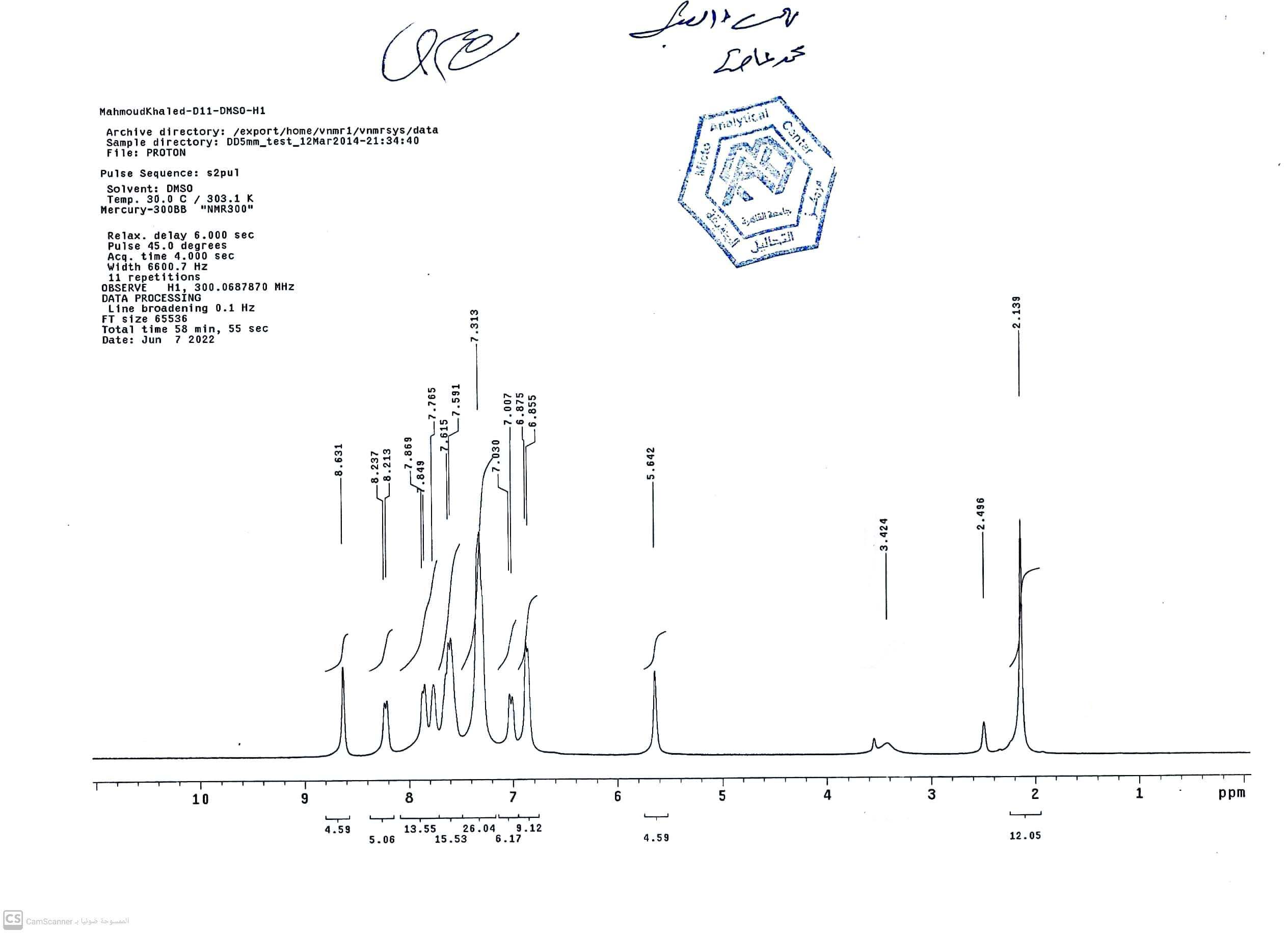

**^1^H-NMR (DMSO- d_6_) of Compound (11a)**


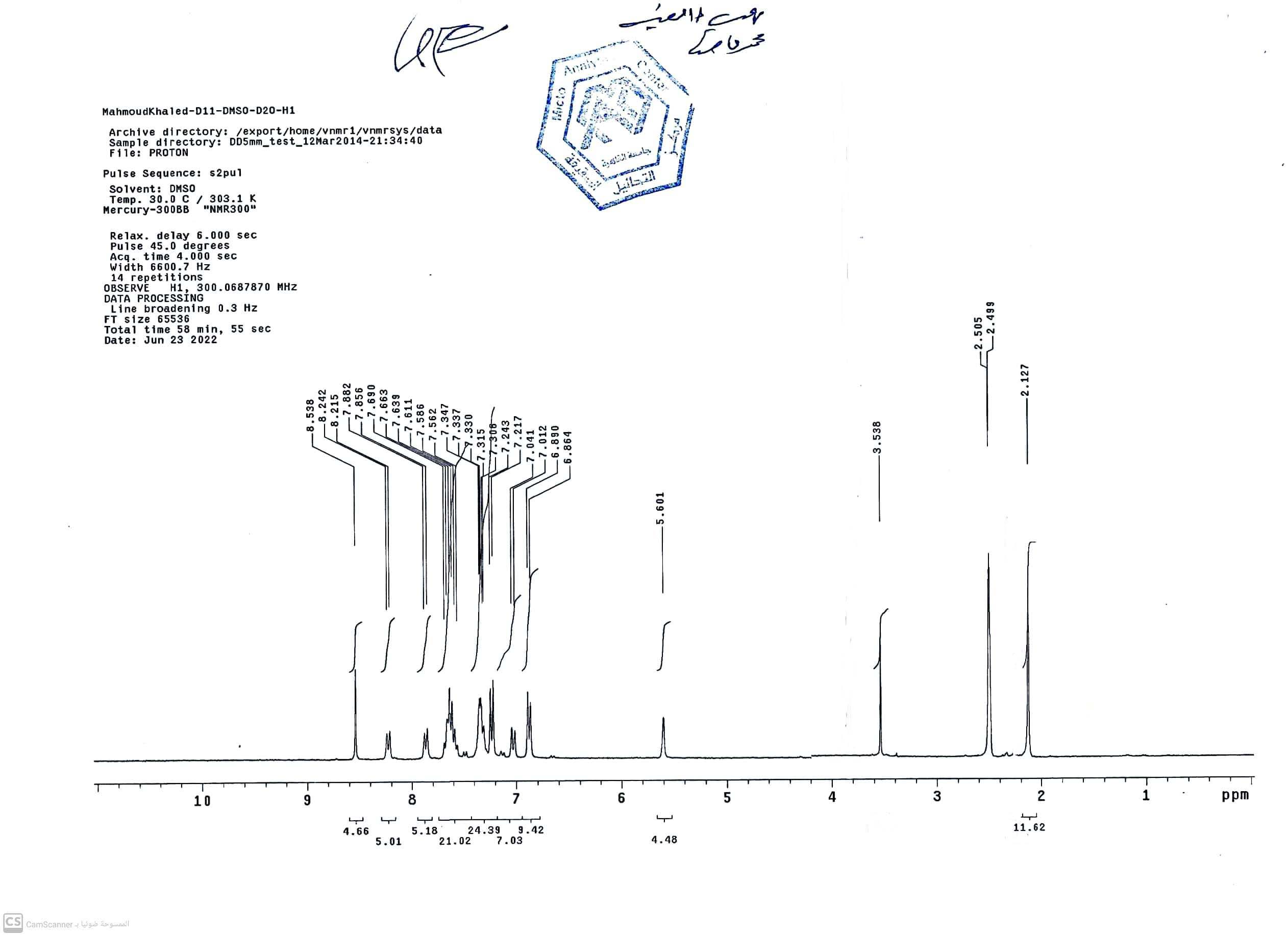

**^1^H-NMR spectrum (DMSO- d_6_ + D_2_O) of Compound (11a(**


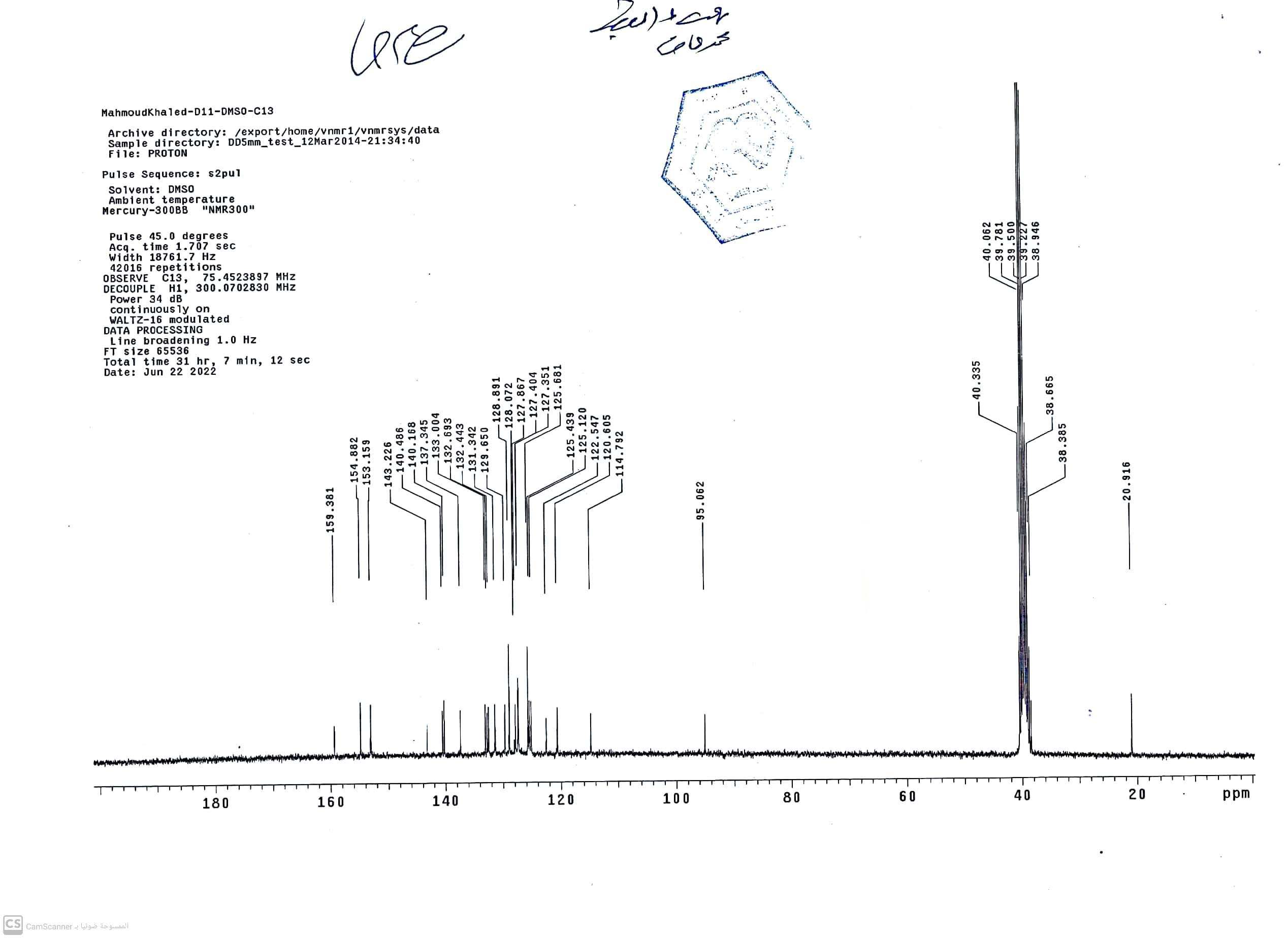

**^13^C -NMR spectrum (DMSO- d_6_) of Compound (11a)**


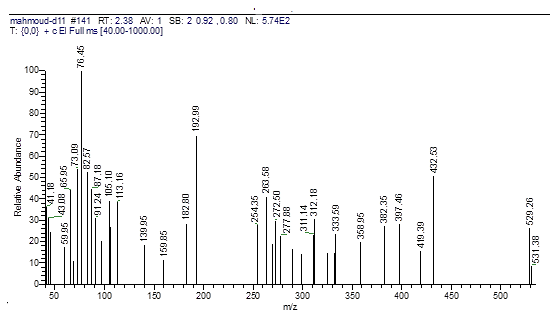

**Mass spectrum of Compound (11a)**


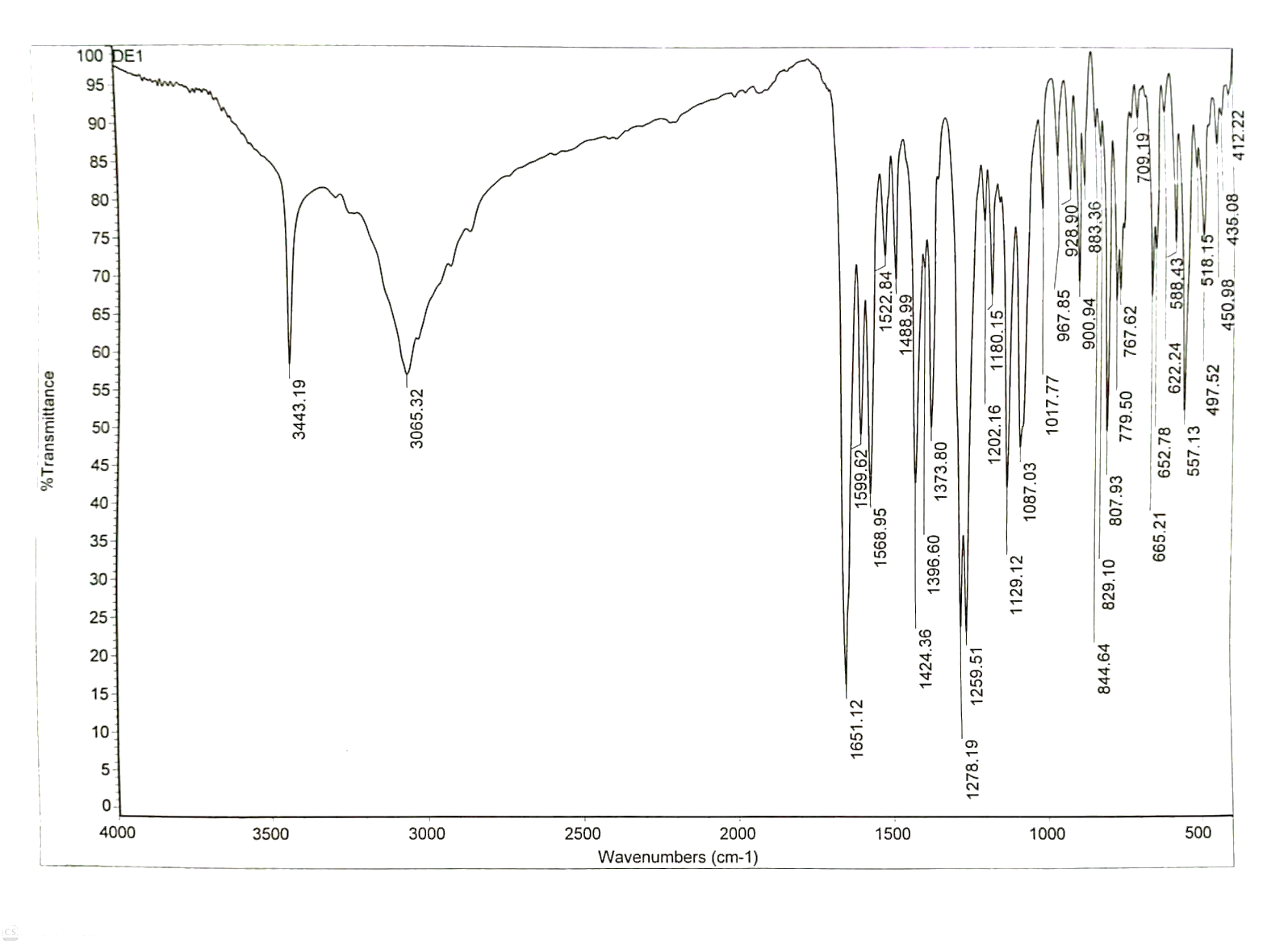

**IR spectrum of compound (11b)**

**
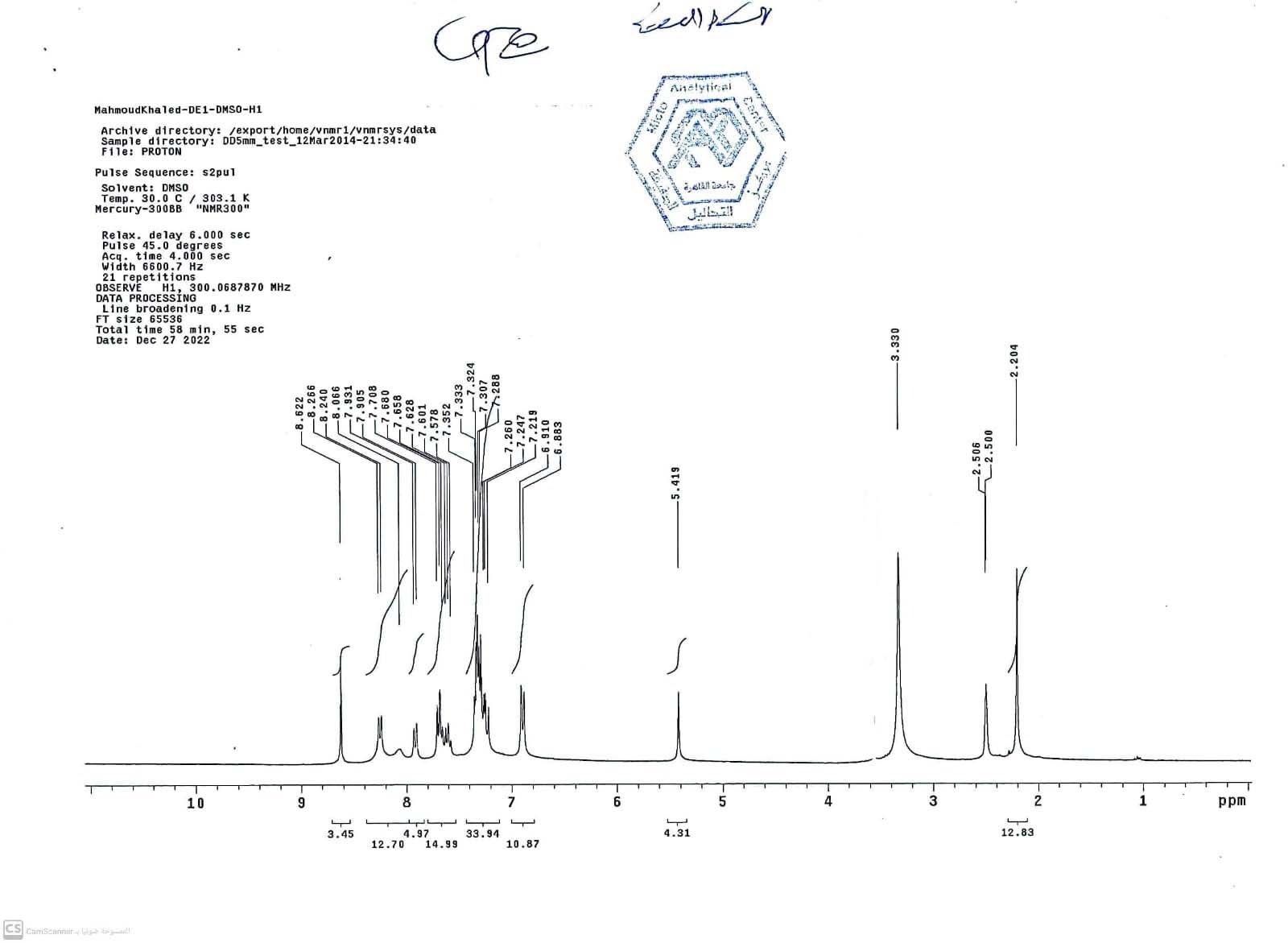
**

**^1^H-NMR (DMSO-d_6_) of Compound (11b)**

**
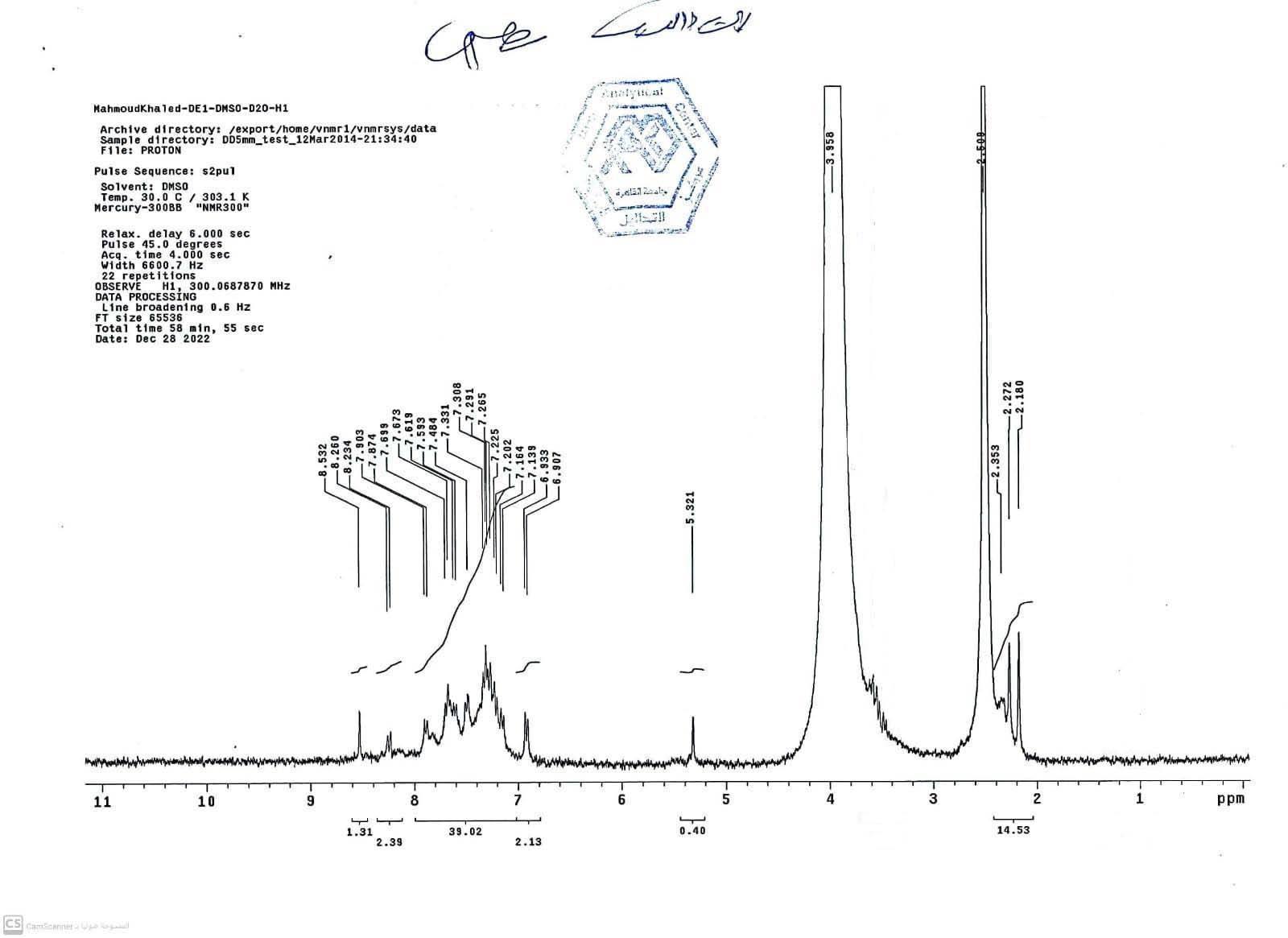
**

**^1^H-NMR spectrum (DMSO-d_6_ + D_2_O) of Compound (11b)**

**
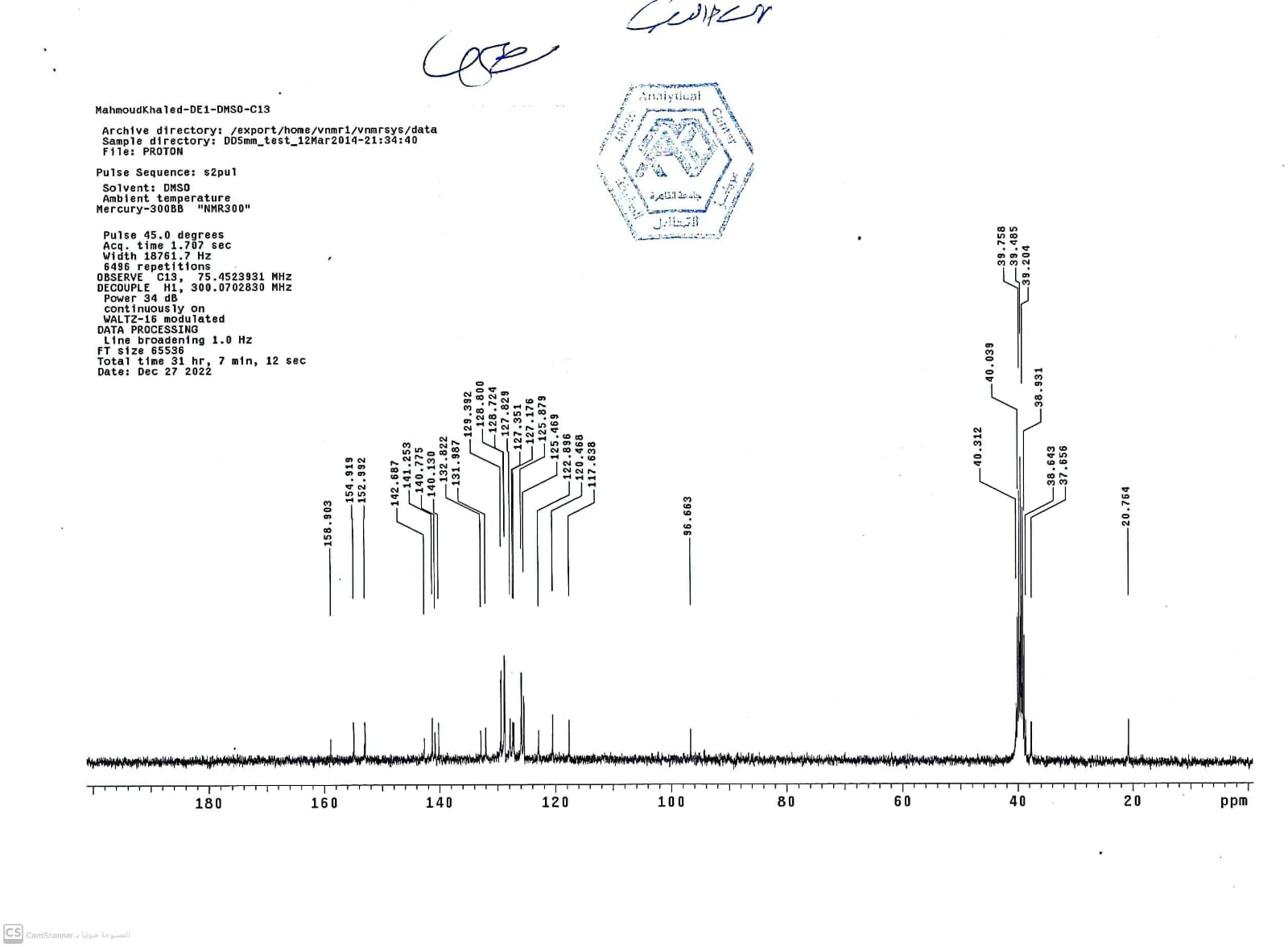
**

**^13^C-NMR spectrum (DMSO-d_6_) of Compound (11b)**


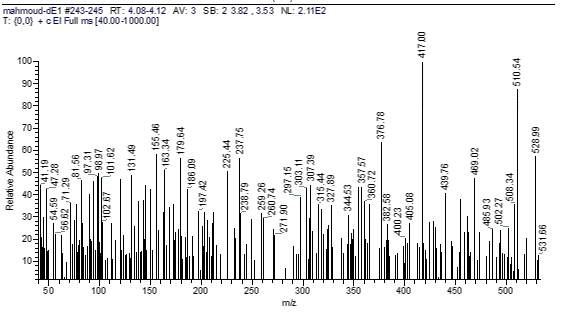

**Mass spectrum of Compound (11b)**


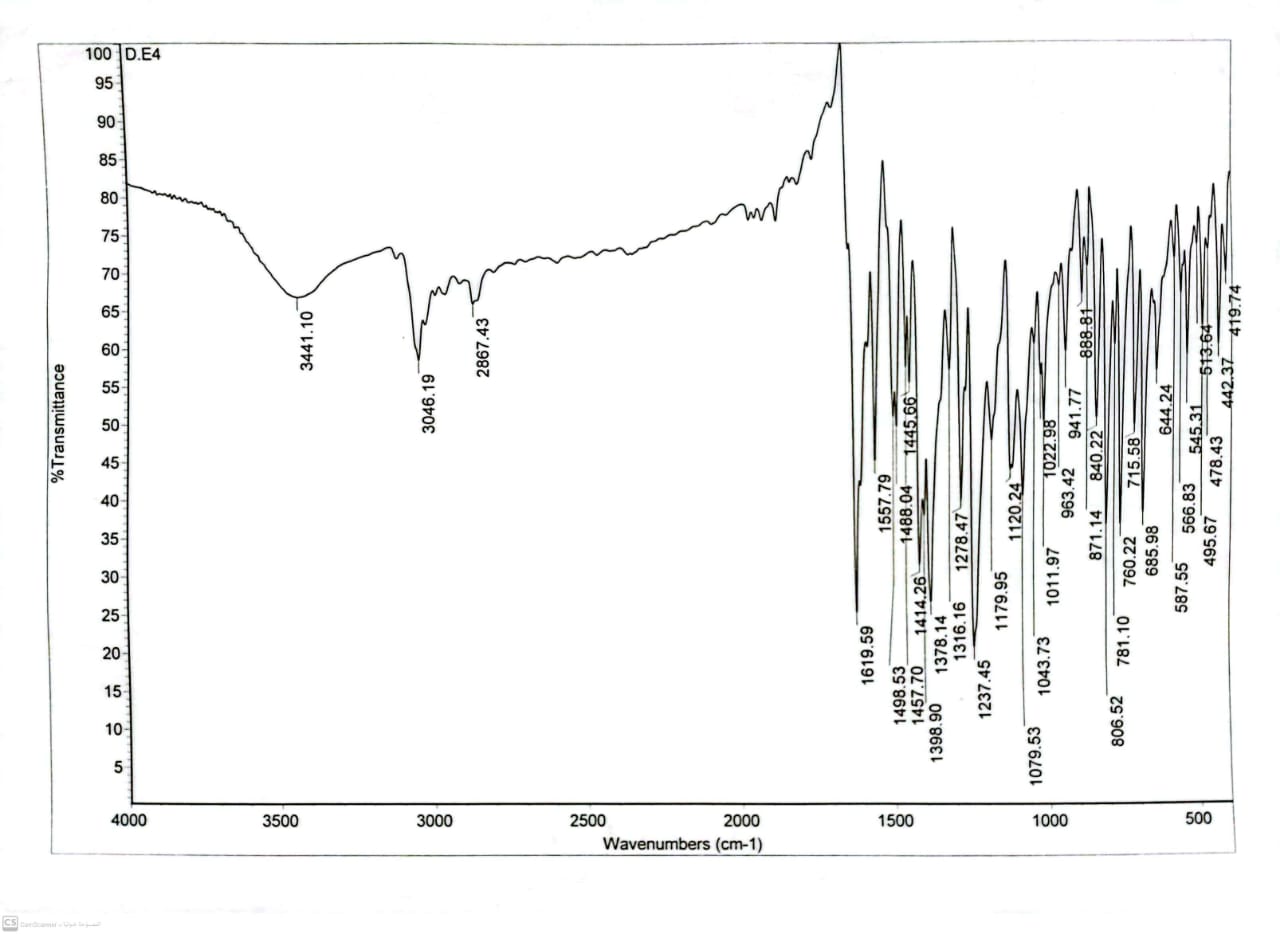

**IR spectrum of compound (12)**


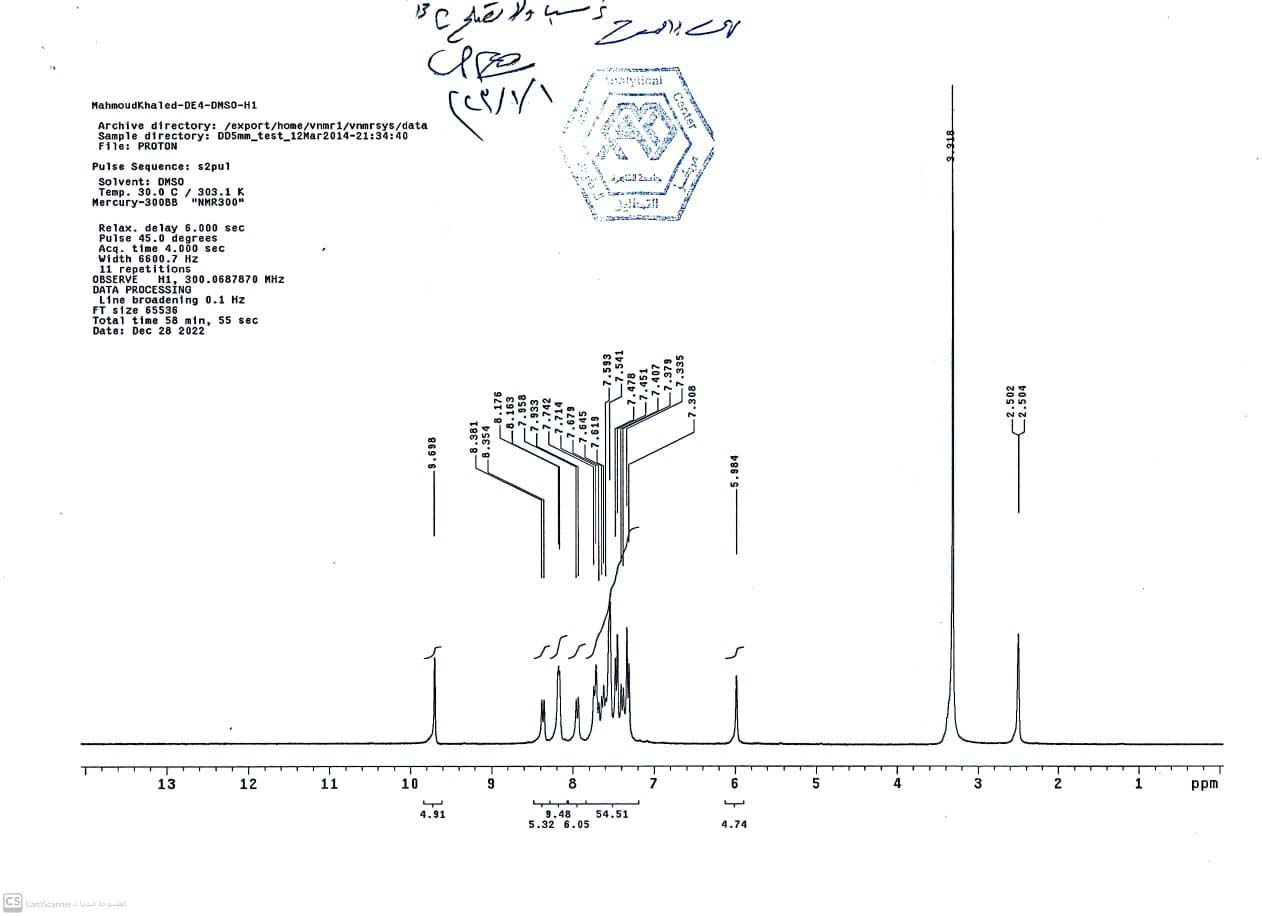

**^1^H-NMR (DMSO-d_6_) of Compound (12)**


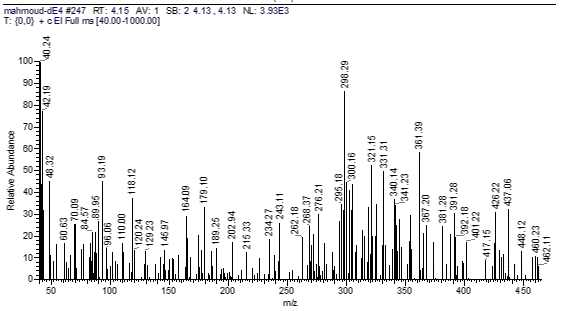

**Mass spectrum of Compound (12)**


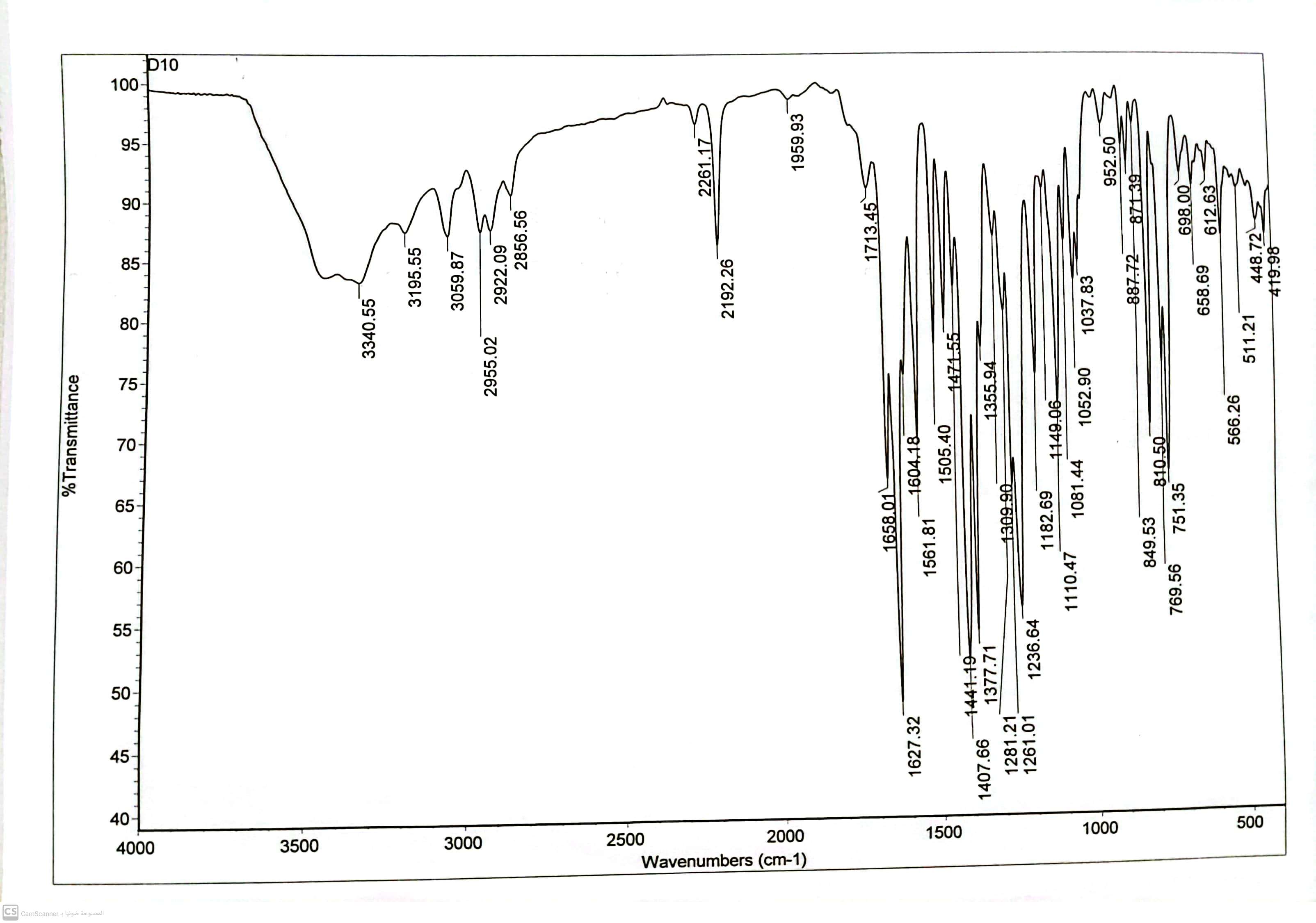

**IR spectrum of compound (13)**


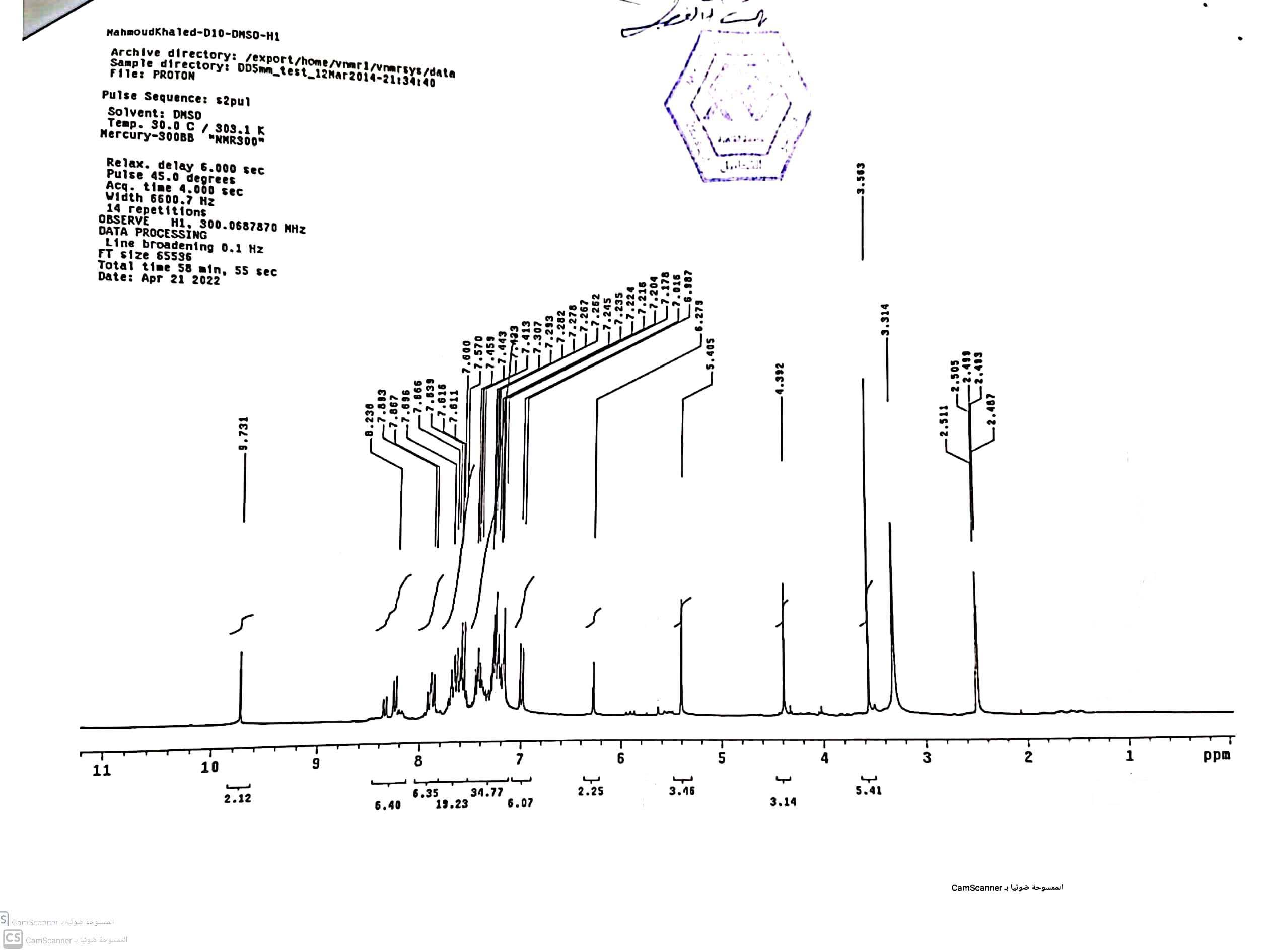

**^1^H-NMR (DMSO- d_6_) of Compound (13)**


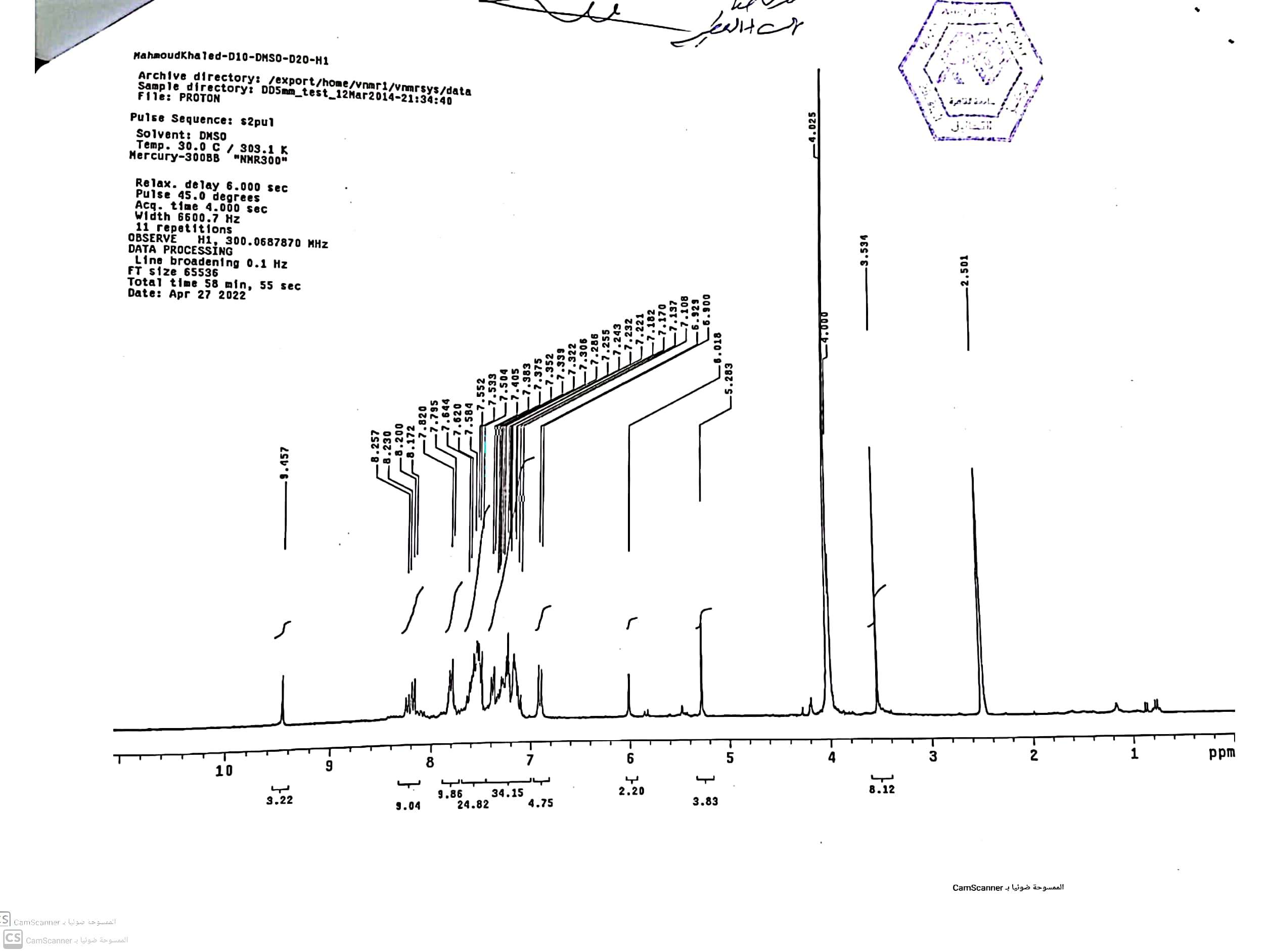

**^1^H-NMR spectrum (DMSO-d_6_ + D_2_O) of Compound (13)**


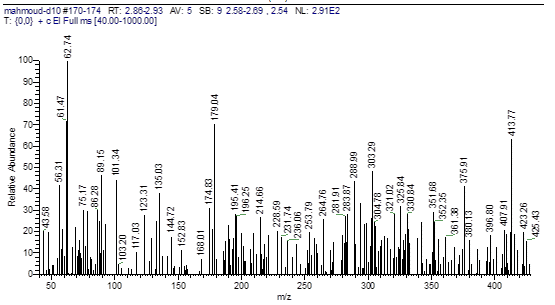

**Mass spectrum of Compound (13)**


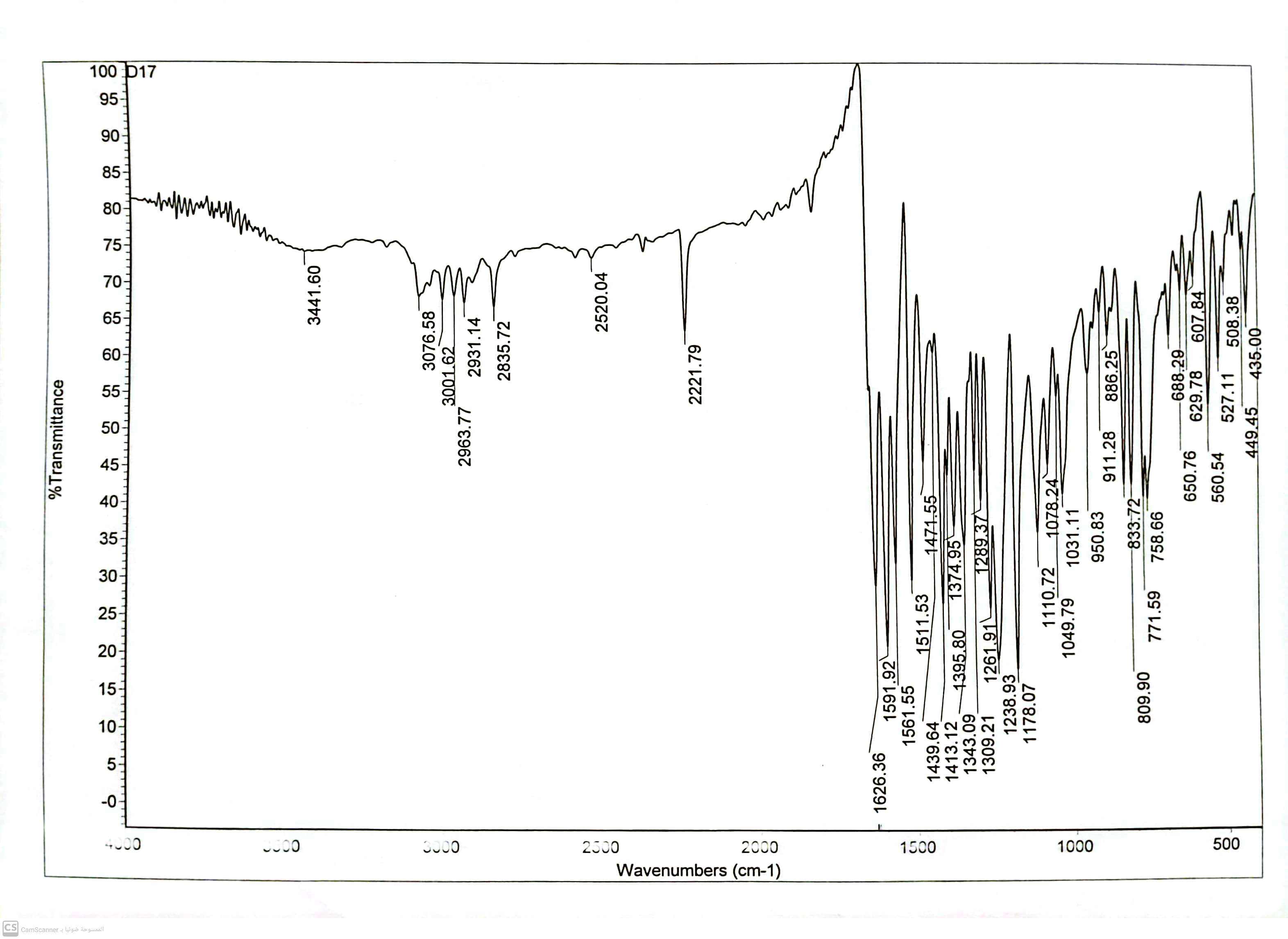

**IR spectrum of compound (14)**


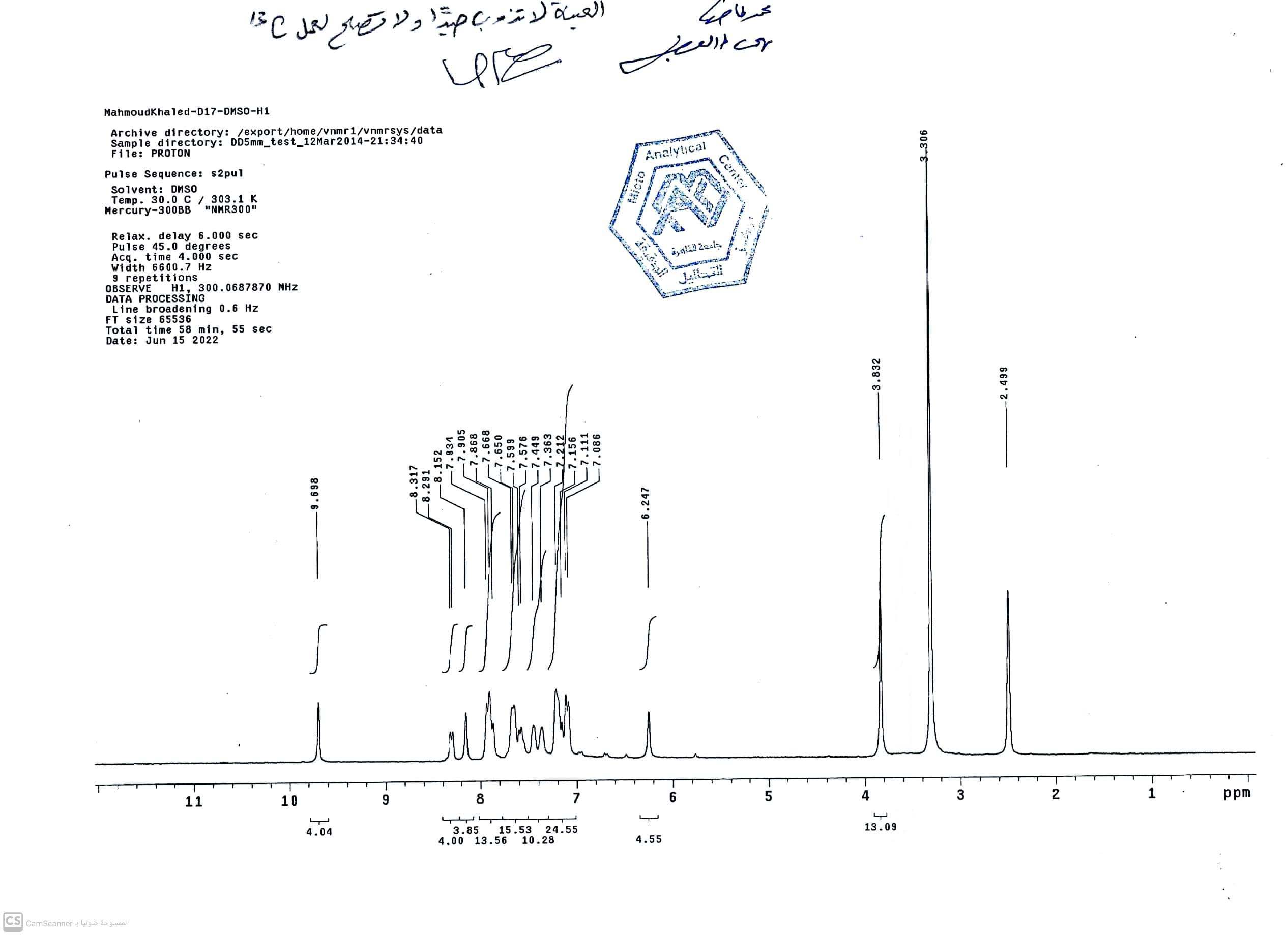

**^1^H-NMR (DMSO-d_6_) of Compound (14)**


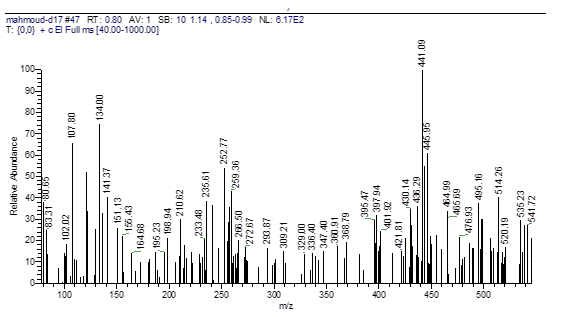

**Mass spectrum of Compound (14)**


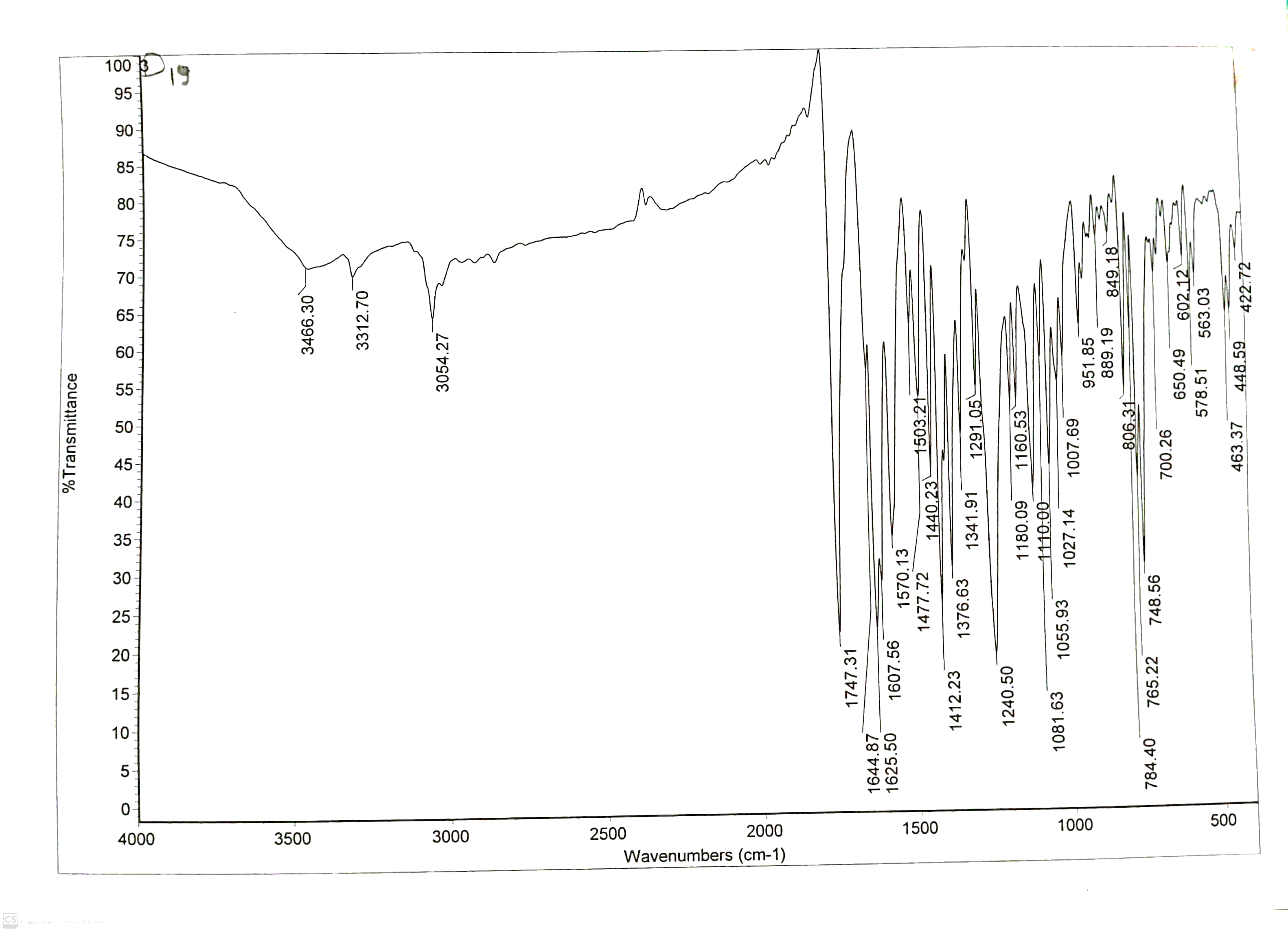

**IR spectrum of compound (15)**


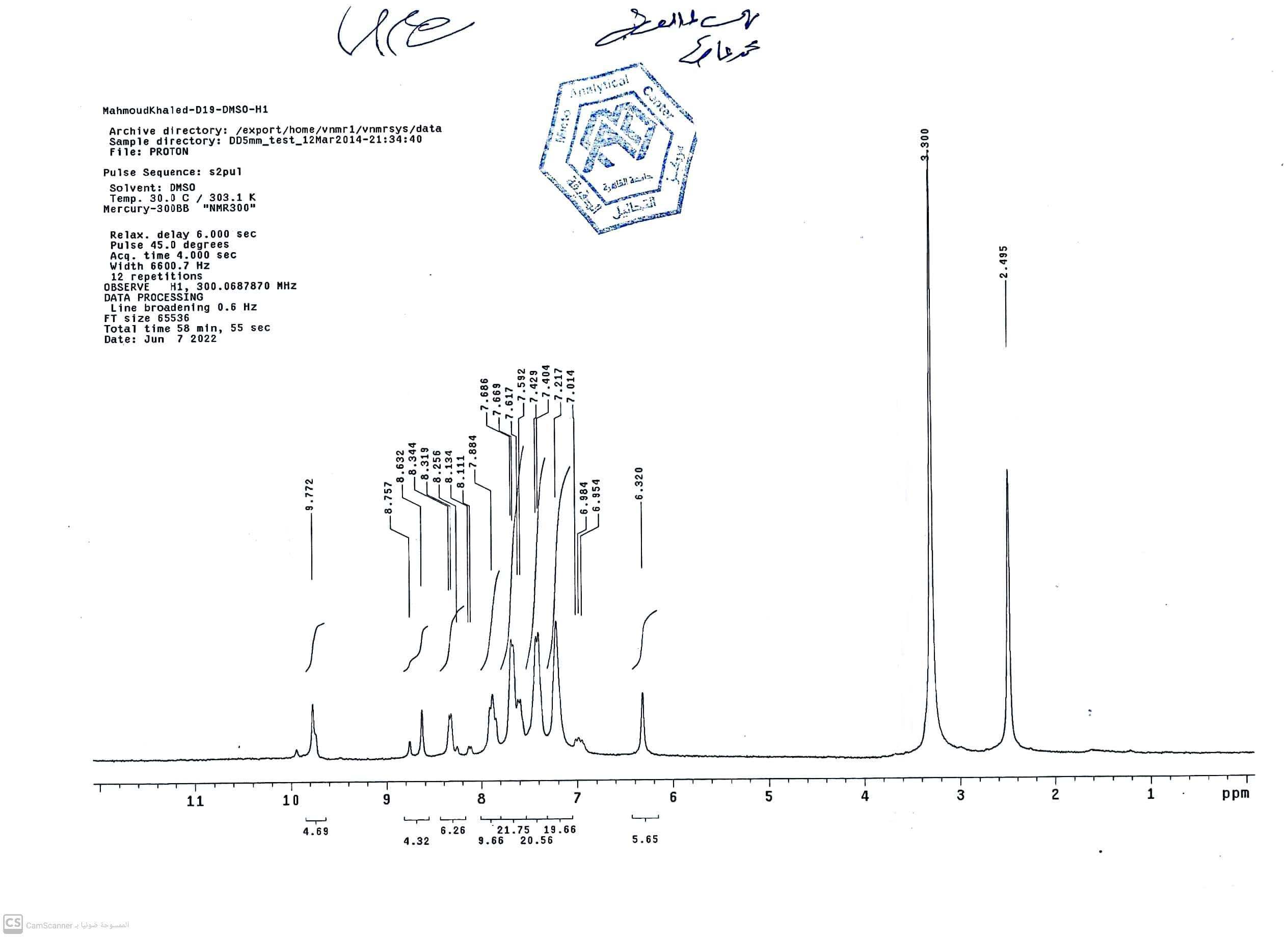

**^1^H-NMR (DMSO-d_6_) of Compound (15)**

**
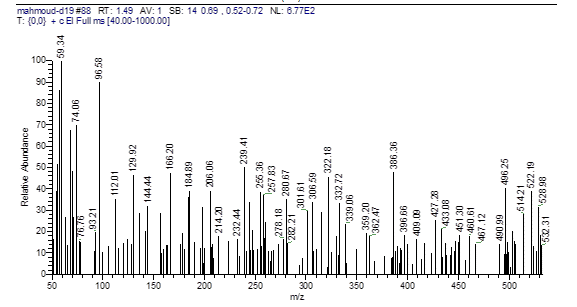
**

**Mass spectrum of Compound (15)**


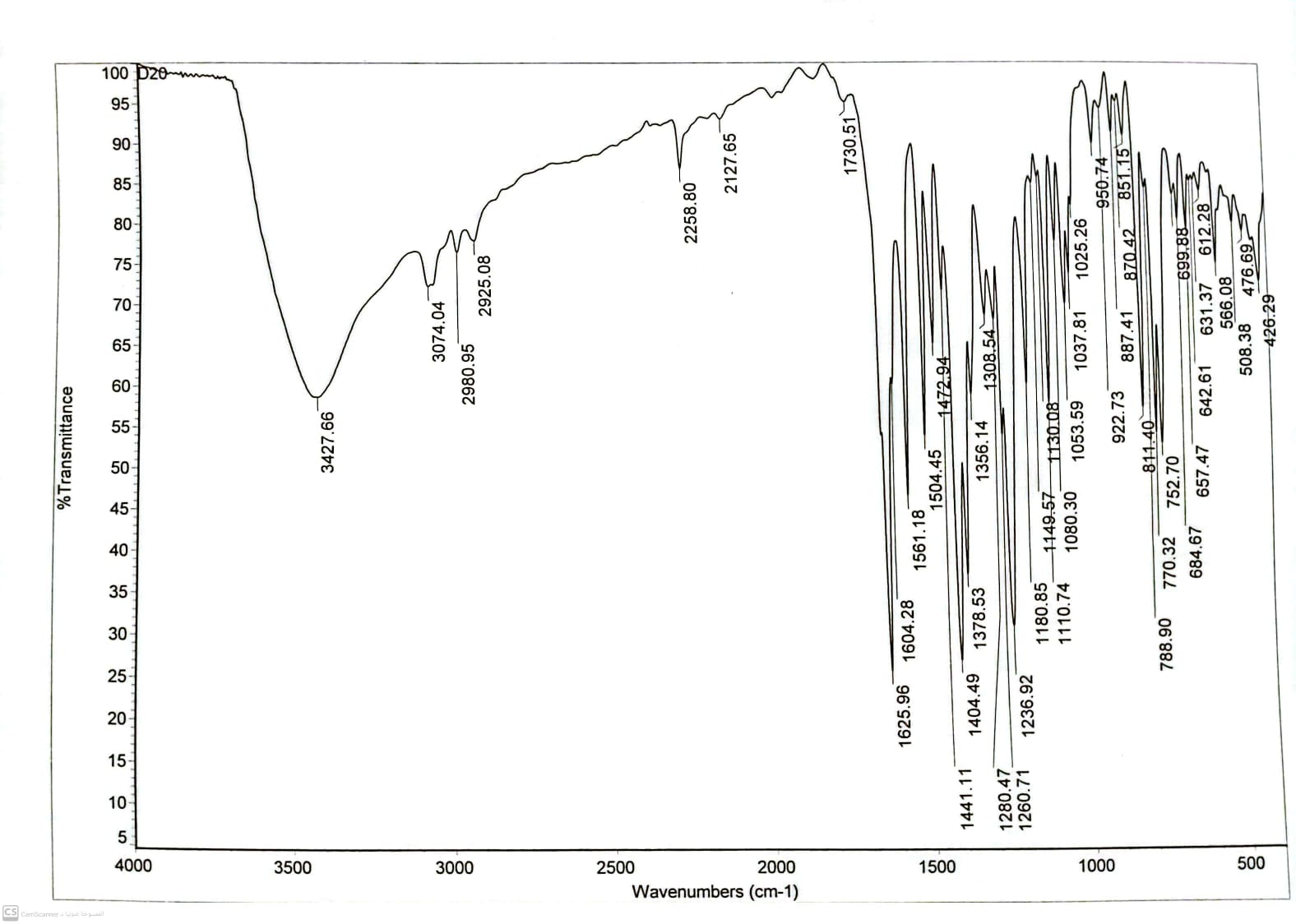

**IR spectrum of compound (16)**


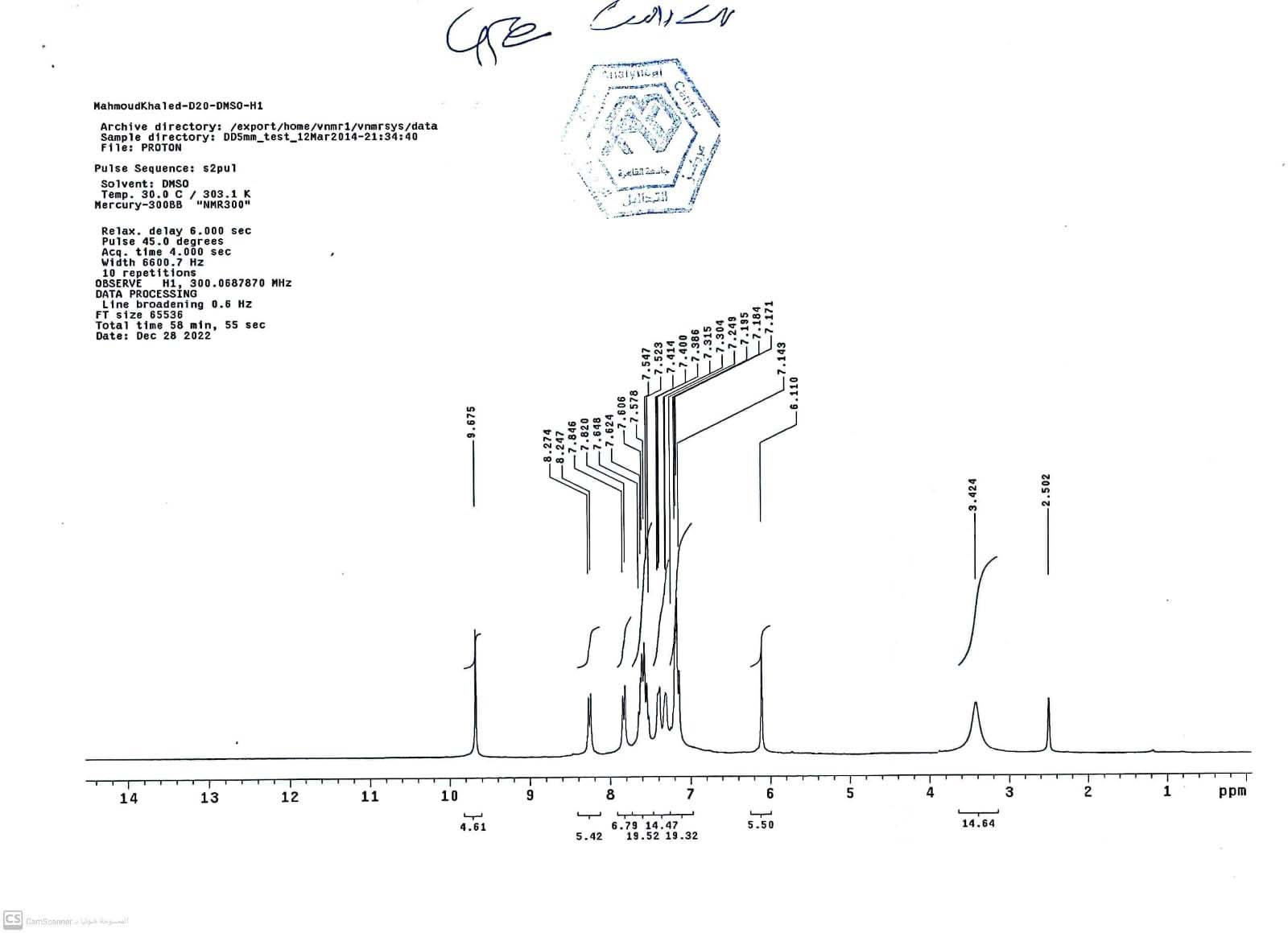

**^1^H-NMR (DMSO-d_6_) of Compound (16)**


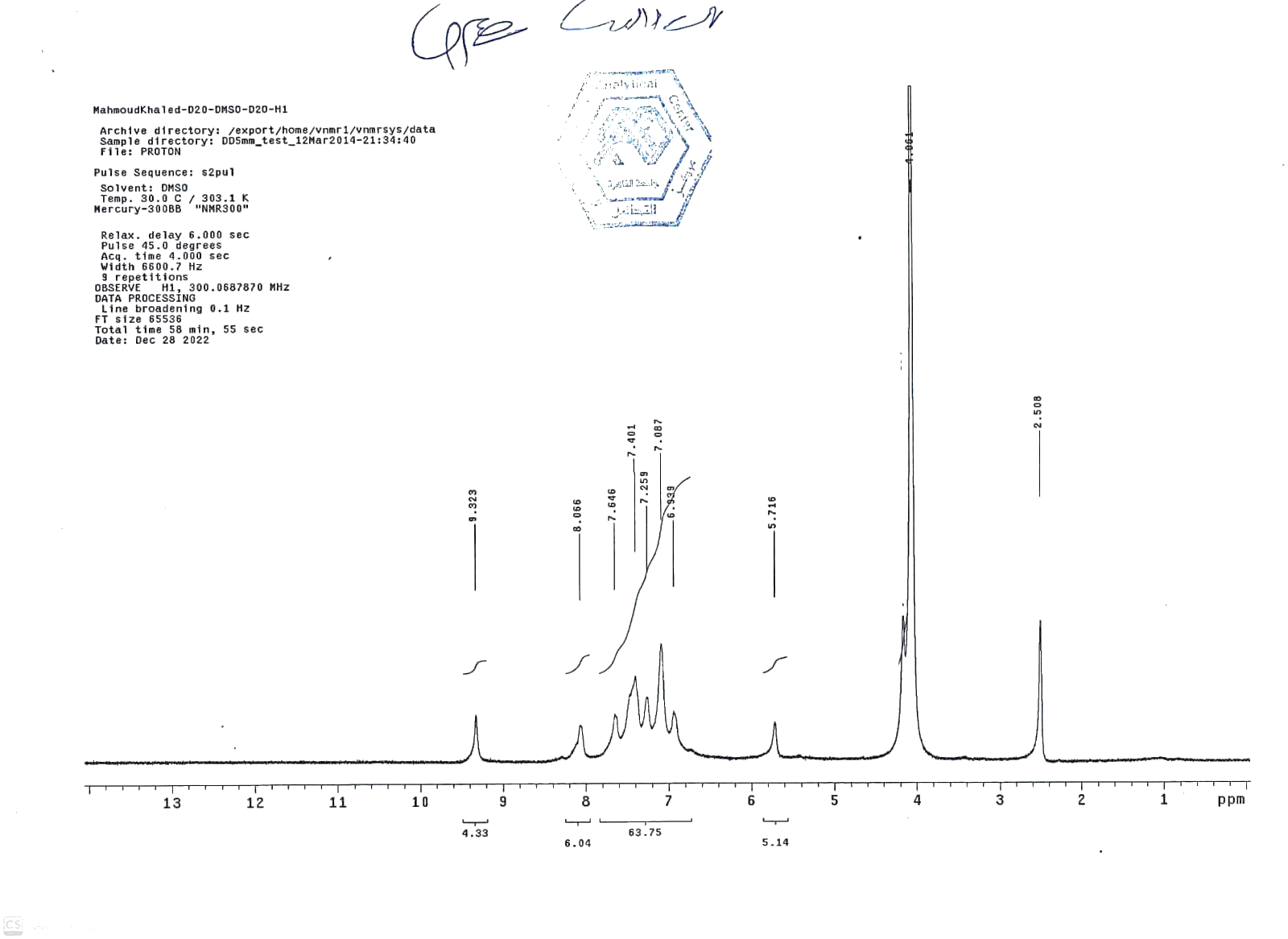

**^1^H-NMR spectrum (DMSO- d_6_ + D_2_O) of Compound (16)**


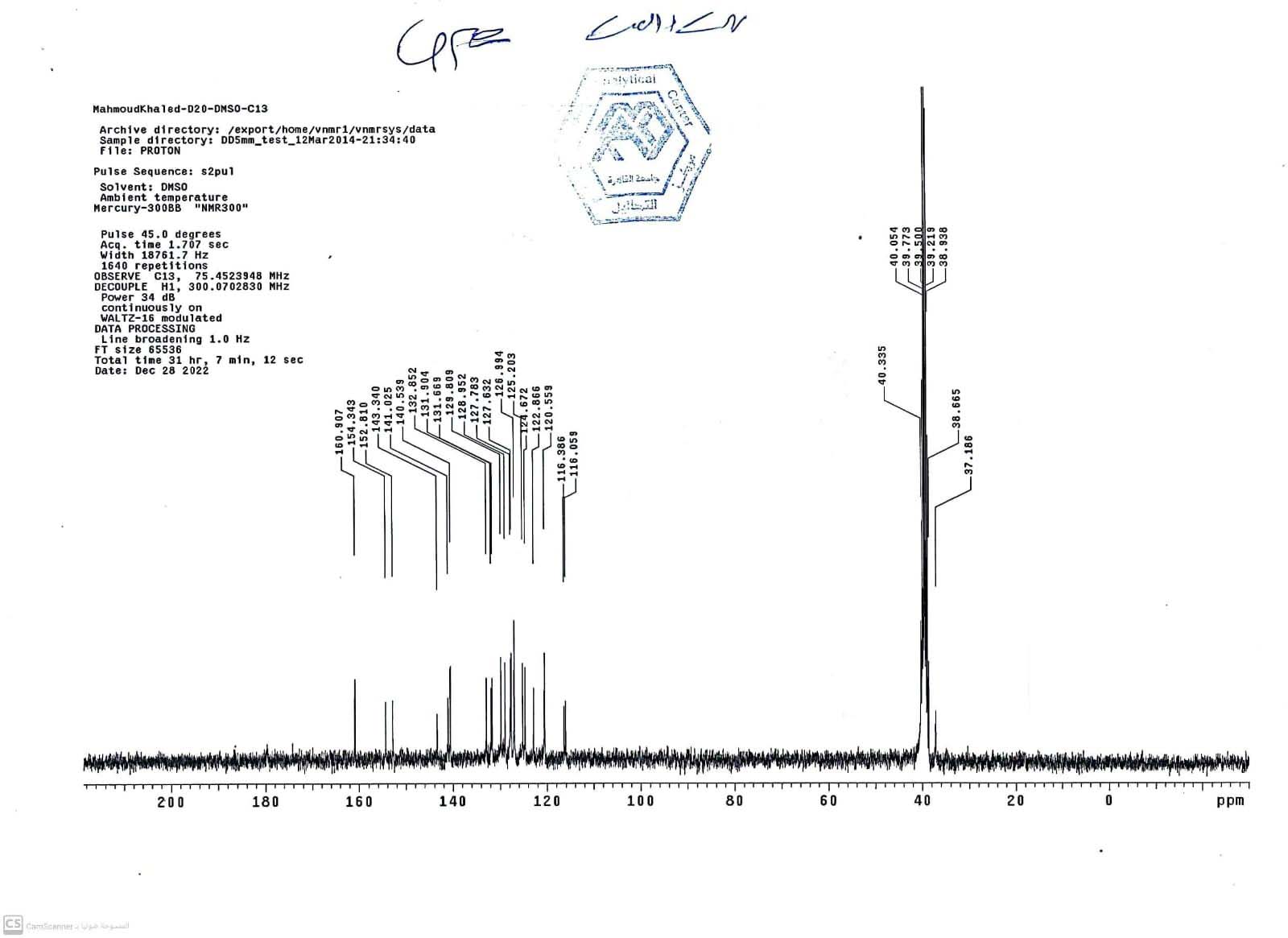

**^13^C-NMR spectrum (DMSO-d_6_) of Compound (16)**


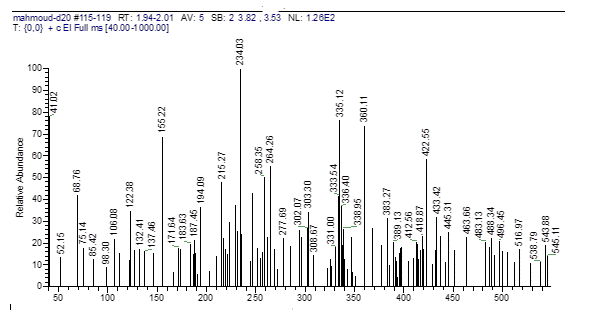

**Mass spectrum of Compound (16)**
